# Search for the Standard Model Higgs boson produced in association with top quarks and decaying into $\varvec{b\bar{b}}$ in $\varvec{pp}$ collisions at $\sqrt{\mathbf{s}}= \varvec{8{{\,\mathrm TeV}}}$ with the ATLAS detector

**DOI:** 10.1140/epjc/s10052-015-3543-1

**Published:** 2015-07-29

**Authors:** G. Aad, B. Abbott, J. Abdallah, O. Abdinov, R. Aben, M. Abolins, O. S. AbouZeid, H. Abramowicz, H. Abreu, R. Abreu, Y. Abulaiti, B. S. Acharya, L. Adamczyk, D. L. Adams, J. Adelman, S. Adomeit, T. Adye, A. A. Affolder, T. Agatonovic-Jovin, J. A. Aguilar-Saavedra, M. Agustoni, S. P. Ahlen, F. Ahmadov, G. Aielli, H. Akerstedt, T. P. A. Åkesson, G. Akimoto, A. V. Akimov, G. L. Alberghi, J. Albert, S. Albrand, M. J. Alconada Verzini, M. Aleksa, I. N. Aleksandrov, C. Alexa, G. Alexander, T. Alexopoulos, M. Alhroob, G. Alimonti, L. Alio, J. Alison, S. P. Alkire, B. M. M. Allbrooke, P. P. Allport, A. Aloisio, A. Alonso, F. Alonso, C. Alpigiani, A. Altheimer, B. Alvarez Gonzalez, D. Álvarez Piqueras, M. G. Alviggi, K. Amako, Y. Amaral Coutinho, C. Amelung, D. Amidei, S. P. Amor Dos Santos, A. Amorim, S. Amoroso, N. Amram, G. Amundsen, C. Anastopoulos, L. S. Ancu, N. Andari, T. Andeen, C. F. Anders, G. Anders, K. J. Anderson, A. Andreazza, V. Andrei, S. Angelidakis, I. Angelozzi, P. Anger, A. Angerami, F. Anghinolfi, A. V. Anisenkov, N. Anjos, A. Annovi, M. Antonelli, A. Antonov, J. Antos, F. Anulli, M. Aoki, L. Aperio Bella, G. Arabidze, Y. Arai, J. P. Araque, A. T. H. Arce, F. A. Arduh, J-F. Arguin, S. Argyropoulos, M. Arik, A. J. Armbruster, O. Arnaez, V. Arnal, H. Arnold, M. Arratia, O. Arslan, A. Artamonov, G. Artoni, S. Asai, N. Asbah, A. Ashkenazi, B. Åsman, L. Asquith, K. Assamagan, R. Astalos, M. Atkinson, N. B. Atlay, B. Auerbach, K. Augsten, M. Aurousseau, G. Avolio, B. Axen, M. K. Ayoub, G. Azuelos, M. A. Baak, A. E. Baas, C. Bacci, H. Bachacou, K. Bachas, M. Backes, M. Backhaus, E. Badescu, P. Bagiacchi, P. Bagnaia, Y. Bai, T. Bain, J. T. Baines, O. K. Baker, P. Balek, T. Balestri, F. Balli, E. Banas, Sw. Banerjee, A. A. E. Bannoura, H. S. Bansil, L. Barak, S. P. Baranov, E. L. Barberio, D. Barberis, M. Barbero, T. Barillari, M. Barisonzi, T. Barklow, N. Barlow, S. L. Barnes, B. M. Barnett, R. M. Barnett, Z. Barnovska, A. Baroncelli, G. Barone, A. J. Barr, F. Barreiro, J. Barreiro Guimarães da Costa, R. Bartoldus, A. E. Barton, P. Bartos, A. Bassalat, A. Basye, R. L. Bates, S. J. Batista, J. R. Batley, M. Battaglia, M. Bauce, F. Bauer, H. S. Bawa, J. B. Beacham, M. D. Beattie, T. Beau, P. H. Beauchemin, R. Beccherle, P. Bechtle, H. P. Beck, K. Becker, M. Becker, S. Becker, M. Beckingham, C. Becot, A. J. Beddall, A. Beddall, V. A. Bednyakov, C. P. Bee, L. J. Beemster, T. A. Beermann, M. Begel, J. K. Behr, C. Belanger-Champagne, P. J. Bell, W. H. Bell, G. Bella, L. Bellagamba, A. Bellerive, M. Bellomo, K. Belotskiy, O. Beltramello, O. Benary, D. Benchekroun, M. Bender, K. Bendtz, N. Benekos, Y. Benhammou, E. Benhar Noccioli, J. A. Benitez Garcia, D. P. Benjamin, J. R. Bensinger, S. Bentvelsen, L. Beresford, M. Beretta, D. Berge, E. Bergeaas Kuutmann, N. Berger, F. Berghaus, J. Beringer, C. Bernard, N. R. Bernard, C. Bernius, F. U. Bernlochner, T. Berry, P. Berta, C. Bertella, G. Bertoli, F. Bertolucci, C. Bertsche, D. Bertsche, M. I. Besana, G. J. Besjes, O. Bessidskaia Bylund, M. Bessner, N. Besson, C. Betancourt, S. Bethke, A. J. Bevan, W. Bhimji, R. M. Bianchi, L. Bianchini, M. Bianco, O. Biebel, S. P. Bieniek, M. Biglietti, J. Bilbao De Mendizabal, H. Bilokon, M. Bindi, S. Binet, A. Bingul, C. Bini, C. W. Black, J. E. Black, K. M. Black, D. Blackburn, R. E. Blair, J.-B. Blanchard, J.E. Blanco, T. Blazek, I. Bloch, C. Blocker, W. Blum, U. Blumenschein, G. J. Bobbink, V. S. Bobrovnikov, S. S. Bocchetta, A. Bocci, C. Bock, M. Boehler, J. A. Bogaerts, A. G. Bogdanchikov, C. Bohm, V. Boisvert, T. Bold, V. Boldea, A. S. Boldyrev, M. Bomben, M. Bona, M. Boonekamp, A. Borisov, G. Borissov, S. Borroni, J. Bortfeldt, V. Bortolotto, K. Bos, D. Boscherini, M. Bosman, J. Boudreau, J. Bouffard, E. V. Bouhova-Thacker, D. Boumediene, C. Bourdarios, N. Bousson, A. Boveia, J. Boyd, I. R. Boyko, I. Bozic, J. Bracinik, A. Brandt, G. Brandt, O. Brandt, U. Bratzler, B. Brau, J. E. Brau, H. M. Braun, S. F. Brazzale, K. Brendlinger, A. J. Brennan, L. Brenner, R. Brenner, S. Bressler, K. Bristow, T. M. Bristow, D. Britton, D. Britzger, F. M. Brochu, I. Brock, R. Brock, J. Bronner, G. Brooijmans, T. Brooks, W. K. Brooks, J. Brosamer, E. Brost, J. Brown, P. A. Bruckman de Renstrom, D. Bruncko, R. Bruneliere, A. Bruni, G. Bruni, M. Bruschi, L. Bryngemark, T. Buanes, Q. Buat, P. Buchholz, A. G. Buckley, S. I. Buda, I. A. Budagov, F. Buehrer, L. Bugge, M. K. Bugge, O. Bulekov, H. Burckhart, S. Burdin, B. Burghgrave, S. Burke, I. Burmeister, E. Busato, D. Büscher, V. Büscher, P. Bussey, C. P. Buszello, J. M. Butler, A. I. Butt, C. M. Buttar, J. M. Butterworth, P. Butti, W. Buttinger, A. Buzatu, R. Buzykaev, S. Cabrera Urbán, D. Caforio, O. Cakir, P. Calafiura, A. Calandri, G. Calderini, P. Calfayan, L. P. Caloba, D. Calvet, S. Calvet, R. Camacho Toro, S. Camarda, D. Cameron, L. M. Caminada, R. Caminal Armadans, S. Campana, M. Campanelli, A. Campoverde, V. Canale, A. Canepa, M. Cano Bret, J. Cantero, R. Cantrill, T. Cao, M. D. M. Capeans Garrido, I. Caprini, M. Caprini, M. Capua, R. Caputo, R. Cardarelli, T. Carli, G. Carlino, L. Carminati, S. Caron, E. Carquin, G. D. Carrillo-Montoya, J. R. Carter, J. Carvalho, D. Casadei, M. P. Casado, M. Casolino, E. Castaneda-Miranda, A. Castelli, V. Castillo Gimenez, N. F. Castro, P. Catastini, A. Catinaccio, J. R. Catmore, A. Cattai, J. Caudron, V. Cavaliere, D. Cavalli, M. Cavalli-Sforza, V. Cavasinni, F. Ceradini, B. C. Cerio, K. Cerny, A. S. Cerqueira, A. Cerri, L. Cerrito, F. Cerutti, M. Cerv, A. Cervelli, S. A. Cetin, A. Chafaq, D. Chakraborty, I. Chalupkova, P. Chang, B. Chapleau, J. D. Chapman, D. G. Charlton, C. C. Chau, C. A. Chavez Barajas, S. Cheatham, A. Chegwidden, S. Chekanov, S. V. Chekulaev, G. A. Chelkov, M. A. Chelstowska, C. Chen, H. Chen, K. Chen, L. Chen, S. Chen, X. Chen, Y. Chen, H. C. Cheng, Y. Cheng, A. Cheplakov, E. Cheremushkina, R. Cherkaoui El Moursli, V. Chernyatin, E. Cheu, L. Chevalier, V. Chiarella, J. T. Childers, G. Chiodini, A. S. Chisholm, R. T. Chislett, A. Chitan, M. V. Chizhov, K. Choi, S. Chouridou, B. K. B. Chow, V. Christodoulou, D. Chromek-Burckhart, M. L. Chu, J. Chudoba, A. J. Chuinard, J. J. Chwastowski, L. Chytka, G. Ciapetti, A. K. Ciftci, D. Cinca, V. Cindro, I. A. Cioara, A. Ciocio, Z. H. Citron, M. Ciubancan, A. Clark, B. L. Clark, P. J. Clark, R. N. Clarke, W. Cleland, C. Clement, Y. Coadou, M. Cobal, A. Coccaro, J. Cochran, L. Coffey, J. G. Cogan, B. Cole, S. Cole, A. P. Colijn, J. Collot, T. Colombo, G. Compostella, P. Conde Muiño, E. Coniavitis, S. H. Connell, I. A. Connelly, S. M. Consonni, V. Consorti, S. Constantinescu, C. Conta, G. Conti, F. Conventi, M. Cooke, B. D. Cooper, A. M. Cooper-Sarkar, K. Copic, T. Cornelissen, M. Corradi, F. Corriveau, A. Corso-Radu, A. Cortes-Gonzalez, G. Cortiana, G. Costa, M. J. Costa, D. Costanzo, D. Côté, G. Cottin, G. Cowan, B. E. Cox, K. Cranmer, G. Cree, S. Crépé-Renaudin, F. Crescioli, W. A. Cribbs, M. Crispin Ortuzar, M. Cristinziani, V. Croft, G. Crosetti, T. Cuhadar Donszelmann, J. Cummings, M. Curatolo, C. Cuthbert, H. Czirr, P. Czodrowski, S. D’Auria, M. D’Onofrio, M. J. Da Cunha Sargedas De Sousa, C. Da Via, W. Dabrowski, A. Dafinca, T. Dai, O. Dale, F. Dallaire, C. Dallapiccola, M. Dam, J. R. Dandoy, A. C. Daniells, M. Danninger, M. Dano Hoffmann, V. Dao, G. Darbo, S. Darmora, J. Dassoulas, A. Dattagupta, W. Davey, C. David, T. Davidek, E. Davies, M. Davies, P. Davison, Y. Davygora, E. Dawe, I. Dawson, R. K. Daya-Ishmukhametova, K. De, R. de Asmundis, S. De Castro, S. De Cecco, N. De Groot, P. de Jong, H. De la Torre, F. De Lorenzi, L. De Nooij, D. De Pedis, A. De Salvo, U. De Sanctis, A. De Santo, J. B. De Vivie De Regie, W. J. Dearnaley, R. Debbe, C. Debenedetti, D. V. Dedovich, I. Deigaard, J. Del Peso, T. Del Prete, D. Delgove, F. Deliot, C. M. Delitzsch, M. Deliyergiyev, A. Dell’Acqua, L. Dell’Asta, M. Dell’Orso, M. Della Pietra, D. della Volpe, M. Delmastro, P. A. Delsart, C. Deluca, D. A. DeMarco, S. Demers, M. Demichev, A. Demilly, S. P. Denisov, D. Derendarz, J. E. Derkaoui, F. Derue, P. Dervan, K. Desch, C. Deterre, P. O. Deviveiros, A. Dewhurst, S. Dhaliwal, A. Di Ciaccio, L. Di Ciaccio, A. Di Domenico, C. Di Donato, A. Di Girolamo, B. Di Girolamo, A. Di Mattia, B. Di Micco, R. Di Nardo, A. Di Simone, R. Di Sipio, D. Di Valentino, C. Diaconu, M. Diamond, F. A. Dias, M. A. Diaz, E. B. Diehl, J. Dietrich, S. Diglio, A. Dimitrievska, J. Dingfelder, F. Dittus, F. Djama, T. Djobava, J. I. Djuvsland, M. A. B. do Vale, D. Dobos, M. Dobre, C. Doglioni, T. Dohmae, J. Dolejsi, Z. Dolezal, B. A. Dolgoshein, M. Donadelli, S. Donati, P. Dondero, J. Donini, J. Dopke, A. Doria, M. T. Dova, A. T. Doyle, E. Drechsler, M. Dris, E. Dubreuil, E. Duchovni, G. Duckeck, O. A. Ducu, D. Duda, A. Dudarev, L. Duflot, L. Duguid, M. Dührssen, M. Dunford, H. Duran Yildiz, M. Düren, A. Durglishvili, D. Duschinger, M. Dyndal, C. Eckardt, K. M. Ecker, W. Edson, N. C. Edwards, W. Ehrenfeld, T. Eifert, G. Eigen, K. Einsweiler, T. Ekelof, M. El Kacimi, M. Ellert, S. Elles, F. Ellinghaus, A. A. Elliot, N. Ellis, J. Elmsheuser, M. Elsing, D. Emeliyanov, Y. Enari, O. C. Endner, M. Endo, R. Engelmann, J. Erdmann, A. Ereditato, G. Ernis, J. Ernst, M. Ernst, S. Errede, E. Ertel, M. Escalier, H. Esch, C. Escobar, B. Esposito, A. I. Etienvre, E. Etzion, H. Evans, A. Ezhilov, L. Fabbri, G. Facini, R. M. Fakhrutdinov, S. Falciano, R. J. Falla, J. Faltova, Y. Fang, M. Fanti, A. Farbin, A. Farilla, T. Farooque, S. Farrell, S. M. Farrington, P. Farthouat, F. Fassi, P. Fassnacht, D. Fassouliotis, M. Faucci Giannelli, A. Favareto, L. Fayard, P. Federic, O. L. Fedin, W. Fedorko, S. Feigl, L. Feligioni, C. Feng, E. J. Feng, H. Feng, A. B. Fenyuk, P. Fernandez Martinez, S. Fernandez Perez, S. Ferrag, J. Ferrando, A. Ferrari, P. Ferrari, R. Ferrari, D. E. Ferreira de Lima, A. Ferrer, D. Ferrere, C. Ferretti, A. Ferretto Parodi, M. Fiascaris, F. Fiedler, A. Filipčič, M. Filipuzzi, F. Filthaut, M. Fincke-Keeler, K. D. Finelli, M. C. N. Fiolhais, L. Fiorini, A. Firan, A. Fischer, C. Fischer, J. Fischer, W. C. Fisher, E. A. Fitzgerald, M. Flechl, I. Fleck, P. Fleischmann, S. Fleischmann, G. T. Fletcher, G. Fletcher, T. Flick, A. Floderus, L. R. Flores Castillo, M. J. Flowerdew, A. Formica, A. Forti, D. Fournier, H. Fox, S. Fracchia, P. Francavilla, M. Franchini, D. Francis, L. Franconi, M. Franklin, M. Fraternali, D. Freeborn, S. T. French, F. Friedrich, D. Froidevaux, J. A. Frost, C. Fukunaga, E. Fullana Torregrosa, B. G. Fulsom, J. Fuster, C. Gabaldon, O. Gabizon, A. Gabrielli, A. Gabrielli, S. Gadatsch, S. Gadomski, G. Gagliardi, P. Gagnon, C. Galea, B. Galhardo, E. J. Gallas, B. J. Gallop, P. Gallus, G. Galster, K. K. Gan, J. Gao, Y. Gao, Y. S. Gao, F. M. Garay Walls, F. Garberson, C. García, J. E. García Navarro, M. Garcia-Sciveres, R. W. Gardner, N. Garelli, V. Garonne, C. Gatti, A. Gaudiello, G. Gaudio, B. Gaur, L. Gauthier, P. Gauzzi, I. L. Gavrilenko, C. Gay, G. Gaycken, E. N. Gazis, P. Ge, Z. Gecse, C. N. P. Gee, D. A. A. Geerts, Ch. Geich-Gimbel, M. P. Geisler, C. Gemme, M. H. Genest, S. Gentile, M. George, S. George, D. Gerbaudo, A. Gershon, H. Ghazlane, B. Giacobbe, S. Giagu, V. Giangiobbe, P. Giannetti, B. Gibbard, S. M. Gibson, M. Gilchriese, T. P. S. Gillam, D. Gillberg, G. Gilles, D. M. Gingrich, N. Giokaris, M. P. Giordani, F. M. Giorgi, F. M. Giorgi, P. F. Giraud, P. Giromini, D. Giugni, C. Giuliani, M. Giulini, B. K. Gjelsten, S. Gkaitatzis, I. Gkialas, E. L. Gkougkousis, L. K. Gladilin, C. Glasman, J. Glatzer, P. C. F. Glaysher, A. Glazov, M. Goblirsch-Kolb, J. R. Goddard, J. Godlewski, S. Goldfarb, T. Golling, D. Golubkov, A. Gomes, R. Gonçalo, J. Goncalves Pinto Firmino Da Costa, L. Gonella, S. González de la Hoz, G. Gonzalez Parra, S. Gonzalez-Sevilla, L. Goossens, P. A. Gorbounov, H. A. Gordon, I. Gorelov, B. Gorini, E. Gorini, A. Gorišek, E. Gornicki, A. T. Goshaw, C. Gössling, M. I. Gostkin, D. Goujdami, A. G. Goussiou, N. Govender, H. M. X. Grabas, L. Graber, I. Grabowska-Bold, P. Grafström, K-J. Grahn, J. Gramling, E. Gramstad, S. Grancagnolo, V. Grassi, V. Gratchev, H. M. Gray, E. Graziani, Z. D. Greenwood, K. Gregersen, I. M. Gregor, P. Grenier, J. Griffiths, A. A. Grillo, K. Grimm, S. Grinstein, Ph. Gris, J.-F. Grivaz, J. P. Grohs, A. Grohsjean, E. Gross, J. Grosse-Knetter, G. C. Grossi, Z. J. Grout, L. Guan, J. Guenther, F. Guescini, D. Guest, O. Gueta, E. Guido, T. Guillemin, S. Guindon, U. Gul, C. Gumpert, J. Guo, S. Gupta, P. Gutierrez, N. G. Gutierrez Ortiz, C. Gutschow, C. Guyot, C. Gwenlan, C. B. Gwilliam, A. Haas, C. Haber, H. K. Hadavand, N. Haddad, P. Haefner, S. Hageböck, Z. Hajduk, H. Hakobyan, M. Haleem, J. Haley, D. Hall, G. Halladjian, G. D. Hallewell, K. Hamacher, P. Hamal, K. Hamano, M. Hamer, A. Hamilton, S. Hamilton, G. N. Hamity, P. G. Hamnett, L. Han, K. Hanagaki, K. Hanawa, M. Hance, P. Hanke, R. Hann, J. B. Hansen, J. D. Hansen, M. C. Hansen, P. H. Hansen, K. Hara, A. S. Hard, T. Harenberg, F. Hariri, S. Harkusha, R. D. Harrington, P. F. Harrison, F. Hartjes, M. Hasegawa, S. Hasegawa, Y. Hasegawa, A. Hasib, S. Hassani, S. Haug, R. Hauser, L. Hauswald, M. Havranek, C. M. Hawkes, R. J. Hawkings, A. D. Hawkins, T. Hayashi, D. Hayden, C. P. Hays, J. M. Hays, H. S. Hayward, S. J. Haywood, S. J. Head, T. Heck, V. Hedberg, L. Heelan, S. Heim, T. Heim, B. Heinemann, L. Heinrich, J. Hejbal, L. Helary, S. Hellman, D. Hellmich, C. Helsens, J. Henderson, R. C. W. Henderson, Y. Heng, C. Hengler, S. Henkelmann, A. Henrichs, A. M. Henriques Correia, S. Henrot-Versille, G. H. Herbert, Y. Hernández Jiménez, R. Herrberg-Schubert, G. Herten, R. Hertenberger, L. Hervas, G. G. Hesketh, N. P. Hessey, J. W. Hetherly, R. Hickling, E. Higón-Rodriguez, E. Hill, J. C. Hill, K. H. Hiller, S. J. Hillier, I. Hinchliffe, E. Hines, R. R. Hinman, M. Hirose, D. Hirschbuehl, J. Hobbs, N. Hod, M. C. Hodgkinson, P. Hodgson, A. Hoecker, M. R. Hoeferkamp, F. Hoenig, M. Hohlfeld, D. Hohn, T. R. Holmes, T. M. Hong, L. Hooft van Huysduynen, W. H. Hopkins, Y. Horii, A. J. Horton, J-Y. Hostachy, S. Hou, A. Hoummada, J. Howard, J. Howarth, M. Hrabovsky, I. Hristova, J. Hrivnac, T. Hryn’ova, A. Hrynevich, C. Hsu, P. J. Hsu, S.-C. Hsu, D. Hu, Q. Hu, X. Hu, Y. Huang, Z. Hubacek, F. Hubaut, F. Huegging, T. B. Huffman, E. W. Hughes, G. Hughes, M. Huhtinen, T. A. Hülsing, N. Huseynov, J. Huston, J. Huth, G. Iacobucci, G. Iakovidis, I. Ibragimov, L. Iconomidou-Fayard, E. Ideal, Z. Idrissi, P. Iengo, O. Igonkina, T. Iizawa, Y. Ikegami, K. Ikematsu, M. Ikeno, Y. Ilchenko, D. Iliadis, N. Ilic, Y. Inamaru, T. Ince, P. Ioannou, M. Iodice, K. Iordanidou, V. Ippolito, A. Irles Quiles, C. Isaksson, M. Ishino, M. Ishitsuka, R. Ishmukhametov, C. Issever, S. Istin, J. M. Iturbe Ponce, R. Iuppa, J. Ivarsson, W. Iwanski, H. Iwasaki, J. M. Izen, V. Izzo, S. Jabbar, B. Jackson, M. Jackson, P. Jackson, M. R. Jaekel, V. Jain, K. Jakobs, S. Jakobsen, T. Jakoubek, J. Jakubek, D. O. Jamin, D. K. Jana, E. Jansen, R. W. Jansky, J. Janssen, M. Janus, G. Jarlskog, N. Javadov, T. Javůrek, L. Jeanty, J. Jejelava, G.-Y. Jeng, D. Jennens, P. Jenni, J. Jentzsch, C. Jeske, S. Jézéquel, H. Ji, J. Jia, Y. Jiang, S. Jiggins, J. Jimenez Pena, S. Jin, A. Jinaru, O. Jinnouchi, M. D. Joergensen, P. Johansson, K. A. Johns, K. Jon-And, G. Jones, R. W. L. Jones, T. J. Jones, J. Jongmanns, P. M. Jorge, K. D. Joshi, J. Jovicevic, X. Ju, C. A. Jung, P. Jussel, A. Juste Rozas, M. Kaci, A. Kaczmarska, M. Kado, H. Kagan, M. Kagan, S. J. Kahn, E. Kajomovitz, C. W. Kalderon, S. Kama, A. Kamenshchikov, N. Kanaya, M. Kaneda, S. Kaneti, V. A. Kantserov, J. Kanzaki, B. Kaplan, A. Kapliy, D. Kar, K. Karakostas, A. Karamaoun, N. Karastathis, M. J. Kareem, M. Karnevskiy, S. N. Karpov, Z. M. Karpova, K. Karthik, V. Kartvelishvili, A. N. Karyukhin, L. Kashif, R. D. Kass, A. Kastanas, Y. Kataoka, A. Katre, J. Katzy, K. Kawagoe, T. Kawamoto, G. Kawamura, S. Kazama, V. F. Kazanin, M. Y. Kazarinov, R. Keeler, R. Kehoe, M. Keil, J. S. Keller, J. J. Kempster, H. Keoshkerian, O. Kepka, B. P. Kerševan, S. Kersten, R. A. Keyes, F. Khalil-zada, H. Khandanyan, A. Khanov, A.G. Kharlamov, T. J. Khoo, G. Khoriauli, V. Khovanskiy, E. Khramov, J. Khubua, H. Y. Kim, H. Kim, S. H. Kim, Y. Kim, N. Kimura, O. M. Kind, B. T. King, M. King, R. S. B. King, S. B. King, J. Kirk, A. E. Kiryunin, T. Kishimoto, D. Kisielewska, F. Kiss, K. Kiuchi, O. Kivernyk, E. Kladiva, M. H. Klein, M. Klein, U. Klein, K. Kleinknecht, P. Klimek, A. Klimentov, R. Klingenberg, J. A. Klinger, T. Klioutchnikova, P. F. Klok, E.-E. Kluge, P. Kluit, S. Kluth, E. Kneringer, E. B. F. G. Knoops, A. Knue, D. Kobayashi, T. Kobayashi, M. Kobel, M. Kocian, P. Kodys, T. Koffas, E. Koffeman, L. A. Kogan, S. Kohlmann, Z. Kohout, T. Kohriki, T. Koi, H. Kolanoski, I. Koletsou, A. A. Komar, Y. Komori, T. Kondo, N. Kondrashova, K. Köneke, A. C. König, S. König, T. Kono, R. Konoplich, N. Konstantinidis, R. Kopeliansky, S. Koperny, L. Köpke, A. K. Kopp, K. Korcyl, K. Kordas, A. Korn, A. A. Korol, I. Korolkov, E. V. Korolkova, O. Kortner, S. Kortner, T. Kosek, V. V. Kostyukhin, V. M. Kotov, A. Kotwal, A. Kourkoumeli-Charalampidi, C. Kourkoumelis, V. Kouskoura, A. Koutsman, R. Kowalewski, T. Z. Kowalski, W. Kozanecki, A. S. Kozhin, V. A. Kramarenko, G. Kramberger, D. Krasnopevtsev, M. W. Krasny, A. Krasznahorkay, J. K. Kraus, A. Kravchenko, S. Kreiss, M. Kretz, J. Kretzschmar, K. Kreutzfeldt, P. Krieger, K. Krizka, K. Kroeninger, H. Kroha, J. Kroll, J. Kroseberg, J. Krstic, U. Kruchonak, H. Krüger, N. Krumnack, Z. V. Krumshteyn, A. Kruse, M. C. Kruse, M. Kruskal, T. Kubota, H. Kucuk, S. Kuday, S. Kuehn, A. Kugel, F. Kuger, A. Kuhl, T. Kuhl, V. Kukhtin, Y. Kulchitsky, S. Kuleshov, M. Kuna, T. Kunigo, A. Kupco, H. Kurashige, Y. A. Kurochkin, R. Kurumida, V. Kus, E. S. Kuwertz, M. Kuze, J. Kvita, T. Kwan, D. Kyriazopoulos, A. La Rosa, J. L. La Rosa Navarro, L. La Rotonda, C. Lacasta, F. Lacava, J. Lacey, H. Lacker, D. Lacour, V. R. Lacuesta, E. Ladygin, R. Lafaye, B. Laforge, T. Lagouri, S. Lai, L. Lambourne, S. Lammers, C. L. Lampen, W. Lampl, E. Lançon, U. Landgraf, M. P. J. Landon, V. S. Lang, J. C. Lange, A. J. Lankford, F. Lanni, K. Lantzsch, S. Laplace, C. Lapoire, J. F. Laporte, T. Lari, F. Lasagni Manghi, M. Lassnig, P. Laurelli, W. Lavrijsen, A. T. Law, P. Laycock, O. Le Dortz, E. Le Guirriec, E. Le Menedeu, M. LeBlanc, T. LeCompte, F. Ledroit-Guillon, C. A. Lee, S. C. Lee, L. Lee, G. Lefebvre, M. Lefebvre, F. Legger, C. Leggett, A. Lehan, G. Lehmann Miotto, X. Lei, W. A. Leight, A. Leisos, A. G. Leister, M. A. L. Leite, R. Leitner, D. Lellouch, B. Lemmer, K. J. C. Leney, T. Lenz, G. Lenzen, B. Lenzi, R. Leone, S. Leone, C. Leonidopoulos, S. Leontsinis, C. Leroy, C. G. Lester, M. Levchenko, J. Levêque, D. Levin, L. J. Levinson, M. Levy, A. Lewis, A. M. Leyko, M. Leyton, B. Li, H. Li, H. L. Li, L. Li, L. Li, S. Li, Y. Li, Z. Liang, H. Liao, B. Liberti, A. Liblong, P. Lichard, K. Lie, J. Liebal, W. Liebig, C. Limbach, A. Limosani, S. C. Lin, T. H. Lin, F. Linde, B. E. Lindquist, J. T. Linnemann, E. Lipeles, A. Lipniacka, M. Lisovyi, T. M. Liss, D. Lissauer, A. Lister, A. M. Litke, B. Liu, D. Liu, J. Liu, J. B. Liu, K. Liu, L. Liu, M. Liu, M. Liu, Y. Liu, M. Livan, A. Lleres, J. Llorente Merino, S. L. Lloyd, F. Lo Sterzo, E. Lobodzinska, P. Loch, W. S. Lockman, F. K. Loebinger, A. E. Loevschall-Jensen, A. Loginov, T. Lohse, K. Lohwasser, M. Lokajicek, B. A. Long, J. D. Long, R. E. Long, K. A. Looper, L. Lopes, D. Lopez Mateos, B. Lopez Paredes, I. Lopez Paz, J. Lorenz, N. Lorenzo Martinez, M. Losada, P. Loscutoff, P. J. Lösel, X. Lou, A. Lounis, J. Love, P. A. Love, N. Lu, H. J. Lubatti, C. Luci, A. Lucotte, F. Luehring, W. Lukas, L. Luminari, O. Lundberg, B. Lund-Jensen, M. Lungwitz, D. Lynn, R. Lysak, E. Lytken, H. Ma, L. L. Ma, G. Maccarrone, A. Macchiolo, C. M. Macdonald, J. Machado Miguens, D. Macina, D. Madaffari, R. Madar, H. J. Maddocks, W. F. Mader, A. Madsen, S. Maeland, T. Maeno, A. Maevskiy, E. Magradze, K. Mahboubi, J. Mahlstedt, C. Maiani, C. Maidantchik, A. A. Maier, T. Maier, A. Maio, S. Majewski, Y. Makida, N. Makovec, B. Malaescu, Pa. Malecki, V. P. Maleev, F. Malek, U. Mallik, D. Malon, C. Malone, S. Maltezos, V. M. Malyshev, S. Malyukov, J. Mamuzic, G. Mancini, B. Mandelli, L. Mandelli, I. Mandić, R. Mandrysch, J. Maneira, A. Manfredini, L. Manhaes de Andrade Filho, J. Manjarres Ramos, A. Mann, P. M. Manning, A. Manousakis-Katsikakis, B. Mansoulie, R. Mantifel, M. Mantoani, L. Mapelli, L. March, G. Marchiori, M. Marcisovsky, C. P. Marino, M. Marjanovic, F. Marroquim, S. P. Marsden, Z. Marshall, L. F. Marti, S. Marti-Garcia, B. Martin, T. A. Martin, V. J. Martin, B. Martin dit Latour, M. Martinez, S. Martin-Haugh, V. S. Martoiu, A. C. Martyniuk, M. Marx, F. Marzano, A. Marzin, L. Masetti, T. Mashimo, R. Mashinistov, J. Masik, A. L. Maslennikov, I. Massa, L. Massa, N. Massol, P. Mastrandrea, A. Mastroberardino, T. Masubuchi, P. Mättig, J. Mattmann, J. Maurer, S. J. Maxfield, D. A. Maximov, R. Mazini, S. M. Mazza, L. Mazzaferro, G. Mc Goldrick, S. P. Mc Kee, A. McCarn, R. L. McCarthy, T. G. McCarthy, N. A. McCubbin, K. W. McFarlane, J. A. Mcfayden, G. Mchedlidze, S. J. McMahon, R. A. McPherson, M. Medinnis, S. Meehan, S. Mehlhase, A. Mehta, K. Meier, C. Meineck, B. Meirose, B. R. Mellado Garcia, F. Meloni, A. Mengarelli, S. Menke, E. Meoni, K. M. Mercurio, S. Mergelmeyer, P. Mermod, L. Merola, C. Meroni, F. S. Merritt, A. Messina, J. Metcalfe, A. S. Mete, C. Meyer, C. Meyer, J-P. Meyer, J. Meyer, R. P. Middleton, S. Miglioranzi, L. Mijović, G. Mikenberg, M. Mikestikova, M. Mikuž, M. Milesi, A. Milic, D. W. Miller, C. Mills, A. Milov, D. A. Milstead, A. A. Minaenko, Y. Minami, I. A. Minashvili, A. I. Mincer, B. Mindur, M. Mineev, Y. Ming, L. M. Mir, T. Mitani, J. Mitrevski, V. A. Mitsou, A. Miucci, P. S. Miyagawa, J. U. Mjörnmark, T. Moa, K. Mochizuki, S. Mohapatra, W. Mohr, S. Molander, R. Moles-Valls, K. Mönig, C. Monini, J. Monk, E. Monnier, J. Montejo Berlingen, F. Monticelli, S. Monzani, R. W. Moore, N. Morange, D. Moreno, M. Moreno Llácer, P. Morettini, M. Morgenstern, M. Morii, M. Morinaga, V. Morisbak, S. Moritz, A. K. Morley, G. Mornacchi, J. D. Morris, S. S. Mortensen, A. Morton, L. Morvaj, H. G. Moser, M. Mosidze, J. Moss, K. Motohashi, R. Mount, E. Mountricha, S. V. Mouraviev, E. J. W. Moyse, S. Muanza, R. D. Mudd, F. Mueller, J. Mueller, K. Mueller, R. S. P. Mueller, T. Mueller, D. Muenstermann, P. Mullen, Y. Munwes, J. A. Murillo Quijada, W. J. Murray, H. Musheghyan, E. Musto, A. G. Myagkov, M. Myska, O. Nackenhorst, J. Nadal, K. Nagai, R. Nagai, Y. Nagai, K. Nagano, A. Nagarkar, Y. Nagasaka, K. Nagata, M. Nagel, E. Nagy, A. M. Nairz, Y. Nakahama, K. Nakamura, T. Nakamura, I. Nakano, H. Namasivayam, G. Nanava, R. F. Naranjo Garcia, R. Narayan, T. Naumann, G. Navarro, R. Nayyar, H. A. Neal, P. Yu. Nechaeva, T. J. Neep, P. D. Nef, A. Negri, M. Negrini, S. Nektarijevic, C. Nellist, A. Nelson, S. Nemecek, P. Nemethy, A. A. Nepomuceno, M. Nessi, M. S. Neubauer, M. Neumann, R. M. Neves, P. Nevski, P. R. Newman, D. H. Nguyen, R. B. Nickerson, R. Nicolaidou, B. Nicquevert, J. Nielsen, N. Nikiforou, A. Nikiforov, V. Nikolaenko, I. Nikolic-Audit, K. Nikolopoulos, J. K. Nilsen, P. Nilsson, Y. Ninomiya, A. Nisati, R. Nisius, T. Nobe, M. Nomachi, I. Nomidis, T. Nooney, S. Norberg, M. Nordberg, O. Novgorodova, S. Nowak, M. Nozaki, L. Nozka, K. Ntekas, G. Nunes Hanninger, T. Nunnemann, E. Nurse, F. Nuti, B. J. O’Brien, F. O’grady, D. C. O’Neil, V. O’Shea, F. G. Oakham, H. Oberlack, T. Obermann, J. Ocariz, A. Ochi, I. Ochoa, S. Oda, S. Odaka, H. Ogren, A. Oh, S. H. Oh, C. C. Ohm, H. Ohman, H. Oide, W. Okamura, H. Okawa, Y. Okumura, T. Okuyama, A. Olariu, S. A. Olivares Pino, D. Oliveira Damazio, E. Oliver Garcia, A. Olszewski, J. Olszowska, A. Onofre, P. U. E. Onyisi, C. J. Oram, M. J. Oreglia, Y. Oren, D. Orestano, N. Orlando, C. Oropeza Barrera, R. S. Orr, B. Osculati, R. Ospanov, G. Otero y Garzon, H. Otono, M. Ouchrif, E. A. Ouellette, F. Ould-Saada, A. Ouraou, K. P. Oussoren, Q. Ouyang, A. Ovcharova, M. Owen, R. E. Owen, V. E. Ozcan, N. Ozturk, K. Pachal, A. Pacheco Pages, C. Padilla Aranda, M. Pagáčová, S. Pagan Griso, E. Paganis, C. Pahl, F. Paige, P. Pais, K. Pajchel, G. Palacino, S. Palestini, M. Palka, D. Pallin, A. Palma, Y. B. Pan, E. Panagiotopoulou, C. E. Pandini, J. G. Panduro Vazquez, P. Pani, S. Panitkin, L. Paolozzi, Th. D. Papadopoulou, K. Papageorgiou, A. Paramonov, D. Paredes Hernandez, M. A. Parker, K. A. Parker, F. Parodi, J. A. Parsons, U. Parzefall, E. Pasqualucci, S. Passaggio, F. Pastore, Fr. Pastore, G. Pásztor, S. Pataraia, N. D. Patel, J. R. Pater, T. Pauly, J. Pearce, B. Pearson, L. E. Pedersen, M. Pedersen, S. Pedraza Lopez, R. Pedro, S. V. Peleganchuk, D. Pelikan, H. Peng, B. Penning, J. Penwell, D. V. Perepelitsa, E. Perez Codina, M. T. Pérez García-Estañ, L. Perini, H. Pernegger, S. Perrella, R. Peschke, V. D. Peshekhonov, K. Peters, R. F. Y. Peters, B. A. Petersen, T. C. Petersen, E. Petit, A. Petridis, C. Petridou, E. Petrolo, F. Petrucci, N. E. Pettersson, R. Pezoa, P. W. Phillips, G. Piacquadio, E. Pianori, A. Picazio, E. Piccaro, M. Piccinini, M. A. Pickering, R. Piegaia, D. T. Pignotti, J. E. Pilcher, A. D. Pilkington, J. Pina, M. Pinamonti, J. L. Pinfold, A. Pingel, B. Pinto, S. Pires, M. Pitt, C. Pizio, L. Plazak, M.-A. Pleier, V. Pleskot, E. Plotnikova, P. Plucinski, D. Pluth, R. Poettgen, L. Poggioli, D. Pohl, G. Polesello, A. Policicchio, R. Polifka, A. Polini, C. S. Pollard, V. Polychronakos, K. Pommès, L. Pontecorvo, B. G. Pope, G. A. Popeneciu, D. S. Popovic, A. Poppleton, S. Pospisil, K. Potamianos, I. N. Potrap, C. J. Potter, C. T. Potter, G. Poulard, J. Poveda, V. Pozdnyakov, P. Pralavorio, A. Pranko, S. Prasad, S. Prell, D. Price, L. E. Price, M. Primavera, S. Prince, M. Proissl, K. Prokofiev, F. Prokoshin, E. Protopapadaki, S. Protopopescu, J. Proudfoot, M. Przybycien, E. Ptacek, D. Puddu, E. Pueschel, D. Puldon, M. Purohit, P. Puzo, J. Qian, G. Qin, Y. Qin, A. Quadt, D. R. Quarrie, W. B. Quayle, M. Queitsch-Maitland, D. Quilty, S. Raddum, V. Radeka, V. Radescu, S. K. Radhakrishnan, P. Radloff, P. Rados, F. Ragusa, G. Rahal, S. Rajagopalan, M. Rammensee, C. Rangel-Smith, F. Rauscher, S. Rave, T. Ravenscroft, M. Raymond, A. L. Read, N. P. Readioff, D. M. Rebuzzi, A. Redelbach, G. Redlinger, R. Reece, K. Reeves, L. Rehnisch, H. Reisin, M. Relich, C. Rembser, H. Ren, A. Renaud, M. Rescigno, S. Resconi, O. L. Rezanova, P. Reznicek, R. Rezvani, R. Richter, S. Richter, E. Richter-Was, O. Ricken, M. Ridel, P. Rieck, C. J. Riegel, J. Rieger, M. Rijssenbeek, A. Rimoldi, L. Rinaldi, B. Ristić, E. Ritsch, I. Riu, F. Rizatdinova, E. Rizvi, S. H. Robertson, A. Robichaud-Veronneau, D. Robinson, J. E. M. Robinson, A. Robson, C. Roda, S. Roe, O. Røhne, S. Rolli, A. Romaniouk, M. Romano, S. M. Romano Saez, E. Romero Adam, N. Rompotis, M. Ronzani, L. Roos, E. Ros, S. Rosati, K. Rosbach, P. Rose, P. L. Rosendahl, O. Rosenthal, V. Rossetti, E. Rossi, L. P. Rossi, R. Rosten, M. Rotaru, I. Roth, J. Rothberg, D. Rousseau, C. R. Royon, A. Rozanov, Y. Rozen, X. Ruan, F. Rubbo, I. Rubinskiy, V. I. Rud, C. Rudolph, M. S. Rudolph, F. Rühr, A. Ruiz-Martinez, Z. Rurikova, N. A. Rusakovich, A. Ruschke, H. L. Russell, J. P. Rutherfoord, N. Ruthmann, Y. F. Ryabov, M. Rybar, G. Rybkin, N. C. Ryder, A. F. Saavedra, G. Sabato, S. Sacerdoti, A. Saddique, H. F-W. Sadrozinski, R. Sadykov, F. Safai Tehrani, M. Saimpert, H. Sakamoto, Y. Sakurai, G. Salamanna, A. Salamon, M. Saleem, D. Salek, P. H. Sales De Bruin, D. Salihagic, A. Salnikov, J. Salt, D. Salvatore, F. Salvatore, A. Salvucci, A. Salzburger, D. Sampsonidis, A. Sanchez, J. Sánchez, V. Sanchez Martinez, H. Sandaker, R. L. Sandbach, H. G. Sander, M. P. Sanders, M. Sandhoff, C. Sandoval, R. Sandstroem, D. P. C. Sankey, M. Sannino, A. Sansoni, C. Santoni, R. Santonico, H. Santos, I. Santoyo Castillo, K. Sapp, A. Sapronov, J. G. Saraiva, B. Sarrazin, O. Sasaki, Y. Sasaki, K. Sato, G. Sauvage, E. Sauvan, G. Savage, P. Savard, C. Sawyer, L. Sawyer, J. Saxon, C. Sbarra, A. Sbrizzi, T. Scanlon, D. A. Scannicchio, M. Scarcella, V. Scarfone, J. Schaarschmidt, P. Schacht, D. Schaefer, R. Schaefer, J. Schaeffer, S. Schaepe, S. Schaetzel, U. Schäfer, A. C. Schaffer, D. Schaile, R. D. Schamberger, V. Scharf, V. A. Schegelsky, D. Scheirich, M. Schernau, C. Schiavi, C. Schillo, M. Schioppa, S. Schlenker, E. Schmidt, K. Schmieden, C. Schmitt, S. Schmitt, S. Schmitt, B. Schneider, Y. J. Schnellbach, U. Schnoor, L. Schoeffel, A. Schoening, B. D. Schoenrock, E. Schopf, A. L. S. Schorlemmer, M. Schott, D. Schouten, J. Schovancova, S. Schramm, M. Schreyer, C. Schroeder, N. Schuh, M. J. Schultens, H.-C. Schultz-Coulon, H. Schulz, M. Schumacher, B. A. Schumm, Ph. Schune, C. Schwanenberger, A. Schwartzman, T. A. Schwarz, Ph. Schwegler, Ph. Schwemling, R. Schwienhorst, J. Schwindling, T. Schwindt, M. Schwoerer, F. G. Sciacca, E. Scifo, G. Sciolla, F. Scuri, F. Scutti, J. Searcy, G. Sedov, E. Sedykh, P. Seema, S. C. Seidel, A. Seiden, F. Seifert, J. M. Seixas, G. Sekhniaidze, S. J. Sekula, K. E. Selbach, D. M. Seliverstov, N. Semprini-Cesari, C. Serfon, L. Serin, L. Serkin, T. Serre, R. Seuster, H. Severini, T. Sfiligoj, F. Sforza, A. Sfyrla, E. Shabalina, M. Shamim, L. Y. Shan, R. Shang, J. T. Shank, M. Shapiro, P. B. Shatalov, K. Shaw, A. Shcherbakova, C. Y. Shehu, P. Sherwood, L. Shi, S. Shimizu, C. O. Shimmin, M. Shimojima, M. Shiyakova, A. Shmeleva, D. Shoaleh Saadi, M. J. Shochet, S. Shojaii, S. Shrestha, E. Shulga, M. A. Shupe, S. Shushkevich, P. Sicho, O. Sidiropoulou, D. Sidorov, A. Sidoti, F. Siegert, Dj. Sijacki, J. Silva, Y. Silver, S. B. Silverstein, V. Simak, O. Simard, Lj. Simic, S. Simion, E. Simioni, B. Simmons, D. Simon, R. Simoniello, P. Sinervo, N. B. Sinev, G. Siragusa, A. N. Sisakyan, S. Yu. Sivoklokov, J. Sjölin, T. B. Sjursen, M. B. Skinner, H. P. Skottowe, P. Skubic, M. Slater, T. Slavicek, M. Slawinska, K. Sliwa, V. Smakhtin, B. H. Smart, L. Smestad, S. Yu. Smirnov, Y. Smirnov, L. N. Smirnova, O. Smirnova, M. N. K. Smith, M. Smizanska, K. Smolek, A. A. Snesarev, G. Snidero, S. Snyder, R. Sobie, F. Socher, A. Soffer, D. A. Soh, C. A. Solans, M. Solar, J. Solc, E. Yu. Soldatov, U. Soldevila, A. A. Solodkov, A. Soloshenko, O. V. Solovyanov, V. Solovyev, P. Sommer, H. Y. Song, N. Soni, A. Sood, A. Sopczak, B. Sopko, V. Sopko, V. Sorin, D. Sosa, M. Sosebee, C. L. Sotiropoulou, R. Soualah, P. Soueid, A. M. Soukharev, D. South, S. Spagnolo, M. Spalla, F. Spanò, W. R. Spearman, F. Spettel, R. Spighi, G. Spigo, L. A. Spiller, M. Spousta, T. Spreitzer, R. D. St. Denis, S. Staerz, J. Stahlman, R. Stamen, S. Stamm, E. Stanecka, C. Stanescu, M. Stanescu-Bellu, M. M. Stanitzki, S. Stapnes, E. A. Starchenko, J. Stark, P. Staroba, P. Starovoitov, R. Staszewski, P. Stavina, P. Steinberg, B. Stelzer, H. J. Stelzer, O. Stelzer-Chilton, H. Stenzel, S. Stern, G. A. Stewart, J. A. Stillings, M. C. Stockton, M. Stoebe, G. Stoicea, P. Stolte, S. Stonjek, A. R. Stradling, A. Straessner, M. E. Stramaglia, J. Strandberg, S. Strandberg, A. Strandlie, E. Strauss, M. Strauss, P. Strizenec, R. Ströhmer, D. M. Strom, R. Stroynowski, A. Strubig, S. A. Stucci, B. Stugu, N. A. Styles, D. Su, J. Su, R. Subramaniam, A. Succurro, Y. Sugaya, C. Suhr, M. Suk, V. V. Sulin, S. Sultansoy, T. Sumida, S. Sun, X. Sun, J. E. Sundermann, K. Suruliz, G. Susinno, M. R. Sutton, S. Suzuki, Y. Suzuki, M. Svatos, S. Swedish, M. Swiatlowski, I. Sykora, T. Sykora, D. Ta, C. Taccini, K. Tackmann, J. Taenzer, A. Taffard, R. Tafirout, N. Taiblum, H. Takai, R. Takashima, H. Takeda, T. Takeshita, Y. Takubo, M. Talby, A. A. Talyshev, J. Y. C. Tam, K. G. Tan, J. Tanaka, R. Tanaka, S. Tanaka, S. Tanaka, B. B. Tannenwald, N. Tannoury, S. Tapprogge, S. Tarem, F. Tarrade, G. F. Tartarelli, P. Tas, M. Tasevsky, T. Tashiro, E. Tassi, A. Tavares Delgado, Y. Tayalati, F. E. Taylor, G. N. Taylor, W. Taylor, F. A. Teischinger, M. Teixeira Dias Castanheira, P. Teixeira-Dias, K. K. Temming, H. Ten Kate, P. K. Teng, J. J. Teoh, F. Tepel, S. Terada, K. Terashi, J. Terron, S. Terzo, M. Testa, R. J. Teuscher, J. Therhaag, T. Theveneaux-Pelzer, J. P. Thomas, J. Thomas-Wilsker, E. N. Thompson, P. D. Thompson, R. J. Thompson, A. S. Thompson, L. A. Thomsen, E. Thomson, M. Thomson, R. P. Thun, M. J. Tibbetts, R. E. Ticse Torres, V. O. Tikhomirov, Yu. A. Tikhonov, S. Timoshenko, E. Tiouchichine, P. Tipton, S. Tisserant, T. Todorov, S. Todorova-Nova, J. Tojo, S. Tokár, K. Tokushuku, K. Tollefson, E. Tolley, L. Tomlinson, M. Tomoto, L. Tompkins, K. Toms, E. Torrence, H. Torres, E. Torró Pastor, J. Toth, F. Touchard, D. R. Tovey, T. Trefzger, L. Tremblet, A. Tricoli, I. M. Trigger, S. Trincaz-Duvoid, M. F. Tripiana, W. Trischuk, B. Trocmé, C. Troncon, M. Trottier-McDonald, M. Trovatelli, P. True, M. Trzebinski, A. Trzupek, C. Tsarouchas, J. C-L. Tseng, P. V. Tsiareshka, D. Tsionou, G. Tsipolitis, N. Tsirintanis, S. Tsiskaridze, V. Tsiskaridze, E. G. Tskhadadze, I. I. Tsukerman, V. Tsulaia, S. Tsuno, D. Tsybychev, A. Tudorache, V. Tudorache, A. N. Tuna, S. A. Tupputi, S. Turchikhin, D. Turecek, R. Turra, A. J. Turvey, P. M. Tuts, A. Tykhonov, M. Tylmad, M. Tyndel, I. Ueda, R. Ueno, M. Ughetto, M. Ugland, M. Uhlenbrock, F. Ukegawa, G. Unal, A. Undrus, G. Unel, F. C. Ungaro, Y. Unno, C. Unverdorben, J. Urban, P. Urquijo, P. Urrejola, G. Usai, A. Usanova, L. Vacavant, V. Vacek, B. Vachon, C. Valderanis, N. Valencic, S. Valentinetti, A. Valero, L. Valery, S. Valkar, E. Valladolid Gallego, S. Vallecorsa, J. A. Valls Ferrer, W. Van Den Wollenberg, P. C. Van Der Deijl, R. van der Geer, H. van der Graaf, R. Van Der Leeuw, N. van Eldik, P. van Gemmeren, J. Van Nieuwkoop, I. van Vulpen, M. C. van Woerden, M. Vanadia, W. Vandelli, R. Vanguri, A. Vaniachine, F. Vannucci, G. Vardanyan, R. Vari, E. W. Varnes, T. Varol, D. Varouchas, A. Vartapetian, K. E. Varvell, F. Vazeille, T. Vazquez Schroeder, J. Veatch, F. Veloso, T. Velz, S. Veneziano, A. Ventura, D. Ventura, M. Venturi, N. Venturi, A. Venturini, V. Vercesi, M. Verducci, W. Verkerke, J. C. Vermeulen, A. Vest, M. C. Vetterli, O. Viazlo, I. Vichou, T. Vickey, O. E. Vickey Boeriu, G. H. A. Viehhauser, S. Viel, R. Vigne, M. Villa, M. Villaplana Perez, E. Vilucchi, M. G. Vincter, V. B. Vinogradov, I. Vivarelli, F. Vives Vaque, S. Vlachos, D. Vladoiu, M. Vlasak, M. Vogel, P. Vokac, G. Volpi, M. Volpi, H. von der Schmitt, H. von Radziewski, E. von Toerne, V. Vorobel, K. Vorobev, M. Vos, R. Voss, J. H. Vossebeld, N. Vranjes, M. Vranjes Milosavljevic, V. Vrba, M. Vreeswijk, R. Vuillermet, I. Vukotic, Z. Vykydal, P. Wagner, W. Wagner, H. Wahlberg, S. Wahrmund, J. Wakabayashi, J. Walder, R. Walker, W. Walkowiak, C. Wang, F. Wang, H. Wang, H. Wang, J. Wang, J. Wang, K. Wang, R. Wang, S. M. Wang, T. Wang, X. Wang, C. Wanotayaroj, A. Warburton, C. P. Ward, D. R. Wardrope, M. Warsinsky, A. Washbrook, C. Wasicki, P. M. Watkins, A. T. Watson, I. J. Watson, M. F. Watson, G. Watts, S. Watts, B. M. Waugh, S. Webb, M. S. Weber, S. W. Weber, J. S. Webster, A. R. Weidberg, B. Weinert, J. Weingarten, C. Weiser, H. Weits, P. S. Wells, T. Wenaus, T. Wengler, S. Wenig, N. Wermes, M. Werner, P. Werner, M. Wessels, J. Wetter, K. Whalen, A. M. Wharton, A. White, M. J. White, R. White, S. White, D. Whiteson, F. J. Wickens, W. Wiedenmann, M. Wielers, P. Wienemann, C. Wiglesworth, L. A. M. Wiik-Fuchs, A. Wildauer, H. G. Wilkens, H. H. Williams, S. Williams, C. Willis, S. Willocq, A. Wilson, J. A. Wilson, I. Wingerter-Seez, F. Winklmeier, B. T. Winter, M. Wittgen, J. Wittkowski, S. J. Wollstadt, M. W. Wolter, H. Wolters, B. K. Wosiek, J. Wotschack, M. J. Woudstra, K. W. Wozniak, M. Wu, M. Wu, S. L. Wu, X. Wu, Y. Wu, T. R. Wyatt, B. M. Wynne, S. Xella, D. Xu, L. Xu, B. Yabsley, S. Yacoob, R. Yakabe, M. Yamada, Y. Yamaguchi, A. Yamamoto, S. Yamamoto, T. Yamanaka, K. Yamauchi, Y. Yamazaki, Z. Yan, H. Yang, H. Yang, Y. Yang, L. Yao, W-M. Yao, Y. Yasu, E. Yatsenko, K. H. Yau Wong, J. Ye, S. Ye, I. Yeletskikh, A. L. Yen, E. Yildirim, K. Yorita, R. Yoshida, K. Yoshihara, C. Young, C. J. S. Young, S. Youssef, D. R. Yu, J. Yu, J. M. Yu, J. Yu, L. Yuan, A. Yurkewicz, I. Yusuff, B. Zabinski, R. Zaidan, A. M. Zaitsev, J. Zalieckas, A. Zaman, S. Zambito, L. Zanello, D. Zanzi, C. Zeitnitz, M. Zeman, A. Zemla, K. Zengel, O. Zenin, T. Ženiš, D. Zerwas, D. Zhang, F. Zhang, J. Zhang, L. Zhang, R. Zhang, X. Zhang, Z. Zhang, X. Zhao, Y. Zhao, Z. Zhao, A. Zhemchugov, J. Zhong, B. Zhou, C. Zhou, L. Zhou, L. Zhou, N. Zhou, C. G. Zhu, H. Zhu, J. Zhu, Y. Zhu, X. Zhuang, K. Zhukov, A. Zibell, D. Zieminska, N. I. Zimine, C. Zimmermann, R. Zimmermann, S. Zimmermann, Z. Zinonos, M. Zinser, M. Ziolkowski, L. Živković, G. Zobernig, A. Zoccoli, M. zur Nedden, G. Zurzolo, L. Zwalinski, ATLAS Publications

**Affiliations:** Department of Physics, University of Adelaide, Adelaide, Australia; Physics Department, SUNY Albany, Albany, NY USA; Department of Physics, University of Alberta, Edmonton, AB Canada; Department of Physics, Ankara University, Ankara, Turkey; LAPP, CNRS/IN2P3 and Université Savoie Mont Blanc, Annecy-le-Vieux, France; High Energy Physics Division, Argonne National Laboratory, Argonne, IL USA; Department of Physics, University of Arizona, Tucson, AZ USA; Department of Physics, The University of Texas at Arlington, Arlington, TX USA; Physics Department, University of Athens, Athens, Greece; Physics Department, National Technical University of Athens, Zografou, Greece; Institute of Physics, Azerbaijan Academy of Sciences, Baku, Azerbaijan; Institut de Física d’Altes Energies and Departament de Física de la Universitat Autònoma de Barcelona, Barcelona, Spain; Institute of Physics, University of Belgrade, Belgrade, Serbia; Department for Physics and Technology, University of Bergen, Bergen, Norway; Physics Division, Lawrence Berkeley National Laboratory and University of California, Berkeley, CA USA; Department of Physics, Humboldt University, Berlin, Germany; Albert Einstein Center for Fundamental Physics and Laboratory for High Energy Physics, University of Bern, Bern, Switzerland; School of Physics and Astronomy, University of Birmingham, Birmingham, UK; Department of Physics, Bogazici University, Istanbul, Turkey; INFN Sezione di Bologna, Bologna, Italy; Physikalisches Institut, University of Bonn, Bonn, Germany; Department of Physics, Boston University, Boston, MA USA; Department of Physics, Brandeis University, Waltham, MA USA; Universidade Federal do Rio De Janeiro COPPE/EE/IF, Rio de Janeiro, Brazil; Physics Department, Brookhaven National Laboratory, Upton, NY USA; National Institute of Physics and Nuclear Engineering, Bucharest, Romania; Departamento de Física, Universidad de Buenos Aires, Buenos Aires, Argentina; Cavendish Laboratory, University of Cambridge, Cambridge, UK; Department of Physics, Carleton University, Ottawa, ON Canada; CERN, Geneva, Switzerland; Enrico Fermi Institute, University of Chicago, Chicago, IL USA; Departamento de Física, Pontificia Universidad Católica de Chile, Santiago, Chile; Institute of High Energy Physics, Chinese Academy of Sciences, Beijing, China; Laboratoire de Physique Corpusculaire, Clermont Université and Université Blaise Pascal and CNRS/IN2P3, Clermont-Ferrand, France; Nevis Laboratory, Columbia University, Irvington, NY USA; Niels Bohr Institute, University of Copenhagen, Copenhagen, Denmark; INFN Gruppo Collegato di Cosenza, Laboratori Nazionali di Frascati, Frascati, Italy; Faculty of Physics and Applied Computer Science, AGH University of Science and Technology, Kraków, Poland; Institute of Nuclear Physics, Polish Academy of Sciences, Kraków, Poland; Physics Department, Southern Methodist University, Dallas, TX USA; Physics Department, University of Texas at Dallas, Richardson, TX USA; DESY, Hamburg and Zeuthen, Germany; Institut für Experimentelle Physik IV, Technische Universität Dortmund, Dortmund, Germany; Institut für Kern- und Teilchenphysik, Technische Universität Dresden, Dresden, Germany; Department of Physics, Duke University, Durham, NC USA; SUPA - School of Physics and Astronomy, University of Edinburgh, Edinburgh, UK; INFN Laboratori Nazionali di Frascati, Frascati, Italy; Fakultät für Mathematik und Physik, Albert-Ludwigs-Universität, Freiburg, Germany; Section de Physique, Université de Genève, Geneva, Switzerland; INFN Sezione di Genova, Genova, Italy; E. Andronikashvili Institute of Physics, Iv. Javakhishvili Tbilisi State University, Tbilisi, Georgia; II Physikalisches Institut, Justus-Liebig-Universität Giessen, Giessen, Germany; SUPA - School of Physics and Astronomy, University of Glasgow, Glasgow, UK; II Physikalisches Institut, Georg-August-Universität, Göttingen, Germany; Laboratoire de Physique Subatomique et de Cosmologie, Université Grenoble-Alpes, CNRS/IN2P3, Grenoble, France; Department of Physics, Hampton University, Hampton, VA USA; Laboratory for Particle Physics and Cosmology, Harvard University, Cambridge, MA USA; Kirchhoff-Institut für Physik, Ruprecht-Karls-Universität Heidelberg, Heidelberg, Germany; Faculty of Applied Information Science, Hiroshima Institute of Technology, Hiroshima, Japan; Department of Physics, The Chinese University of Hong Kong, Shatin, NT Hong Kong; Department of Physics, Indiana University, Bloomington, IN USA; Institut für Astro- und Teilchenphysik, Leopold-Franzens-Universität, Innsbruck, Austria; University of Iowa, Iowa City, IA USA; Department of Physics and Astronomy, Iowa State University, Ames, IA USA; Joint Institute for Nuclear Research, JINR Dubna, Dubna, Russia; KEK, High Energy Accelerator Research Organization, Tsukuba, Japan; Graduate School of Science, Kobe University, Kobe, Japan; Faculty of Science, Kyoto University, Kyoto, Japan; Kyoto University of Education, Kyoto, Japan; Department of Physics, Kyushu University, Fukuoka, Japan; Instituto de Física La Plata, Universidad Nacional de La Plata and CONICET, La Plata, Argentina; Physics Department, Lancaster University, Lancaster, UK; INFN Sezione di Lecce, Lecce, Italy; Oliver Lodge Laboratory, University of Liverpool, Liverpool, UK; Department of Physics, Jožef Stefan Institute and University of Ljubljana, Ljubljana, Slovenia; School of Physics and Astronomy, Queen Mary University of London, London, UK; Department of Physics, Royal Holloway University of London, Surrey, UK; Department of Physics and Astronomy, University College London, London, UK; Louisiana Tech University, Ruston, LA USA; Laboratoire de Physique Nucléaire et de Hautes Energies, UPMC and Université Paris-Diderot and CNRS/IN2P3, Paris, France; Fysiska institutionen, Lunds universitet, Lund, Sweden; Departamento de Fisica Teorica C-15, Universidad Autonoma de Madrid, Madrid, Spain; Institut für Physik, Universität Mainz, Mainz, Germany; School of Physics and Astronomy, University of Manchester, Manchester, UK; CPPM, Aix-Marseille Université and CNRS/IN2P3, Marseille, France; Department of Physics, University of Massachusetts, Amherst, MA USA; Department of Physics, McGill University, Montreal, QC Canada; School of Physics, University of Melbourne, Melbourne, VIC Australia; Department of Physics, The University of Michigan, Ann Arbor, MI USA; Department of Physics and Astronomy, Michigan State University, East Lansing, MI USA; INFN Sezione di Milano, Milan, Italy; B.I. Stepanov Institute of Physics, National Academy of Sciences of Belarus, Minsk, Republic of Belarus; National Scientific and Educational Centre for Particle and High Energy Physics, Minsk, Republic of Belarus; Department of Physics, Massachusetts Institute of Technology, Cambridge, MA USA; Group of Particle Physics, University of Montreal, Montreal, QC Canada; P.N. Lebedev Institute of Physics, Academy of Sciences, Moscow, Russia; Institute for Theoretical and Experimental Physics (ITEP), Moscow, Russia; National Research Nuclear University MEPhI, Moscow, Russia; D.V. Skobeltsyn Institute of Nuclear Physics, M.V. Lomonosov Moscow State University, Moscow, Russia; Fakultät für Physik, Ludwig-Maximilians-Universität München, Munich, Germany; Max-Planck-Institut für Physik (Werner-Heisenberg-Institut), Munich, Germany; Nagasaki Institute of Applied Science, Nagasaki, Japan; Graduate School of Science and Kobayashi-Maskawa Institute, Nagoya University, Nagoya, Japan; INFN Sezione di Napoli, Naples, Italy; Department of Physics and Astronomy, University of New Mexico, Albuquerque, NM USA; Institute for Mathematics, Astrophysics and Particle Physics, Radboud University Nijmegen/Nikhef, Nijmegen, The Netherlands; Nikhef National Institute for Subatomic Physics and University of Amsterdam, Amsterdam, The Netherlands; Department of Physics, Northern Illinois University, De Kalb, IL USA; Budker Institute of Nuclear Physics, SB RAS, Novosibirsk, Russia; Department of Physics, New York University, New York, NY USA; Ohio State University, Columbus, OH USA; Faculty of Science, Okayama University, Okayama, Japan; Homer L. Dodge Department of Physics and Astronomy, University of Oklahoma, Norman, OK USA; Department of Physics, Oklahoma State University, Stillwater, OK USA; Palacký University, RCPTM, Olomouc, Czech Republic; Center for High Energy Physics, University of Oregon, Eugene, OR USA; LAL, Université Paris-Sud and CNRS/IN2P3, Orsay, France; Graduate School of Science, Osaka University, Osaka, Japan; Department of Physics, University of Oslo, Oslo, Norway; Department of Physics, Oxford University, Oxford, UK; INFN Sezione di Pavia, Pavia, Italy; Department of Physics, University of Pennsylvania, Philadelphia, PA USA; Petersburg Nuclear Physics Institute, Gatchina, Russia; INFN Sezione di Pisa, Pisa, Italy; Department of Physics and Astronomy, University of Pittsburgh, Pittsburgh, PA USA; Laboratorio de Instrumentacao e Fisica Experimental de Particulas, LIP, Lisbon, Portugal; Institute of Physics, Academy of Sciences of the Czech Republic, Prague, Czech Republic; Czech Technical University in Prague, Prague, Czech Republic; Faculty of Mathematics and Physics, Charles University in Prague, Prague, Czech Republic; State Research Center Institute for High Energy Physics, Protvino, Russia; Particle Physics Department, Rutherford Appleton Laboratory, Didcot, UK; Ritsumeikan University, Kusatsu, Shiga Japan; INFN Sezione di Roma, Rome, Italy; INFN Sezione di Roma Tor Vergata, Rome, Italy; INFN Sezione di Roma Tre, Rome, Italy; Faculté des Sciences Ain Chock, Réseau Universitaire de Physique des Hautes Energies-Université Hassan II, Casablanca, Morocco; DSM/IRFU (Institut de Recherches sur les Lois Fondamentales de l’Univers), CEA Saclay (Commissariat à l’Energie Atomique et aux Energies Alternatives), Gif-sur-Yvette, France; Santa Cruz Institute for Particle Physics, University of California Santa Cruz, Santa Cruz, CA USA; Department of Physics, University of Washington, Seattle, WA USA; Department of Physics and Astronomy, University of Sheffield, Sheffield, UK; Department of Physics, Shinshu University, Nagano, Japan; Fachbereich Physik, Universität Siegen, Siegen, Germany; Department of Physics, Simon Fraser University, Burnaby, BC Canada; SLAC National Accelerator Laboratory, Stanford, CA USA; Faculty of Mathematics, Physics and Informatics, Comenius University, Bratislava, Slovak Republic; Department of Physics, University of Cape Town, Cape Town, South Africa; Department of Physics, Stockholm University, Stockholm, Sweden; Physics Department, Royal Institute of Technology, Stockholm, Sweden; Departments of Physics and Astronomy and Chemistry, Stony Brook University, Stony Brook, NY USA; Department of Physics and Astronomy, University of Sussex, Brighton, UK; School of Physics, University of Sydney, Sydney, Australia; Institute of Physics, Academia Sinica, Taipei, Taiwan; Department of Physics, Technion: Israel Institute of Technology, Haifa, Israel; Raymond and Beverly Sackler School of Physics and Astronomy, Tel Aviv University, Tel Aviv, Israel; Department of Physics, Aristotle University of Thessaloniki, Thessaloníki, Greece; International Center for Elementary Particle Physics and Department of Physics, The University of Tokyo, Tokyo, Japan; Graduate School of Science and Technology, Tokyo Metropolitan University, Tokyo, Japan; Department of Physics, Tokyo Institute of Technology, Tokyo, Japan; Department of Physics, University of Toronto, Toronto, ON Canada; TRIUMF, Vancouver, BC Canada; Faculty of Pure and Applied Sciences, University of Tsukuba, Tsukuba, Japan; Department of Physics and Astronomy, Tufts University, Medford, MA USA; Centro de Investigaciones, Universidad Antonio Narino, Bogotá, Colombia; Department of Physics and Astronomy, University of California Irvine, Irvine, CA USA; INFN Gruppo Collegato di Udine, Sezione di Trieste, Udine, Italy; Department of Physics, University of Illinois, Urbana, IL USA; Department of Physics and Astronomy, University of Uppsala, Uppsala, Sweden; Instituto de Física Corpuscular (IFIC) and Departamento de Física Atómica, Molecular y Nuclear and Departamento de Ingeniería Electrónica and Instituto de Microelectrónica de Barcelona (IMB-CNM), University of Valencia and CSIC, Valencia, Spain; Department of Physics, University of British Columbia, Vancouver, BC Canada; Department of Physics and Astronomy, University of Victoria, Victoria, BC Canada; Department of Physics, University of Warwick, Coventry, UK; Waseda University, Tokyo, Japan; Department of Particle Physics, The Weizmann Institute of Science, Rehovot, Israel; Department of Physics, University of Wisconsin, Madison, WI USA; Fakultät für Physik und Astronomie, Julius-Maximilians-Universität, Würzburg, Germany; Fachbereich C Physik, Bergische Universität Wuppertal, Wuppertal, Germany; Department of Physics, Yale University, New Haven, CT USA; Yerevan Physics Institute, Yerevan, Armenia; Centre de Calcul de l’Institut National de Physique Nucléaire et de Physique des Particules (IN2P3), Villeurbanne, France; CERN, Geneva, Switzerland; Istanbul Aydin University, Istanbul, Turkey; Division of Physics, TOBB University of Economics and Technology, Ankara, Turkey; Department of Physics, Dogus University, Istanbul, Turkey; Department of Physics Engineering, Gaziantep University, Gaziantep, Turkey; Dipartimento di Fisica e Astronomia, Università di Bologna, Bologna, Italy; Electrical Circuits Department, Federal University of Juiz de Fora (UFJF), Juiz de Fora, Brazil; Federal University of Sao Joao del Rei (UFSJ), Sao Joao del Rei, Brazil; Instituto de Fisica, Universidade de Sao Paulo, São Paulo, Brazil; National Institute for Research and Development of Isotopic and Molecular Technologies, Physics Department, Cluj Napoca, Romania; University Politehnica Bucharest, Bucharest, Romania; West University in Timisoara, Timisoara, Romania; Departamento de Física, Universidad Técnica Federico Santa María, Valparaiso, Chile; Department of Modern Physics, University of Science and Technology of China, Anhui, China; Department of Physics, Nanjing University, Jiangsu, China; School of Physics, Shandong University, Shandong, China; Department of Physics and Astronomy, Shanghai Key Laboratory for Particle Physics and Cosmology, Shanghai Jiao Tong University, Shanghai, China; Physics Department, Tsinghua University, Beijing, 100084 China; Dipartimento di Fisica, Università della Calabria, Rende, Italy; Marian Smoluchowski Institute of Physics, Jagiellonian University, Kraków, Poland; Dipartimento di Fisica, Universit di Genova, Genova, Italy; High Energy Physics Institute, Tbilisi State University, Tbilisi, Georgia; Physikalisches Institut, Ruprecht-Karls-Universität Heidelberg, Heidelberg, Germany; ZITI Institut für technische Informatik, Ruprecht-Karls-Universität Heidelberg, Mannheim, Germany; Department of Physics, The University of Hong Kong, Hong Kong, Hong Kong; Department of Physics, The Hong Kong University of Science and Technology, Clear Water Bay, Kowloon, Hong Kong, China; Dipartimento di Matematica e Fisica, Università del Salento, Lecce, Italy; Dipartimento di Fisica, Università di Milano, Milan, Italy; Dipartimento di Fisica, Università di Napoli, Naples, Italy; Dipartimento di Fisica, Università di Pavia, Pavia, Italy; Dipartimento di Fisica E. Fermi, Università di Pisa, Pisa, Italy; Faculdade de Ciências, Universidade de Lisboa, Lisbon, Portugal; Department of Physics, University of Coimbra, Coimbra, Portugal; Centro de Física Nuclear da Universidade de Lisboa, Lisbon, Portugal; Departamento de Fisica, Universidade do Minho, Braga, Portugal; Departamento de Fisica Teorica y del Cosmos and CAFPE, Universidad de Granada, Granada, Spain; Dep Fisica and CEFITEC of Faculdade de Ciencias e Tecnologia, Universidade Nova de Lisboa, Caparica, Portugal; Dipartimento di Fisica, Sapienza Università di Roma, Rome, Italy; Dipartimento di Fisica, Università di Roma Tor Vergata, Rome, Italy; Dipartimento di Matematica e Fisica, Università Roma Tre, Rome, Italy; Centre National de l’Energie des Sciences Techniques Nucleaires, Rabat, Morocco; Faculté des Sciences Semlalia, Université Cadi Ayyad, LPHEA-Marrakech, Marrakech, Morocco; Faculté des Sciences, Université Mohamed Premier and LPTPM, Oujda, Morocco; Faculté des Sciences, Université Mohammed V-Agdal, Rabat, Morocco; Department of Subnuclear Physics, Institute of Experimental Physics of the Slovak Academy of Sciences, Kosice, Slovak Republic; Department of Physics, University of Johannesburg, Johannesburg, South Africa; School of Physics, University of the Witwatersrand, Johannesburg, South Africa; The Oskar Klein Centre, Stockholm, Sweden; Department of Physics and Astronomy, York University, Toronto, ON Canada; ICTP, Trieste, Italy; Dipartimento di Chimica, Fisica e Ambiente, Università di Udine, Udine, Italy

## Abstract

A search for the Standard Model Higgs boson produced in association with a top-quark pair, $t\bar{t}H$, is presented. The analysis uses 20.3 fb^−1^ of *pp* collision data at $\sqrt{s}=8{{\,\mathrm TeV}}$, collected with the ATLAS detector at the Large Hadron Collider during 2012. The search is designed for the $H \rightarrow b\bar{b}$ decay mode and uses events containing one or two electrons or muons. In order to improve the sensitivity of the search, events are categorised according to their jet and *b*-tagged jet multiplicities. A neural network is used to discriminate between signal and background events, the latter being dominated by $t\bar{t}$+jets production. In the single-lepton channel, variables calculated using a matrix element method are included as inputs to the neural network to improve discrimination of the irreducible $t\bar{t}{+}b\bar{b}$ background. No significant excess of events above the background expectation is found and an observed (expected) limit of 3.4 (2.2) times the Standard Model cross section is obtained at 95 % confidence level. The ratio of the measured ${t\bar{t}H}$ signal cross section to the Standard Model expectation is found to be $\mu = 1.5 \pm 1.1$ assuming a Higgs boson mass of 125${{\,\mathrm GeV\,}}$.

## Introduction

The discovery of a new particle in the search for the Standard Model (SM) [1–3] Higgs boson [4–7] at the LHC was reported by the ATLAS [8] and CMS [9] collaborations in July 2012. There is by now clear evidence of this particle in the $H\rightarrow \gamma \gamma$, $H \rightarrow ZZ^{(*)} \rightarrow 4 \ell$, $H \rightarrow WW^{(*)} \rightarrow \ell \nu \ell \nu$ and $H\rightarrow \tau \tau$ decay channels, at a mass of around 125  GeV , which have strengthened the SM Higgs boson hypothesis [10–15] of the observation. To determine all properties of the new boson experimentally, it is important to study it in as many production and decay modes as possible. In particular, its coupling to heavy quarks is a strong focus of current experimental searches. The SM Higgs boson production in association with a top-quark pair (${t\bar{t}H}$) [16–19] with subsequent Higgs decay into bottom quarks (${H\rightarrow b\bar{b}}$) addresses heavy-quark couplings in both production and decay. Due to the large measured mass of the top quark, the Yukawa coupling of the top quark ($y_t$) is much stronger than that of other quarks. The observation of the ${t\bar{t}H}$ production mode would allow for a direct measurement of this coupling, to which other Higgs production modes are only sensitive through loop effects. Since $y_t$ is expected to be close to unity, it is also argued to be the quantity that might give insight into the scale of new physics [20].

The ${H\rightarrow b\bar{b}}$ final state is the dominant decay mode in the SM for a Higgs boson with a mass of 125 GeV. So far, this decay mode has not yet been observed. While a search for this decay via the gluon fusion process is precluded by the overwhelming multijet background, Higgs boson production in association with a vector boson (*VH*)  [21–23] or a top-quark pair ($t\bar{t}$) significantly improves the signal-to-background ratio for this decay.

**Fig. 1:**
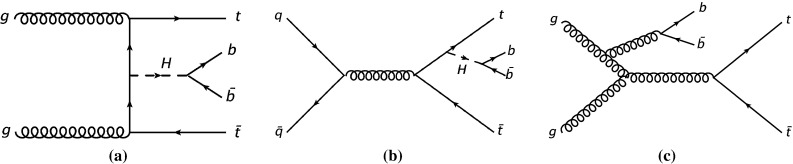
Representative tree-level Feynman diagrams for the production of the Higgs boson in association with a top-quark pair (${t\bar{t}H}$) and the subsequent decay of the Higgs to $b\bar{b}$, (**a**, **b**) for the main background $t\bar{t}{+}b\bar{b}$ (**c**)

This paper describes a search for the SM Higgs boson in the ${t\bar{t}H}$ production mode and is designed to be primarily sensitive to the ${H\rightarrow b\bar{b}}$ decay, although other Higgs boson decay modes are also treated as signal. Figure 1a, b show two examples of tree-level diagrams for ${t\bar{t}H}$ production with a subsequent ${H\rightarrow b\bar{b}}$ decay. A search for the associated production of the Higgs boson with a top-quark pair using several Higgs decay modes (including ${H\rightarrow b\bar{b}}$) has recently been published by the CMS Collaboration [24] quoting a ratio of the measured ${t\bar{t}H}$ signal cross section to the SM expectation for a Higgs boson mass of 125.6${{\,\mathrm GeV\,}}$ of $\mu = 2.8 \pm 1.0$.

The main source of background to this search comes from top-quark pairs produced in association with additional jets. The dominant source is ${t\bar{t}{+}b\bar{b}}$ production, resulting in the same final-state signature as the signal. An example is shown in Fig. 1c. A second contribution arises from $t\bar{t}$ production in association with light-quark (*u*, *d*, *s*) or gluon jets, referred to as $t\bar{t}$+light background, and from $t\bar{t}$ production in association with *c*-quarks, referred to as $t\bar{t}$+$c\bar{c}$. The size of the second contribution depends on the misidentification rate of the algorithm used to identify *b*-quark jets.

The search presented in this paper uses 20.3 fb$^{-1}$ of data collected with the ATLAS detector in *pp* collisions at $\sqrt{s}=8{{\,\mathrm TeV}}$ during 2012. The analysis focuses on final states containing one or two electrons or muons from the decay of the $t\bar{t}$ system, referred to as the single-lepton and dilepton channels, respectively. Selected events are classified into exclusive categories, referred to as “regions”, according to the number of reconstructed jets and jets identified as *b*-quark jets by the *b*-tagging algorithm (*b*-tagged jets or *b*-jets for short). Neural networks (NN) are employed in the regions with a significant expected contribution from the $t\bar{t}H$ signal to separate it from the background. Simpler kinematic variables are used in regions that are depleted of the $t\bar{t}H$ signal, and primarily serve to constrain uncertainties on the background prediction. A combined fit to signal-rich and signal-depleted regions is performed to search for the signal while simultaneously obtaining a background prediction.

## ATLAS detector

The ATLAS detector [25] consists of four main subsystems: an inner tracking system, electromagnetic and hadronic calorimeters, and a muon spectrometer. The inner detector provides tracking information from pixel and silicon microstrip detectors in the pseudorapidity^1^ range $|\eta |<2.5$ and from a straw-tube transition radiation tracker covering $|\eta |<2.0$, all immersed in a 2 T magnetic field provided by a superconducting solenoid. The electromagnetic sampling calorimeter uses lead and liquid-argon (LAr) and is divided into barrel ($|\eta |<1.475$) and end-cap regions ($1.375<|\eta |<3.2$). Hadron calorimetry employs the sampling technique, with either scintillator tiles or liquid argon as active media, and with steel, copper, or tungsten as absorber material. The calorimeters cover $|\eta |<4.9$. The muon spectrometer measures muon tracks within $|\eta |<2.7$ using multiple layers of high-precision tracking chambers located in a toroidal field of approximately 0.5 T and 1 T in the central and end-cap regions of ATLAS, respectively. The muon spectrometer is also instrumented with separate trigger chambers covering $|\eta |<2.4$.

## Object reconstruction

The main physics objects considered in this search are electrons, muons, jets and *b*-jets. Whenever possible, the same object reconstruction is used in both the single-lepton and dilepton channels, though some small differences exist and are noted below.

Electron candidates [26] are reconstructed from energy deposits (clusters) in the electromagnetic calorimeter that are matched to a reconstructed track in the inner detector. To reduce the background from non-prompt electrons, i.e. from decays of hadrons (in particular heavy flavour) produced in jets, electron candidates are required to be isolated. In the single-lepton channel, where such background is significant, an $$ \eta $$-dependent isolation cut is made, based on the sum of transverse energies of cells around the direction of each candidate, in a cone of size $$\Delta R = \sqrt{(\Delta \phi )^2 + (\Delta \eta )^2} = 0.2$$. This energy sum excludes cells associated with the electron and is corrected for leakage from the electron cluster itself. A further isolation cut is made on the scalar sum of the track $${p_{\mathrm {T}}}$$ around the electron in a cone of size $$\Delta R = 0.3 $$ (referred to as $${p_\mathrm {T}^{\mathrm {cone30}}}$$). The longitudinal impact parameter of the electron track with respect to the selected event primary vertex defined in Sect. 4, $$z_{0}$$, is required to be less than 2 mm. To increase efficiency in the dilepton channel, the electron selection is optimised by using an improved electron identification method based on a likelihood variable [27] and the electron isolation. The ratio of $${p_\mathrm {T}^{\mathrm {cone30}}}$$ to the $${p_{\mathrm {T}}}$$ of the electron is required to be less than 0.12, i.e. $${p_\mathrm {T}^{\mathrm {cone30}} /p_{\mathrm {T}}^e}$$$$<$$ 0.12. The optimised selection improves the efficiency by roughly 7 % per electron.

Muon candidates are reconstructed from track segments in the muon spectrometer, and matched with tracks found in the inner detector [28]. The final muon candidates are refitted using the complete track information from both detector systems, and are required to satisfy $|\eta |<2.5$. Additionally, muons are required to be separated by $\Delta R > 0.4$ from any selected jet (see below for details on jet reconstruction and selection). Furthermore, muons must satisfy a ${p_{\mathrm {T}}}$-dependent track-based isolation requirement that has good performance under conditions with a high number of jets from other *pp* interactions within the same bunch crossing, known as “pileup”, or in boosted configurations where the muon is close to a jet: the track ${p_{\mathrm {T}}}$ scalar sum in a cone of variable size $\Delta R < 10{{\,\mathrm GeV\,}}/{p_{\mathrm {T}}}^\mu$ around the muon must be less than 5 % of the muon ${p_{\mathrm {T}}}$. The longitudinal impact parameter of the muon track with respect to the primary vertex, $z_{0}$, is required to be less than 2 mm.

Jets are reconstructed from calibrated clusters [25, 29] built from energy deposits in the calorimeters, using the anti-$k_t$ algorithm [30–32] with a radius parameter $R=0.4$. Prior to jet finding, a local cluster calibration scheme [33, 34] is applied to correct the cluster energies for the effects of dead material, non-compensation and out-of-cluster leakage. The jets are calibrated using energy- and $\eta$-dependent calibration factors, derived from simulations, to the mean energy of stable particles inside the jets. Additional corrections to account for the difference between simulation and data are applied [35]. After energy calibration, jets are required to have ${p_{\mathrm {T}}}> 25{{\,\mathrm GeV\,}}$ and $|\eta | < 2.5$. To reduce the contamination from low-${p_{\mathrm {T}}}$ jets due to pileup, the scalar sum of the ${p_{\mathrm {T}}}$ of tracks matched to the jet and originating from the primary vertex must be at least 50 % of the scalar sum of the ${p_{\mathrm {T}}}$ of all tracks matched to the jet. This is referred to as the jet vertex fraction. This criterion is only applied to jets with ${p_{\mathrm {T}}}< 50{{\,\mathrm GeV\,}}$ and $|\eta |<2.4$.

During jet reconstruction, no distinction is made between identified electrons and jet candidates. Therefore, if any of the jets lie $\Delta R <$ 0.2 from a selected electron, the single closest jet is discarded in order to avoid double-counting of electrons as jets. After this, electrons which are $\Delta R <$ 0.4 from a jet are removed to further suppress background from non-isolated electrons.

Jets are identified as originating from the hadronisation of a *b*-quark via an algorithm [36] that uses multivariate techniques to combine information from the impact parameters of displaced tracks with topological properties of secondary and tertiary decay vertices reconstructed within the jet. The working point used for this search corresponds to a 70 % efficiency to tag a *b*-quark jet, with a light-jet mistag rate of 1 %, and a charm-jet mistag rate of 20 %, as determined for *b*-tagged jets with ${p_{\mathrm {T}}}>20{{\,\mathrm GeV\,}}$ and $|\eta |<2.5$ in simulated $t\bar{t}$ events. Tagging efficiencies in simulation are corrected to match the results of the calibrations performed in data [37]. Studies in simulation show that these efficiencies do not depend on the number of jets.

## Event selection and classification

For this search, only events collected using a single-electron or single-muon trigger under stable beam conditions and for which all detector subsystems were operational are considered. The corresponding integrated luminosity is 20.3 fb$^{-1}$. Triggers with different ${p_{\mathrm {T}}}$ thresholds are combined in a logical OR in order to maximise the overall efficiency. The ${p_{\mathrm {T}}}$ thresholds are 24 or 60  GeV for electrons and 24 or 36  GeV for muons. The triggers with the lower ${p_{\mathrm {T}}}$ threshold include isolation requirements on the lepton candidate, resulting in inefficiency at high ${p_{\mathrm {T}}}$ that is recovered by the triggers with higher ${p_{\mathrm {T}}}$ threshold. The triggers use selection criteria looser than the final reconstruction requirements.

Events accepted by the trigger are required to have at least one reconstructed vertex with at least five associated tracks, consistent with the beam collision region in the *x*–*y* plane. If more than one such vertex is found, the vertex candidate with the largest sum of squared transverse momenta of its associated tracks is taken as the hard-scatter primary vertex.

In the single-lepton channel, events are required to have exactly one identified electron or muon with ${p_{\mathrm {T}}}>25$  GeV and at least four jets, at least two of which are *b*-tagged. The selected lepton is required to match, with $\Delta R < 0.15$, the lepton reconstructed by the trigger.

In the dilepton channel, events are required to have exactly two leptons of opposite charge and at least two *b*-jets. The leading and subleading lepton must have ${p_{\mathrm {T}}}>25$  GeV and ${p_{\mathrm {T}}}>15$  GeV, respectively. Events in the single-lepton sample with additional leptons passing this selection are removed from the single-lepton sample to avoid statistical overlap between the channels. In the dilepton channel, events are categorised into *ee*, $\mu \mu$ and $e\mu$ samples. In the $e\mu$ category, the scalar sum of the transverse energy of leptons and jets, ${{H_{\mathrm {T}}}}$, is required to be above 130 GeV. In the *ee* and $\mu \mu$ event categories, the invariant mass of the two leptons, ${m_{\ell \ell }}$, is required to be larger than 15  GeV in events with more than two *b*-jets, to suppress contributions from the decay of hadronic resonances such as the $J/\psi$ and $\Upsilon$ into a same-flavour lepton pair. In events with exactly two *b*-jets, ${m_{\ell \ell }}$ is required to be larger than 60  GeV due to poor agreement between data and prediction at lower ${m_{\ell \ell }}$. A further cut on ${m_{\ell \ell }}$ is applied in the *ee* and $\mu \mu$ categories to reject events close to the *Z* boson mass: $|{m_{\ell \ell }}- m_Z| > 8$ GeV.

After all selection requirements, the samples are dominated by $t\bar{t}$+jets background. In both channels, selected events are categorised into different regions. In the following, a given region with *m* jets of which *n* are *b*-jets are referred to as “($m \mathrm{j}, n \mathrm{b}$)”. The regions with a signal-to-background ratio $S/B>$ 1 % and $S/\sqrt{B}>0.3$, where *S* and *B* denote the expected signal for a SM Higgs boson with $m_H=125{{\,\mathrm GeV\,}}$, and background, respectively, are referred to as “signal-rich regions”, as they provide most of the sensitivity to the signal. The remaining regions are referred to as “signal-depleted regions”. They are almost purely background-only regions and are used to constrain systematic uncertainties, thus improving the background prediction in the signal-rich regions. The regions are analysed separately and combined statistically to maximise the overall sensitivity. In the most sensitive regions, ${(\ge \!{6} {\mathrm{j}},\ge \!{4} {\mathrm{b}})}$ in the single-lepton channel and ${(\ge \!{4} {\mathrm{j}},\ge \!{4} {\mathrm{b}})}$ in the dilepton channel, $H \rightarrow b\bar{b}$ decays are expected to constitute about 90 % of the signal contribution as shown in Fig. 20 of Appendix A.

**Fig. 2:**
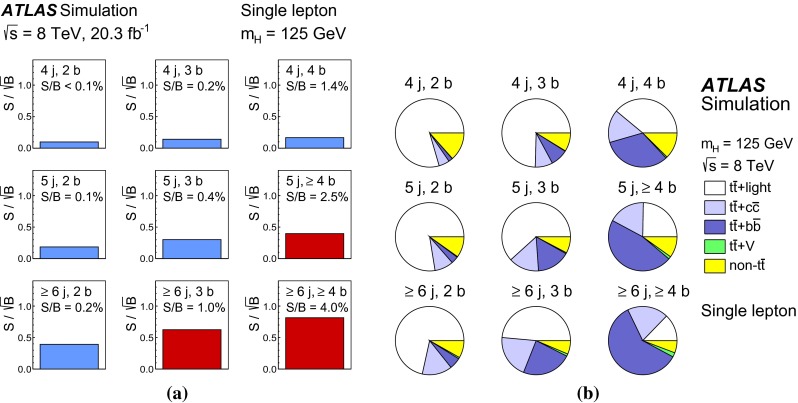
Single-lepton channel: **a** $S/\sqrt{B}$ ratio for each of the regions assuming SM cross sections and branching fractions, and ${m_H}=125{{\,\mathrm GeV\,}}$. *Each row* shows the plots for a specific jet multiplicity (4, 5, $\ge$6), and the *columns* show the *b*-jet multiplicity (2, 3, $\ge$4). Signal-rich regions are *shaded in dark red*, while the rest are shown in *light blue*. The *S* / *B* ratio for each region is also noted. **b** The fractional contributions of the various backgrounds to the total background prediction in each considered region. The ordering of the rows and columns is the same as in a

**Fig. 3:**
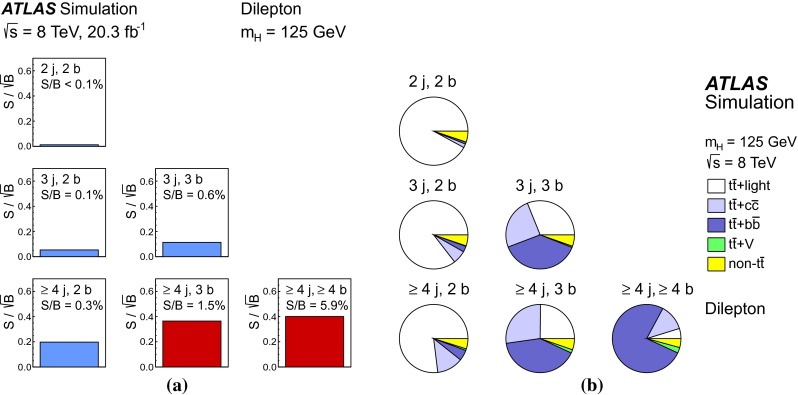
Dilepton channel: **a** The $S/\sqrt{B}$ ratio for each of the regions assuming SM cross sections and branching fractions and ${m_H}=125{{\,\mathrm GeV\,}}$. *Each row* shows the plots for a specific jet multiplicity (2, 3, $\ge$4), and the *columns* show the *b*-jet multiplicity (2, 3, $\ge$4). Signal-rich regions are *shaded in dark red*, while the rest are shown in *light blue*. The *S* / *B* ratio for each region is also noted. **b** The fractional contributions of the various backgrounds to the total background prediction in each considered region. The ordering of the rows and columns is the same as in a

In the single-lepton channel, a total of nine independent regions are considered: six signal-depleted regions $( 4 {\mathrm{j}},\,2 {\mathrm{b}})$, $( 4 {\mathrm{j}},\,2 {\mathrm{b}})$, $( 4 {\mathrm{j}},\,4 {\mathrm{b}})$, $( 5 {\mathrm{j}},\,2 {\mathrm{b}})$, $( 5 {\mathrm{j}},\,3 {\mathrm{b}})$, $({\ge \!{6} {\mathrm{j}},\,2 {\mathrm{b}})}$, and three signal-rich regions, $( 5 {\mathrm{j}},\ge \!{4} {\mathrm{b}})$, ${(\ge \!{6} {\mathrm{j}},\,3 {\mathrm{b}})}$ and ${(\ge \!{6} {\mathrm{j}},\ge \!{4} {\mathrm{b}})}$. In the dilepton channel, a total of six independent regions are considered. The signal-rich regions are ${(\ge \!{4} {\mathrm{j}},\,3 {\mathrm{b}})}$ and ${(\ge \!{4} {\mathrm{j}},\ge \!{4} {\mathrm{b}})}$, while the signal-depleted regions are $(2 {\mathrm{j}},\,2 {\mathrm{b}})$, $(3 {\mathrm{j}},\,2 {\mathrm{b}})$, $(3 {\mathrm{j}},\,3 {\mathrm{b}})$ and ${(\ge \!{4} {\mathrm{j}},\,2 {\mathrm{b}})}$. Figure 2a shows the $S/\sqrt{B}$ and *S* / *B* ratios for the different regions under consideration in the single-lepton channel based on the simulations described in Sect. 5. The expected proportions of different backgrounds in each region are shown in Fig. 2b. The same is shown in the dilepton channel in Fig. 3a, b.

## Background and signal modelling

After the event selection described above, the main background in both the single-lepton and dilepton channels is $t\bar{t}$+jets production. In the single-lepton channel, additional background contributions come from single top quark production, followed by the production of a *W* or *Z* boson in association with jets (*W* / *Z*+jets), diboson (*WW*, *WZ*, *ZZ*) production, as well as the associated production of a vector boson and a $t\bar{t}$ pair, $t\bar{t}{+}V$ ($V=W,Z$). Multijet events also contribute to the selected sample via the misidentification of a jet or a photon as an electron or the presence of a non-prompt electron or muon, referred to as “Lepton misID” background. The corresponding yield is estimated via a data-driven method known as the “matrix method” [38]. In the dilepton channel, backgrounds containing at least two prompt leptons other than $t\bar{t}$+jets production arise from *Z*+jets, diboson, and *Wt*-channel single top quark production, as well as from the $t\bar{t}V$ processes. There are also several processes which may contain either non-prompt leptons that pass the lepton isolation requirements or jets misidentified as leptons. These processes include *W*+jets, $t\bar{t}$ production with a single prompt lepton in the final state, and single top quark production in *t*- and *s*-channels. Their yield is estimated using simulation and cross-checked with a data-driven technique based on the selection of a same-sign lepton pair. In both channels, the contribution of the misidentified lepton background is negligible after requiring two *b*-tagged jets.

In the following, the simulation of each background and of the signal is described in detail. For all MC samples, the top quark mass is taken to be ${m_{t}}= 172.5$  GeV and the Higgs boson mass is taken to be ${m_H}= 125$ GeV.

### $t\bar{t}$+jets background

The $t\bar{t}$+jets sample is generated using the Powheg-Box 2.0 NLO generator [39–41] with the CT10 parton distribution function (PDF) set [42]. It is interfaced to Pythia 6.425 [43] with the CTEQ6L1 PDF set [44] and the Perugia2011C [45] underlying-event tune. The sample is normalised to the top++2.0 [46] theoretical calculation performed at next-to-next-to-leading order (NNLO) in QCD that includes resummation of next-to-next-to-leading logarithmic (NNLL) soft gluon terms [47–51].

The $t\bar{t}$+jets sample is generated inclusively, but events are categorised depending on the flavour of partons that are matched to particle jets that do not originate from the decay of the $t\bar{t}$ system. The matching procedure is done using the requirement of $\Delta R < 0.4$. Particle jets are reconstructed by clustering stable particles excluding muons and neutrinos using the anti-$k_t$ algorithm with a radius parameter $R=0.4$, and are required to have ${p_{\mathrm {T}}}>15{{\,\mathrm GeV\,}}$ and $|\eta |<2.5$.

Events where at least one such particle jet is matched to a bottom-flavoured hadron are labelled as $t\bar{t}{+}b\bar{b}$ events. Similarly, events which are not already categorised as $t\bar{t}{+}b\bar{b}$, and where at least one particle jet is matched to a charm-flavoured hadron, are labelled as $t\bar{t}{+}c\bar{c}$ events. Only hadrons not associated with *b* and *c* quarks from top quark and *W* boson decays are considered. Events labelled as either $t\bar{t}{+}b\bar{b}$ or $t\bar{t}{+}c\bar{c}$ are generically referred to as $t\bar{t}$+HF events (HF for “heavy flavour”). The remaining events are labelled as $t\bar{t}$+light-jet events, including those with no additional jets.

Since Powheg+Pythia only models $t\bar{t}{+}b\bar{b}$ via the parton shower, an alternative $t\bar{t}$+jets sample is generated with the Madgraph5 1.5.11 LO generator [52] using the CT10 PDF set and interfaced to Pythia 6.425 for showering and hadronisation. It includes tree-level diagrams with up to three extra partons (including *b*- and *c*-quarks) and uses settings similar to those in Ref. [24]. To avoid double-counting of partonic configurations generated by both the matrix element calculation and the parton-shower evolution, a parton–jet matching scheme (“MLM matching”) [53] is employed.

Fully matched NLO predictions with massive *b*-quarks have become available recently [54] within the Sherpa with OpenLoops framework [55, 56] referred to in the following as SherpaOL. The SherpaOL NLO sample is generated following the four-flavour scheme using the Sherpa 2.0 pre-release and the CT10 PDF set. The renormalisation scale ($\mu _\mathrm{R}$) is set to $\mu _\mathrm{R}=\prod _{i=t,\bar{t},b,\bar{b}}E_{\mathrm {T},i}^{1/4}$, where $E_{\mathrm {T},i}$ is the transverse energy of parton *i*, and the factorisation and resummation scales are both set to $(E_{\mathrm {T}, t }+E_{\mathrm {T}, \bar{t} })/2$.

For the purpose of comparisons between $t\bar{t}$+jets event generators and the propagation of systematic uncertainties related to the modelling of $t\bar{t}$+HF, as described in Sect. 8.3.1, a finer categorisation of different topologies in $t\bar{t}$+HF is made. In particular, the following categories are considered: if two particle jets are both matched to an extra *b*-quark or extra *c*-quark each, the event is referred to as ${t\bar{t}{+}b\bar{b}}$ or ${t\bar{t}{+}c\bar{c}}$; if a single particle jet is matched to a single *b*(*c*)-quark the event is referred to as $t\bar{t}$+*b* ($t\bar{t}$+*c*); if a single particle jet is matched to a $b\bar{b}$ or a $c\bar{c}$ pair, the event is referred to as $t\bar{t}$+*B* or $t\bar{t}$+*C*, respectively.

**Fig. 4:**
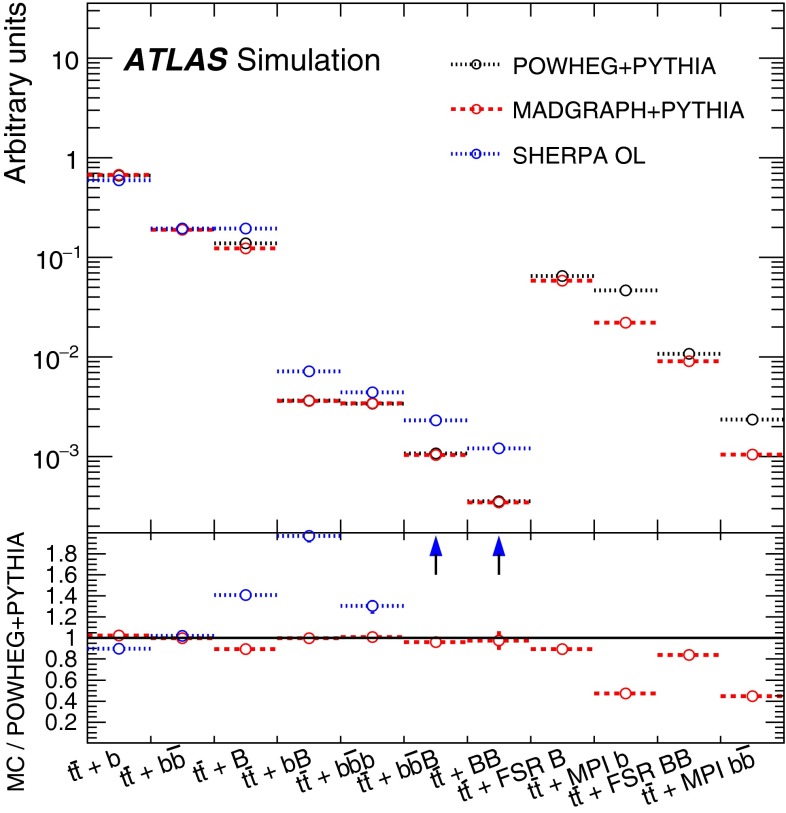
Relative contributions of different categories of $t\bar{t}{+}b\bar{b}$ events in Powheg+Pythia, Madgraph+Pythia and SherpaOL samples. Labels “$t\bar{t}$+MPI” and “$t\bar{t}$+FSR” refer to events where heavy flavour is produced via multiparton interaction (MPI) or final state radiation (FSR), respectively. These contributions are not included in the SherpaOL calculation. An *arrow* indicates that the point is off-scale. Uncertainties are from the limited MC sample sizes

Figure 4 shows the relative contributions of the different $t\bar{t}{+}b\bar{b}$ event categories to the total $t\bar{t}{+}b\bar{b}$ cross section at generator level for the Powheg+Pythia, Madgraph+Pythia and SherpaOL samples. It demonstrates that Powheg+Pythia is able to reproduce reasonably well the $t\bar{t}$+HF content of the Madgraph$t\bar{t}$+jets sample, which includes a LO ${t\bar{t}{+}b\bar{b}}$ matrix element calculation, as well as the NLO SherpaOL prediction.

The relative distribution across categories is such that SherpaOL predicts a higher contribution of the $t\bar{t}+B$ category, as well as every category where the production of a second $b\bar{b}$ pair is required. The modelling of the relevant kinematic variables in each category is in reasonable agreement between Powheg+Pythia and SherpaOL. Some differences are observed in the very low regions of the mass and ${p_{\mathrm {T}}}$ of the $b\bar{b}$ pair, and in the ${p_{\mathrm {T}}}$ of the top quark and $t\bar{t}$ systems.

The prediction from SherpaOL is expected to model the ${t\bar{t}{+}b\bar{b}}$ contribution more accurately than both Powheg+Pythia and Madgraph+Pythia. Thus, in the analysis $t\bar{t}{+}b\bar{b}$ events are reweighted from Powheg+ Pythia to reproduce the NLO $t\bar{t}{+}b\bar{b}$ prediction from SherpaOL for relative contributions of different categories as well as their kinematics. The reweighting is done at generator level using several kinematic variables such as the top quark ${p_{\mathrm {T}}}$, $t\bar{t}$ system ${p_{\mathrm {T}}}$, $\Delta R$ and ${p_{\mathrm {T}}}$ of the dijet system not coming from the top quark decay. In the absence of an NLO calculation of $t\bar{t}{+}c\bar{c}$ production, the Madgraph+Pythia sample is used to evaluate systematic uncertainties on the $t\bar{t}{+}c\bar{c}$ background.

**Fig. 5:**
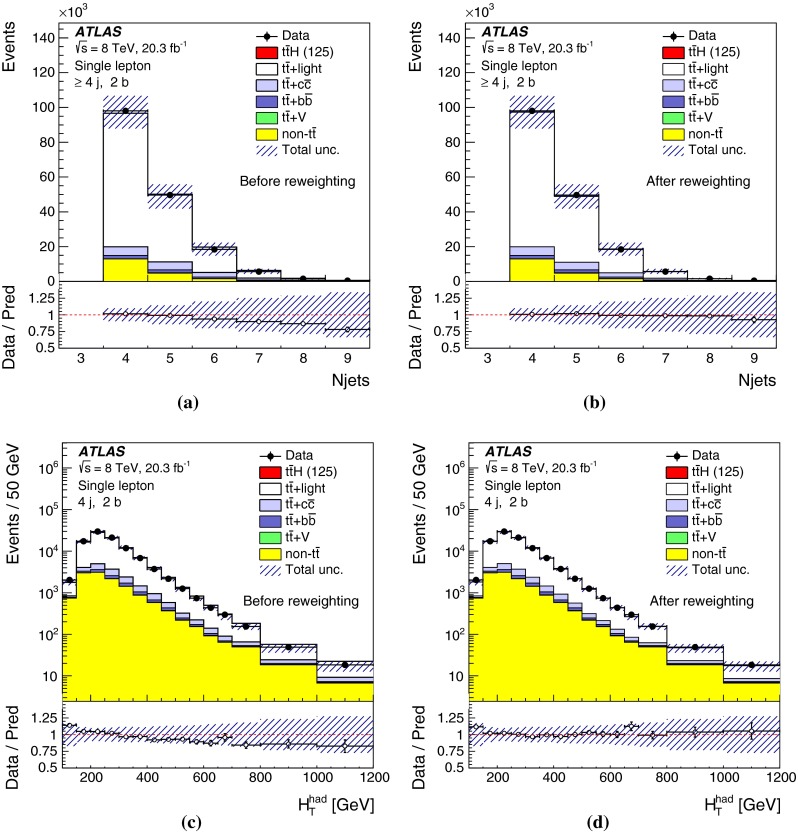
The exclusive 2-*b*-tag region of the single-lepton channel before and after the reweighting of the ${p_{\mathrm {T}}}$ of the $t\bar{t}$ system and the ${p_{\mathrm {T}}}$ of the top quark of the Powheg+Pythia $t\bar{t}$ sample. The jet multiplicity distribution (**a**) before and (**b**) after the reweighting; ${{H_{\mathrm {T}}}^{\mathrm{had}}}$ distribution **c** before and **d** after the reweighting

Since achieving the best possible modelling of the $t\bar{t}$+jets background is a key aspect of this analysis, a separate reweighting is applied to $t\bar{t}$+light and $t\bar{t}{+}c\bar{c}$ events in Powheg+Pythia based on the ratio of measured differential cross sections at $\sqrt{s}=7{{\,\mathrm TeV}}$ in data and simulation as a function of top quark ${p_{\mathrm {T}}}$ and $t\bar{t}$ system ${p_{\mathrm {T}}}$ [57]. It was verified using the simulation that the ratio derived at $\sqrt{s}=7{{\,\mathrm TeV}}$ is applicable to $\sqrt{s}=8{{\,\mathrm TeV}}$ simulation. It is not applied to the $t\bar{t}{+}b\bar{b}$ component since that component was corrected to match the best available theory calculation. Moreover, the measured differential cross section is not sensitive to this component. The reweighting significantly improves the agreement between simulation and data in the total number of jets (primarily due to the $t\bar{t}$ system ${p_{\mathrm {T}}}$ reweighting) and jet ${p_{\mathrm {T}}}$ (primarily due to the top quark ${p_{\mathrm {T}}}$ reweighting). This can be seen in Fig. 5, where the number of jets and the scalar sum of the jet ${p_{\mathrm {T}}}$ (${{H_{\mathrm {T}}}^{\mathrm{had}}}$) distributions in the exclusive 2-*b*-tag region are plotted in the single-lepton channel before and after the reweighting is applied.

### Other backgrounds

The *W* / *Z*+jets background is estimated from simulation reweighted to account for the difference in the *W* / *Z*${p_{\mathrm {T}}}$ spectrum between data and simulation [58]. The heavy-flavour fraction of these simulated backgrounds, i.e. the sum of $W/Z+b\bar{b}$ and $W/Z+c\bar{c}$ processes, is adjusted to reproduce the relative rates of *Z* events with no *b*-tags and those with one *b*-tag observed in data. Samples of *W* / *Z*+jets events, and diboson production in association with jets, are generated using the Alpgen 2.14 [59] leading-order (LO) generator and the CTEQ6L1 PDF set. Parton showers and fragmentation are modelled with Pythia 6.425 for *W* / *Z*+jets production and with Herwig 6.520 [60] for diboson production. The *W*+jets samples are generated with up to five additional partons, separately for *W*+light-jets, $Wb\bar{b}$+jets, $Wc\bar{c}$+jets, and *Wc*+jets. Similarly, the *Z*+jets background is generated with up to five additional partons separated in different parton flavours. Both are normalised to the respective inclusive NNLO theoretical cross section [61]. The overlap between $WQ\bar{Q}$ ($ZQ\bar{Q})$($Q=b,c$) events generated from the matrix element calculation and those from parton-shower evolution in the *W*+light-jet (*Z*+light-jet) samples is removed by an algorithm based on the angular separation between the extra heavy quarks: if $\Delta R(Q,\bar{Q})>0.4$, the matrix element prediction is used, otherwise the parton shower prediction is used.

The diboson+jets samples are generated with up to three additional partons and are normalised to their respecitve NLO theoretical cross sections [62].

Samples of single top quark backgrounds are generated with Powheg-Box 2.0 using the CT10 PDF set. The samples are interfaced to Pythia 6.425 with the CTEQ6L1 set of parton distribution functions and Perugia2011C underlying-event tune. Overlaps between the $t\bar{t}$ and *Wt* final states are removed [63]. The single top quark samples are normalised to the approximate NNLO theoretical cross sections [64–66] using the MSTW2008 NNLO PDF set [67, 68].

Samples of $t\bar{t}{+}V$ are generated with Madgraph 5 and the CTEQ6L1 PDF set. Pythia 6.425 with the AUET2B tune [69] is used for showering. The $t\bar{t}V$ samples are normalised to the NLO cross-section predictions [70, 71].

### Signal model

The $t\bar{t}H$ signal process is modelled using NLO matrix elements obtained from the HELAC-Oneloop package [72]. Powheg-Box serves as an interface to shower Monte Carlo programs. The samples created using this approach are referred to as PowHel samples [73]. They are inclusive in Higgs boson decays and are produced using the CT10nlo PDF set and factorisation ($\mu _\mathrm{F}$) and renormalisation scales set to $\mu _\mathrm{F} = \mu _\mathrm{R} = {m_{t}}+{m_H}/2$. The PowHel${t\bar{t}H}$ sample is showered with Pythia 8.1 [74] with the CTEQ6L1 PDF and the AU2 underlying-event tune [75]. The $t\bar{t}H$ cross section and Higgs boson decay branching fractions are taken from (N)NLO theoretical calculations [19, 76–82], collected in Ref. [83]. In Appendix A, the relative contributions of the Higgs boson decay modes are shown for all regions considered in the analysis.

### Common treatment of MC samples

All samples using Herwig are also interfaced to Jimmy 4.31 [84] to simulate the underlying event. All simulated samples utilise Photos 2.15 [85] to simulate photon radiation and Tauola 1.20 [86] to simulate $\tau$ decays. Events from minimum-bias interactions are simulated with the Pythia 8.1 generator with the MSTW2008 LO PDF set and the AUET2 [87] tune. They are superimposed on the simulated MC events, matching the luminosity profile of the recorded data. The contributions from these pileup interactions are simulated both within the same bunch crossing as the hard-scattering process and in neighbouring bunch crossings.

Finally, all simulated MC samples are processed through a simulation [88] of the detector geometry and response either using Geant4 [89], or through a fast simulation of the calorimeter response [90]. All simulated MC samples are processed through the same reconstruction software as the data. Simulated MC events are corrected so that the object identification efficiencies, energy scales and energy resolutions match those determined from data control samples.

**Fig. 6:**
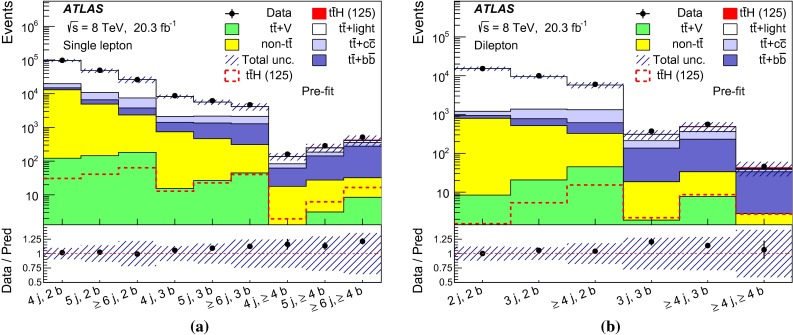
Comparison of prediction to data in all analysis regions before the fit to data in **a** the single-lepton channel and **b** the dilepton channel. The signal, normalised to the SM prediction, is shown both as a *filled red area* stacked on the backgrounds and separately as a *dashed red line*. The *hashed area* corresponds to the total uncertainty on the yields

Figure 6a, b show a comparison of predicted yields to data prior to the fit described in Sect. 9 in all analysis regions in the single-lepton and dilepton channel, respectively. The data agree with the SM expectation within the uncertainties of 10–30 %. Detailed tables of the event yields prior to the fit and the corresponding *S* / *B* and $S/\sqrt{B}$ ratios for the single-lepton and dilepton channels can be found in Appendix B.

When requiring high jet and *b*-tag multiplicity in the analysis, the number of available MC events is significantly reduced, leading to large fluctuations in the resulting distributions for certain samples. This can negatively affect the sensitivity of the analysis through the large statistical uncertainties on the templates and unreliable systematic uncertainties due to shape fluctuations. In order to mitigate this problem, instead of tagging the jets by applying the *b*-tagging algorithm, their probabilities to be *b*-tagged are parameterised as functions of jet flavour, ${p_{\mathrm {T}}}$, and $\eta$. This allows all events in the sample before *b*-tagging is applied to be used in predicting the normalisation and shape after *b*-tagging [91]. The tagging probabilities are derived using an inclusive $t\bar{t}$+jets simulated sample. Since the *b*-tagging probability for a *b*-jet coming from top quark decay is slightly higher than that of a *b*-jet with the same ${p_{\mathrm {T}}}$ and $\eta$ but arising from other sources, they are derived separately. The predictions agree well with the normalisation and shape obtained by applying the *b*-tagging algorithm directly. The method is applied to all signal and background samples.

## Analysis method

In both the single-lepton and dilepton channels, the analysis uses a neural network (NN) to discriminate signal from background in each of the regions with significant expected ${t\bar{t}H}$ signal contribution since the $S/\sqrt{B}$ is very small and the uncertainty on the background is larger than the signal. Those include $( 5 {\mathrm{j}},\ge \!{4} {\mathrm{b}})$, ${(\ge \!{6} {\mathrm{j}},\,3 {\mathrm{b}})}$ and ${(\ge \!{6} {\mathrm{j}},\ge \!{4} {\mathrm{b}})}$ in the case of the single-lepton channel, and ${(\ge \!{4} {\mathrm{j}},\,3 {\mathrm{b}})}$ and ${(\ge \!{4} {\mathrm{j}},\ge \!{4} {\mathrm{b}})}$ in the case of the dilepton channel. In the dilepton channel, an additional NN is used to separate signal from background in the $(3 {\mathrm{j}},\,3 {\mathrm{b}})$ channel. Despite a small expected $S/\sqrt{B}$, it nevertheless adds sensitivity to the signal due to a relatively high expected *S* / *B*. In the single-lepton channel, a dedicated NN is used in the $( 5 {\mathrm{j}},\,3 {\mathrm{b}})$ region to separate $t\bar{t}$+light from $t\bar{t}$+HF backgrounds. The other regions considered in the analysis have lower sensitivity, and use ${{H_{\mathrm {T}}}^{\mathrm{had}}}$ in the single-lepton channel, and the scalar sum of the jet and lepton ${p_{\mathrm {T}}}$ (${{H_{\mathrm {T}}}}$) in the dilepton channel as a discriminant.

The NNs used in the analysis are built using the NeuroBayes [92] package. The choice of the variables that enter the NN discriminant is made through the ranking procedure implemented in this package based on the statistical separation power and the correlation of variables. Several classes of variables were considered: object kinematics, global event variables, event shape variables and object pair properties. In the regions with $\ge$6 ($\ge$4) jets, a maximum of seven (five) jets are considered to construct the kinematic variables in the single-lepton (dilepton) channel, first using all the *b*-jets, and then incorporating the untagged jets with the highest ${p_{\mathrm {T}}}$. All variables used for the NN training and their pairwise correlations are required to be described well in simulation in multiple control regions.

In the $( 5 {\mathrm{j}},\,3 {\mathrm{b}})$ region in the single-lepton channel, the separation between the $t\bar{t}$+light and $t\bar{t}$+HF events is achieved by exploiting the different origin of the third *b*-jet in the case of $t\bar{t}$+light compared to $t\bar{t}$+HF events. In both cases, two of the *b*-jets originate from the $t\bar{t}$ decay. However, in the case of $t\bar{t}$+HF events, the third *b*-jet is likely to originate from one of the additional heavy-flavour quarks, whereas in the case of $t\bar{t}$+light events, the third *b*-jet is often matched to a *c*-quark from the hadronically decaying *W* boson. Thus, kinematic variables, such as the invariant mass of the two untagged jets with minimum $\Delta R$, provide discrimination between $t\bar{t}$+light and $t\bar{t}$+HF events, since the latter presents a distinct peak at the *W* boson mass which is not present in the former. This and other kinematic variables are used in the dedicated NN used in this region.

In addition to the kinematic variables, two variables calculated using the matrix element method (MEM), detailed in Sect. 7, are included in the NN training in ${(\ge \!{6} {\mathrm{j}},\,3 {\mathrm{b}})}$ and ${(\ge \!{6} {\mathrm{j}},\ge \!{4} {\mathrm{b}})}$ regions of the single-lepton channel. These two variables are the Neyman–Pearson likelihood ratio (*D*1) (Eq. (4)) and the logarithm of the summed signal likelihoods (SSLL) (Eq. (2)). The *D*1 variable provides the best separation between ${t\bar{t}H}$ signal and the dominant $t\bar{t}{+}b\bar{b}$ background in the ${(\ge \!{6} {\mathrm{j}},\ge \!{4} {\mathrm{b}})}$ region. The SSLL variable further improves the NN performance.

The variables used in the single-lepton and dilepton channels, as well as their ranking in each analysis region, are listed in Tables 1 and 2, respectively. For the construction of variables in the ${(\ge \!{4} {\mathrm{j}},\ge \!{4} {\mathrm{b}})}$ region of the dilepton channel, the two *b*-jets that are closest in $\Delta R$ to the leptons are considered to originate from the top quarks, and the other two *b*-jets are assigned to the Higgs candidate.

**Table 1:** Single-lepton channel: the definitions and rankings of the variables considered in each of the regions where an NN is used

Variable	Definition	NN rank			
		${\ge }6 \mathrm{j},\,{\ge }4 \mathrm{b}$	${\ge }6 \mathrm{j},\,3 \mathrm{b}$	$5 \mathrm{j},\,{\ge }4 \mathrm{b}$	$5 \mathrm{j},\,3 \mathrm{b}$
*D*1	Neyman–Pearson MEM discriminant (Eq. (4))	1	10	–	–
${{\mathrm{Centrality}}}$	Scalar sum of the ${p_{\mathrm {T}}}$ divided by sum of the *E* for all jets and the lepton	2	2	1	–
${{p_{\mathrm {T}}}^{\mathrm{jet5}}}$	${p_{\mathrm {T}}}$ of the fifth leading jet	3	7	–	–
*H*1	Second Fox–Wolfram moment computed using all jets and the lepton	4	3	2	–
${\Delta R^{\mathrm{avg}}_{\mathrm{bb}}}$	Average $\Delta R$ for all *b*-tagged jet pairs	5	6	5	–
SSLL	Logarithm of the summed signal likelihoods (Eq. (2))	6	4	–	–
${m_{\mathrm{bb}}^{\mathrm{min} \Delta \mathrm{R}}}$	Mass of the combination of the two *b*-tagged jets with the smallest $\Delta R$	7	12	4	4
${m_{\mathrm{bj}}^{\mathrm{max\, p_{T}}}}$	Mass of the combination of a *b*-tagged jet and any jet with the largest vector sum ${p_{\mathrm {T}}}$	8	8	–	–
${\Delta R_{\mathrm{bb}}^{\mathrm{max\, p_{T}}}}$	$\Delta R$ between the two *b*-tagged jets with the largest vector sum ${p_{\mathrm {T}}}$	9	–	–	–
${\Delta R_{\mathrm{lep-bb}}^{\mathrm{min\, \Delta R}}}$	$\Delta R$ between the lepton and the combination of the two *b*-tagged jets with the smallest $\Delta R$	10	11	10	–
${m^{\mathrm{min} \Delta \mathrm{R}}_{\mathrm{uu}}}$	Mass of the combination of the two untagged jets with the smallest $\Delta R$	11	9	–	2
${\mathrm{Aplan_{b-jet}}}$	$1.5 \lambda _2$, where $\lambda _2$ is the second eigenvalue of the momentum tensor [93] built with only *b*-tagged jets	12	–	8	–
${N^{\mathrm{jet}}_{40}}$	Number of jets with ${p_{\mathrm {T}}}\ge 40{{\,\mathrm GeV\,}}$	–	1	3	–
${m_{\mathrm{bj}}^{\mathrm{min\, \Delta R}}}$	Mass of the combination of a *b*-tagged jet and any jet with the smallest $\Delta R$	–	5	–	–
${m_{\mathrm{jj}}^{\mathrm{max\, p_{T}}}}$	Mass of the combination of any two jets with the largest vector sum ${p_{\mathrm {T}}}$	–	–	6	–
${{H_{\mathrm {T}}}^{\mathrm{had}}}$	Scalar sum of jet ${p_{\mathrm {T}}}$	–	–	7	–
${m_{\mathrm{jj}}^{\mathrm{min\, \Delta R}}}$	Mass of the combination of any two jets with the smallest $\Delta R$	–	–	9	–
${m_{\mathrm{bb}}^{\mathrm{max\, p_{T}}}}$	Mass of the combination of the two *b*-tagged jets with the largest vector sum ${p_{\mathrm {T}}}$	–	–	–	1
${ p_{\mathrm{T, uu}}^{\mathrm{min\, \Delta R}}}$	Scalar sum of the ${p_{\mathrm {T}}}$ of the pair of untagged jets with the smallest $\Delta R$	–	–	–	3
${m_{\mathrm{bb}}^{\mathrm{max\, m}}}$	Mass of the combination of the two *b*-tagged jets with the largest invariant mass	–	–	–	5
${\Delta R_{\mathrm{uu}}^{\mathrm{min\, \Delta R}}}$	Minimum $\Delta R$ between the two untagged jets	–	–	–	6
${m_{\mathrm{jjj}}}$	Mass of the jet triplet with the largest vector sum ${p_{\mathrm {T}}}$	–	–	–	7

**Table 2:** Dilepton channel: the definitions and rankings of the variables considered in each of the regions where an NN is used

Variable	Definition	NN rank		
		${\ge }4 \mathrm{j},{\ge }4 \mathrm{b}$	${\ge }4 \mathrm{j},\,3 \mathrm{b}$	$3 \mathrm{j},\,3 \mathrm{b}$
${\Delta \eta _{\mathrm{jj}}^{\mathrm{max\,\Delta \eta }}}$	Maximum $\Delta \eta$ between any two jets in the event	1	1	1
${m_{\mathrm{bb}}^{\mathrm{min} \Delta \mathrm{R}}}$	Mass of the combination of the two *b*-tagged jets with the smallest $\Delta R$	2	8	–
${m_{b\bar{b}}}$	Mass of the two *b*-tagged jets from the Higgs candidate system	3	–	–
${\Delta R_{\mathrm{hl}}^{\mathrm{min\,\Delta R}}}$	$\Delta R$ between the Higgs candidate and the closest lepton	4	5	–
${\mathrm{{N_{30}^{Higgs}}}}$	Number of Higgs candidates within 30 GeV of the Higgs mass of 125 GeV	5	2	5
${\Delta R_{\mathrm{bb}}^{\mathrm{max\, p_{T}}}}$	$\Delta R$ between the two *b*-tagged jets with the largest vector sum ${p_{\mathrm {T}}}$	6	4	8
${\mathrm{Aplan_{jet}}}$	$1.5 \lambda _2$, where $\lambda _2$ is the second eigenvalue of the momentum tensor built with all jets	7	7	–
${m_{\mathrm{jj}}^{\mathrm{min\,m}}}$	Minimum dijet mass between any two jets	8	3	2
${\Delta R_{\mathrm{hl}}^{\mathrm{max}\,\Delta \mathrm{R}}}$	$\Delta R$ between the Higgs candidate and the furthest lepton	9	–	–
${m_{\mathrm{jj}}^{\mathrm{closest}}}$	Dijet mass between any two jets closest to the Higgs mass of 125 GeV	10	–	10
${{H_{\mathrm {T}}}}$	Scalar sum of jet ${p_{\mathrm {T}}}$ and lepton ${p_{\mathrm {T}}}$ values	–	6	3
${\Delta R_{\mathrm{bb}}^{\mathrm{max\,m}}}$	$\Delta R$ between the two *b*-tagged jets with the largest invariant mass	–	9	–
${\Delta R_{\mathrm{lj}}^{\mathrm{min}\,\Delta \mathrm{R}}}$	Minimum $\Delta R$ between any lepton and jet	–	10	–
${{\mathrm{Centrality}}}$	Sum of the ${p_{\mathrm {T}}}$ divided by sum of the *E* for all jets and both leptons	–	–	7
${m_{\mathrm{jj}}^{\mathrm{max\, p_{T}}}}$	Mass of the combination of any two jets with the largest vector sum ${p_{\mathrm {T}}}$	–	–	9
*H*4	Fifth Fox–Wolfram moment computed using all jets and both leptons	–	–	4
${{p_{\mathrm {T}}}^\mathrm{jet3}}$	${p_{\mathrm {T}}}$ of the third leading jet	–	–	6

**Fig. 7:**
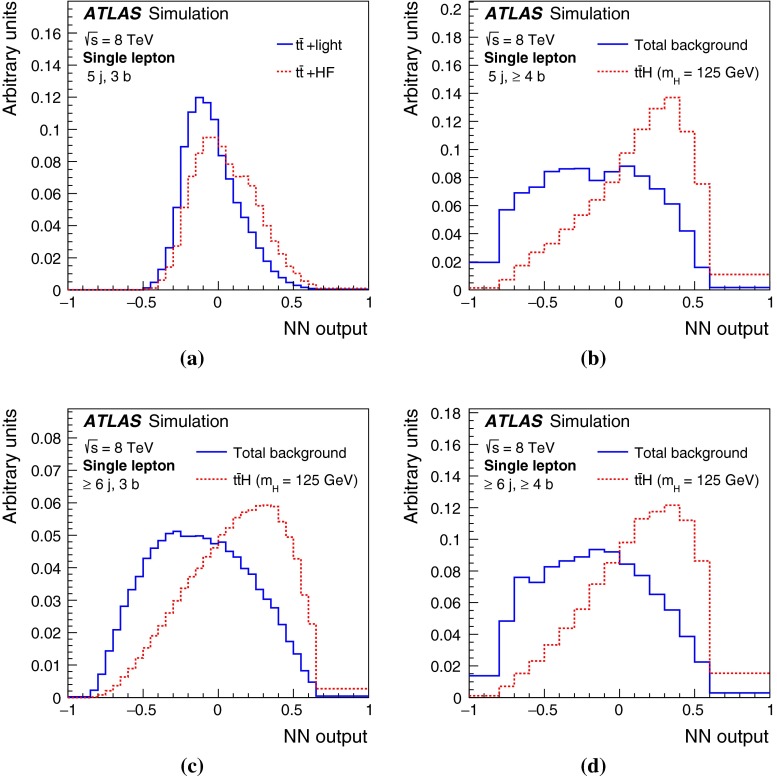
Single-lepton channel: NN output for the different regions. In the $( 5 {\mathrm{j}},\,3 {\mathrm{b}})$ region (**a**), the $t\bar{t}$+HF production is considered as signal and $t\bar{t}$+light as background whereas in the $( 5 {\mathrm{j}},\ge \!{4} {\mathrm{b}})$ (**b**), ${(\ge \!{6} {\mathrm{j}},\,3 {\mathrm{b}})}$ (**c**), and ${(\ge \!{6} {\mathrm{j}},\ge \!{4} {\mathrm{b}})}$ (**d**) regions the NN output is for the ${t\bar{t}H}$ signal and total background. The distributions are normalised to unit area

**Fig. 8:**
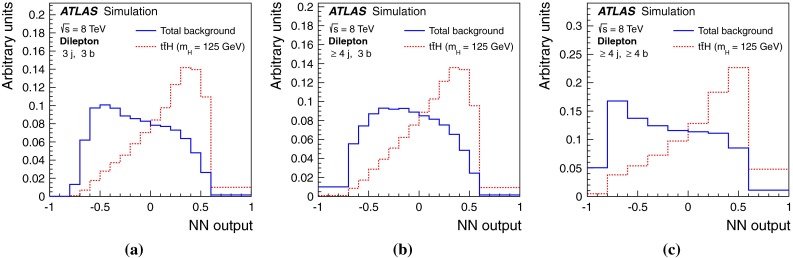
Dilepton channel: NN output for the ${t\bar{t}H}$ signal and total background in the **a** $(3 {\mathrm{j}},\,3 {\mathrm{b}})$, **b** ${(\ge \!{4} {\mathrm{j}},\,3 {\mathrm{b}})}$, and **c** ${(\ge \!{4} {\mathrm{j}},\ge \!{4} {\mathrm{b}})}$ regions. The distributions are normalised to unit area

Figures 7 and 8 show the distribution of the NN discriminant for the ${t\bar{t}H}$ signal and background in the single-lepton and dilepton channels, respectively, in the signal-rich regions. In particular, Fig. 7a shows the separation between the $t\bar{t}$+HF and $t\bar{t}$+light-jet production achieved by a dedicated NN in the $( 5 {\mathrm{j}},\,3 {\mathrm{b}})$ region in the single-lepton channel. The distributions in the highest-ranked input variables from each of the NN regions are shown in Appendix C.

For all analysis regions considered in the fit, the ${t\bar{t}H}$ signal includes all Higgs decay modes. They are also included in the NN training.

The analysis regions have different contributions from various systematic uncertainties, allowing the combined fit to constrain them. The highly populated $( 4 {\mathrm{j}},\,2 {\mathrm{b}})$ and $(2 {\mathrm{j}},\,2 {\mathrm{b}})$ regions in the single-lepton and dilepton channels, respectively, provide a powerful constraint on the overall normalisation of the $t\bar{t}$ background. The $( 4 {\mathrm{j}},\,2 {\mathrm{b}})$, $( 5 {\mathrm{j}},\,2 {\mathrm{b}})$ and $({\ge \!{6} {\mathrm{j}},\,2 {\mathrm{b}})}$ regions in the single-lepton channel and the $(2 {\mathrm{j}},\,2 {\mathrm{b}})$, $(3 {\mathrm{j}},\,2 {\mathrm{b}})$ and ${(\ge \!{4} {\mathrm{j}},\,2 {\mathrm{b}})}$ regions in the dilepton channel are almost pure in $t\bar{t}$+light-jets background and provide an important constraint on $t\bar{t}$ modelling uncertainties both in terms of normalisation and shape. Uncertainties on *c*-tagging are reduced by exploiting the large contribution of $W\rightarrow c s$ decays in the $t\bar{t}$+light-jets background populating the $( 4 {\mathrm{j}},\,3 {\mathrm{b}})$ region in the single-lepton channel. Finally, the consideration of regions with exactly 3 and $\ge$ 4 *b*-jets in both channels, having different fractions of $t\bar{t}{+}b\bar{b}$ and $t\bar{t}{+}c\bar{c}$ backgrounds, provides the ability to constrain uncertainties on the $t\bar{t}{+}b\bar{b}$ and $t\bar{t}{+}c\bar{c}$ normalisations.

## The matrix element method

The matrix element method [94] has been used by the D0 and CDF collaborations for precision measurements of the top quark mass [95, 96] and for the observations of single top quark production [97, 98]. Recently this technique has been used for the ${t\bar{t}H}$ search by the CMS experiment [99]. By directly linking theoretical calculations and observed quantities, it makes the most complete use of the kinematic information of a given event.

The method calculates the probability density function of an observed event to be consistent with physics process *i* described by a set of parameters ${\varvec{\alpha }}$. This probability density function $P_i \left( {\varvec{x}} | {\varvec{\alpha }} \right)$ is defined as

1 $$\begin{aligned}&P_i\left( {\varvec{x}}|{\varvec{\alpha }}\right) = \frac{(2\pi )^4}{\sigma _i^{\mathrm {exp}}\left( {\varvec{\alpha }}\right) } \int \mathrm {d}p_\mathrm {A} \mathrm {d}p_\mathrm {B} \; {\varvec{f}}\left( p_\mathrm {A}\right) {\varvec{f}}\left( p_\mathrm {B}\right) \nonumber \\&\frac{\left| \mathcal {M}_{i}\left( {\varvec{y}}|{\varvec{\alpha }}\right) \right| ^2}{\mathcal {F}} \; W\left( {\varvec{y}}|{\varvec{x}}\right) \; \mathrm {d}\Phi _N\left( {\varvec{y}}\right) \end{aligned}$$

and is obtained by numerical integration over the entire phase space of the initial- and final-state particles. In this equation, ${\varvec{x}}$ and ${\varvec{y}}$ represent the four-momentum vectors of all final-state particles at reconstruction and parton level, respectively. The flux factor $\mathcal {F}$ and the Lorentz-invariant phase space element $\mathrm {d}\Phi _N$ describe the kinematics of the process. The transition matrix element $\mathcal {M}_{i}$ is defined by the Feynman diagrams of the hard process. The transfer functions $W\left( {\varvec{y}}|{\varvec{x}}\right)$ map the detector quantities ${\varvec{x}}$ to the parton level quantities ${\varvec{y}}$. Finally, the cross section $\sigma _i^{\mathrm {exp}}$ normalises $P_i$ to unity taking acceptance and efficiency into account.

The assignment of reconstructed objects to final-state partons in the hard process contains multiple ambiguities. The process probability density is calculated for each allowed assignment permutation of the jets to the final-state quarks of the hard process. A process likelihood function can then be built by summing the process probabilities for the $N_\mathrm{p}$ allowed assignment permutation,

2 $$\begin{aligned} \mathcal {L}_{i} \left( {\varvec{x}}|{\varvec{\alpha }}\right) = \sum _{p=1}^{N_\mathrm{p}} P_{i}^\mathrm{p} \left( {\varvec{x}}|{\varvec{\alpha }} \right) . \end{aligned}$$

The process probability densities are used to distinguish signal from background events by calculating the likelihood ratio of the signal and background processes contributing with fractions $f_{\text {bkg}}$,

3 $$\begin{aligned} r_{\text {sig}} \left( {\varvec{x}} | {\varvec{\alpha }} \right) = \frac{\mathcal {L}_{\text {sig}} \left( {\varvec{x}}|{\varvec{\alpha }} \right) }{\sum \limits _{\text {bkg}} f_{\text {bkg}} \mathcal {L}_{\text {bkg}} \left( {\varvec{x}}|{\varvec{\alpha }} \right) } . \end{aligned}$$

This ratio, according to the Neyman–Pearson lemma [100], is the most powerful discriminant between signal and background processes. In the analysis, this variable is used as input to the NN along with other kinematic variables.

Matrix element calculation methods are generated with Madgraph 5 in LO. The transfer functions are obtained from simulation following a similar procedure as described in Ref. [101]. For the modelling of the parton distribution functions the CTEQ6L1 set from the LHAPDF package [102] is used.

The integration is performed using VEGAS [103]. Due to the complexity and high dimensionality, adaptive MC techniques [104], simplifications and approximations are needed to obtain results within a reasonable computing time. In particular, only the numerically most significant contributing helicity states of a process hypothesis for a given event, identified at the start of each integration, are evaluated. This does not perceptibly decrease the separation power but reduces the calculation time by more than an order of magnitude. Furthermore, several approximations are made to improve the VEGAS convergence rate. Firstly, the dimensionality of integration is reduced by assuming that the final-state object directions in ${\eta }$ and ${\phi }$ as well as charged lepton momenta are well measured, and therefore the corresponding transfer functions are represented by $\delta$ functions. The total momentum conservation and a negligible transverse momentum of the initial-state partons allow for further reduction. Secondly, kinematic transformations are utilised to optimise the integration over the remaining phase space by aligning the peaks of the integrand with the integration dimensions. The narrow-width approximation is applied to the leptonically decaying *W* boson. This leaves three *b*-quark energies, one light-quark energy, the hadronically decaying *W* boson mass and the invariant mass of the two *b*-quarks originating from either the Higgs boson for the signal or a gluon for the background as the remaining parameters which define the integration phase space. The total integration volume is restricted based upon the observed values and the width of the transfer functions and of the propagator peaks in the matrix elements. Finally, the likelihood contributions of all allowed assignment permutations are coarsely integrated, and only for the leading twelve assignment permutations is the full integration performed, with a required precision decreasing according to their relative contributions.

The signal hypothesis is defined as a SM Higgs boson produced in association with a top-quark pair as shown in Fig. 1a, b. Hence no coupling of the Higgs boson to the *W* boson is accounted for in $| \mathcal {M}_{i}|^{2}$ to allow for a consistent treatment when performing the kinematic transformation. The Higgs boson is required to decay into a pair of *b*-quarks, while the top-quark pair decays into the single-lepton channel. For the background hypothesis, only the diagrams of the irreducible ${t\bar{t}{+}b\bar{b}}$ background are considered. Since it dominates the most signal-rich analysis regions, inclusion of other processes does not improve the separation between signal and background. No gluon radiation from the final-state quarks is allowed, since these are kinematically suppressed and difficult to treat in any kinematic transformation aiming for phase-space alignment during the integration process. In the definition of the signal and background hypothesis the LO diagrams are required to have a top-quark pair as an intermediate state resulting in exactly four *b*-quarks, two light quarks, one charged lepton (electron or muon) and one neutrino in the final state. Assuming lepton universality and invariance under charge conjugation, diagrams of only one lepton flavour and of only negative charge (electron) are considered. The probability density function calculation of the signal and background is only performed in the ${(\ge \!{6} {\mathrm{j}},\,3 {\mathrm{b}})}$ and ${(\ge \!{6} {\mathrm{j}},\ge \!{4} {\mathrm{b}})}$ regions of the single-lepton channel. Only six reconstructed jets are considered in the calculation: the four jets with the highest value of the probability to be a *b*-jet returned by the *b*-tagging algorithm (i.e. the highest *b*-tagging weight) and two of the remaining jets with an invariant mass closest to the *W* boson mass of 80.4 GeV. If a jet is *b*-tagged it cannot be assigned to a light quark in the matrix element description. In the case of more than four *b*-tagged jets, only the four with the highest *b*-tagging weight are treated as *b*-tagged. Assignment permutations between the two light quarks of the hadronically decaying *W* boson and between the two *b*-quarks originating from the Higgs boson or gluon result in the same likelihood value and are thus not considered. As a result there are in total 12 and 36 assignment permutations in the ${(\ge \!{6} {\mathrm{j}},\ge \!{4} {\mathrm{b}})}$ and ${(\ge \!{6} {\mathrm{j}},\,3 {\mathrm{b}})}$ region, respectively, which need to be evaluated in the coarse integration phase.

Using the ${t\bar{t}H}$ process as the signal hypothesis and the ${t\bar{t}{+}b\bar{b}}$ process as the background hypothesis, a slightly modified version of Eq. (3) is used to define the likelihood ratio *D*1:

4 $$\begin{aligned} D1=\frac{\mathcal {L}_{t\bar{t}H}}{{\mathcal {L}_{t\bar{t}H}} + \alpha \cdot \mathcal {L}_{t\bar{t}{+}b\bar{b}}} , \end{aligned}$$

where $\alpha =0.23$ is a relative normalisation factor chosen to optimise the performance of the discriminant given the finite bin sizes of the *D*1 distribution. In this definition, signal-like and background-like events have *D*1 values close to one and zero, respectively. The logarithm of the summed signal likelihoods defined by Eq. (2) and the ratio *D*1 are included in the NN training in both the ${(\ge \!{6} {\mathrm{j}},\,3 {\mathrm{b}})}$ and ${(\ge \!{6} {\mathrm{j}},\ge \!{4} {\mathrm{b}})}$ regions.

## Systematic uncertainties

Several sources of systematic uncertainty are considered that can affect the normalisation of signal and background and/or the shape of their final discriminant distributions. Individual sources of systematic uncertainty are considered uncorrelated. Correlations of a given systematic effect are maintained across processes and channels. Table 3 presents a summary of the sources of systematic uncertainty considered in the analysis, indicating whether they are taken to be normalisation-only, shape-only, or to affect both shape and normalisation. In Appendix D, the normalisation impact of the systematic uncertainties are shown on the $t\bar{t}$ background as well as on the ${t\bar{t}H}$ signal.

In order to reduce the degradation of the sensitivity of the search due to systematic uncertainties, they are fitted to data in the statistical analysis, exploiting the constraining power from the background-dominated regions described in Sect. 4. Each systematic uncertainty is represented by an independent parameter, referred to as a “nuisance parameter”, and is fitted with a Gaussian prior for the shape differences and a log-normal distribution for the normalisation. They are centred around zero with a width that corresponds to the given uncertainty.

**Table 3:** List of systematic uncertainties considered. An “N” means that the uncertainty is taken as normalisation-only for all processes and channels affected, whereas an “S” denotes systematic uncertainties that are considered shape-only in all processes and channels. An “SN” means that the uncertainty is taken on both shape and normalisation. Some of the systematic uncertainties are split into several components for a more accurate treatment. This is the number indicated in the column labelled as “Comp.”

Systematic uncertainty	Type	Comp.
Luminosity	N	1
*Physics objects*		
Electron	SN	5
Muon	SN	6
Jet energy scale	SN	22
Jet vertex fraction	SN	1
Jet energy resolution	SN	1
Jet reconstruction	SN	1
*b*-tagging efficiency	SN	6
*c*-tagging efficiency	SN	4
Light-jet tagging efficiency	SN	12
High-${p_{\mathrm {T}}}$ tagging efficiency	SN	1
*Background model*		
$t\bar{t}$ cross section	N	1
$t\bar{t}$ modelling: ${p_{\mathrm {T}}}$ reweighting	SN	9
$t\bar{t}$ modelling: parton shower	SN	3
$t\bar{t}$+heavy-flavour: normalisation	N	2
$t\bar{t}$+$c\bar{c}$: ${p_{\mathrm {T}}}$ reweighting	SN	2
$t\bar{t}$+$c\bar{c}$: generator	SN	4
$t\bar{t}$+$b\bar{b}$: NLO Shape	SN	8
*W*+jets normalisation	N	3
*W* ${p_{\mathrm {T}}}$ reweighting	SN	1
*Z*+jets normalisation	N	3
*Z* ${p_{\mathrm {T}}}$ reweighting	SN	1
Lepton misID normalisation	N	3
Lepton misID shape	S	3
Single top cross section	N	1
Single top model	SN	1
Diboson+jets normalisation	N	3
$t\bar{t}+V$ cross section	N	1
$t\bar{t}+V$ model	SN	1
*Signal model*		
$t\bar{t}H$ scale	SN	2
$t\bar{t}H$ generator	SN	1
$t\bar{t}H$ hadronisation	SN	1
$t\bar{t}H$ PDF	SN	1

### Luminosity

The uncertainty on the integrated luminosity for the data set used in this analysis is 2.8 %. It is derived following the same methodology as that detailed in Ref. [105]. This systematic uncertainty is applied to all contributions determined from the MC simulation.

### Uncertainties on physics objects

#### Leptons

Uncertainties associated with the lepton selection arise from the trigger, reconstruction, identification, isolation and lepton momentum scale and resolution. In total, uncertainties associated with electrons (muons) include five (six) components.

#### Jets

Uncertainties associated with the jet selection arise from the jet energy scale (JES), jet vertex fraction requirement, jet energy resolution and jet reconstruction efficiency. Among these, the JES uncertainty has the largest impact on the analysis. The JES and its uncertainty are derived combining information from test-beam data, LHC collision data and simulation [35]. The jet energy scale uncertainty is split into 22 uncorrelated sources which can have different jet ${p_{\mathrm {T}}}$ and $\eta$ dependencies. In this analysis, the largest jet energy scale uncertainty arises from the $\eta$ dependence of the JES calibration in the end-cap regions of the calorimeter. It is the second leading uncertainty.

#### Heavy- and light-flavour tagging

A total of six (four) independent sources of uncertainty affecting the *b*(*c*)-tagging efficiency are considered [37]. Each of these uncertainties corresponds to an eigenvector resulting from diagonalising the matrix containing the information about the total uncertainty per jet ${p_{\mathrm {T}}}$ bin and the bin-to-bin correlations. An additional uncertainty is assigned due to the extrapolation of the *b*-tagging efficiency measurement to the high-${p_{\mathrm {T}}}$ region. Twelve uncertainties are considered for the light-jet tagging and they depend on jet ${p_{\mathrm {T}}}$ and $\eta$. These systematic uncertainties are taken as uncorrelated between *b*-jets, *c*-jets, and light-flavour jets.

No additional systematic uncertainty is assigned due to the use of parameterisations of the *b*-tagging probabilities instead of applying the *b*-tagging algorithm directly since the difference between these two approaches is negligible compared to the other sources.

### Uncertainties on background modelling

#### $t\bar{t}{+}jets$ modelling

An uncertainty of +6.5 %/–6 % is assumed for the inclusive $t\bar{t}$ production cross section. It includes uncertainties from the top quark mass and choices of the PDF and $\alpha _{\mathrm {S}}$. The PDF and $\alpha _{\mathrm {S}}$ uncertainties are calculated using the PDF4LHC prescription [106] with the MSTW2008 68 % CL NNLO, CT10 NNLO [107] and NNPDF2.3 5f FFN [108] PDF sets, and are added in quadrature to the scale uncertainty. Other systematic uncertainties affecting the modelling of $t\bar{t}$+jets include uncertainties due to the choice of parton shower and hadronisation model, as well as several uncertainties related to the reweighting procedure applied to improve the ${t\bar{t}}$ MC model. Additional uncertainties are assigned to account for limited knowledge of $t\bar{t}$+HF jets production. They are described later in this section.

As discussed in Sect. 5, to improve the agreement between data and the $t\bar{t}$ simulation a reweighting procedure is applied to $t\bar{t}$ MC events based on the difference in the top quark ${p_{\mathrm {T}}}$ and $t\bar{t}$ system ${p_{\mathrm {T}}}$ distributions between data and simulation at $\sqrt{s}=7{{\,\mathrm TeV}}$ [57]. The nine largest uncertainties associated with the experimental measurement of top quark and $t\bar{t}$ system ${p_{\mathrm {T}}}$, representing approximately 95 % of the total experimental uncertainty on the measurement, are considered as separate uncertainty sources in the reweighting applied to the MC prediction. The largest uncertainties on the measurement of the differential distributions include radiation modelling in $t\bar{t}$ events, the choice of generator to simulate $t\bar{t}$ production, uncertainties on the components of jet energy scale and resolution, and flavour tagging.

Because the measurement is performed for the inclusive $t\bar{t}$ sample and the size of the uncertainties applicable to the $t\bar{t}{+}c\bar{c}$ component is not known, two additional uncorrelated uncertainties are assigned to $t\bar{t}{+}c\bar{c}$ events, consisting of the full difference between applying and not applying the reweightings of the $t\bar{t}$ system ${p_{\mathrm {T}}}$ and top quark ${p_{\mathrm {T}}}$, respectively.

An uncertainty due to the choice of parton shower and hadronisation model is derived by comparing events produced by Powheg interfaced with Pythia or Herwig. Effects on the shapes are compared, symmetrised and applied to the shapes predicted by the default model. Given that the change of the parton shower model leads to two separate effects – a change in the number of jets and a change of the heavy-flavour content – the parton shower uncertainty is represented by three parameters, one acting on the $t\bar{t}$+light contribution and two others on the $t\bar{t}{+}c\bar{c}$ and $t\bar{t}{+}b\bar{b}$ contributions. These three parameters are treated as uncorrelated in the fit.

Detailed comparisons of $t\bar{t}{+}b\bar{b}$ production between Powheg+Pythia and an NLO prediction of $t\bar{t}{+}b\bar{b}$ production based on SherpaOL have shown that the cross sections agree within 50 % of each other. Therefore, a systematic uncertainty of 50 % is applied to the ${t\bar{t}{+}b\bar{b}}$ component of the $t\bar{t}$+jets background obtained from the Powheg+Pythia MC simulation. In the absence of an NLO prediction for the $t\bar{t}{+}c\bar{c}$ background, the same 50 % systematic uncertainty is applied to the $t\bar{t}{+}c\bar{c}$ component, and the uncertainties on ${t\bar{t}{+}b\bar{b}}$ and $t\bar{t}{+}c\bar{c}$ are treated as uncorrelated. The large available data sample allows the determination of the $t\bar{t}{+}b\bar{b}$ and $t\bar{t}{+}c\bar{c}$ normalisations with much better precision, approximately 15 and 30 %, respectively (see Appendix D). Thus, the final result does not significantly depend on the exact value of the assumed prior uncertainty, as long as it is larger than the precision with which the data can constrain it. However, even after the reduction, the uncertainties on the $t\bar{t}{+}b\bar{b}$ and the $t\bar{t}{+}c\bar{c}$ background normalisation are still the leading and the third leading uncertainty in the analysis, respectively.

Four additional systematic uncertainties in the $t\bar{t}{+}c\bar{c}$ background estimate are derived from the simultaneous variation of factorisation and renormalisation scales, matching threshold and *c*-quark mass variations in the Madgraph+Pythia$t\bar{t}$ simulation, and the difference between the $t\bar{t}{+}c\bar{c}$ simulation in Madgraph+Pythia and Powheg+Pythia since Madgraph+Pythia includes the $t\bar{t}{+}c\bar{c}$ process in the matrix element calculation while it is absent in Powheg+Pythia.

**Fig. 9:**
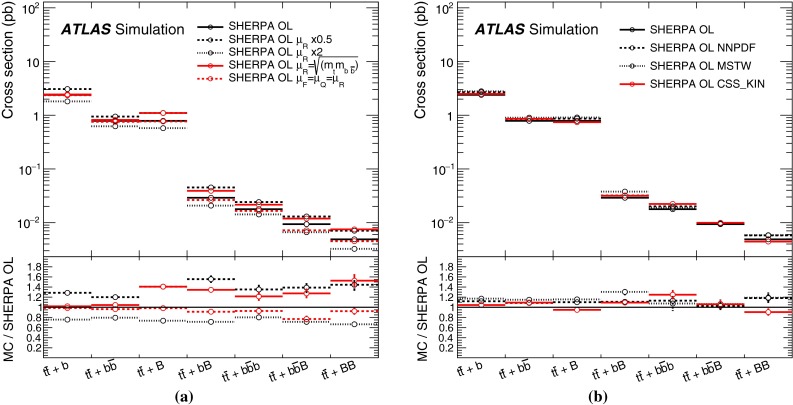
Systematic uncertainties on the $t\bar{t}{+}b\bar{b}$ contribution based on **a** scale variations and **b** PDF choice and shower recoil model of the SherpaOL simulation. The effect of a given systematic uncertainty is shown across the different $t\bar{t}{+}b\bar{b}$ categories. The effect of migration between categories is covered by variations of these systematic uncertainties

For the $t\bar{t}{+}b\bar{b}$ background, three scale uncertainties, including changing the functional form of the renormalisation scale to $\mu _\mathrm{R} = (m_t m_{b\bar{b}})^{1/2}$, changing the functional form of the factorisation $\mu _\mathrm{F}$ and resummation $\mu _Q$ scales to $\mu _\mathrm{F} =\mu _\mathrm{Q} =\prod _{i=t,\bar{t},b,\bar{b}}E_\mathrm{{T},i}^{1/4}$ and varying the renormalisation scale $\mu _\mathrm{R}$ by a factor of two up and down are evaluated. Additionally, the shower recoil model uncertainty and two uncertainties due to the PDF choice in the SherpaOL NLO calculation are quoted. The effect of these variations on the contribution of different $t\bar{t}{+}b\bar{b}$ event categories is shown in Fig. 9. The renormalisation scale choice and the shower recoil scheme have a large effect on the modelling of ${t\bar{t}{+}b\bar{b}}$. They provide large shape variations of the NN discriminants resulting in the fourth and sixth leading uncertainties in this analysis.

Finally, two uncertainties due to $t\bar{t}{+}b\bar{b}$ production via multiparton interaction and final-state radiation which are not present in the SherpaOL NLO calculation are applied. Overall, the uncertainties on ${t\bar{t}{+}b\bar{b}}$ normalisation and modelling result in about a 55 % total uncertainty on the ${t\bar{t}{+}b\bar{b}}$ background contribution in the most sensitive ${(\ge \!{6} {\mathrm{j}},\ge \!{4} {\mathrm{b}})}$ and ${(\ge \!{4} {\mathrm{j}},\ge \!{4} {\mathrm{b}})}$ regions.

#### The *W* / *Z*+jets modelling

As discussed in Sect. 5, the *W* / *Z*+jets contributions are obtained from the simulation and normalised to the inclusive theoretical cross sections, and a reweighting is applied to improve the modelling of the *W* / *Z* boson ${p_{\mathrm {T}}}$ spectrum. The full difference between applying and not applying the *W* / *Z* boson ${p_{\mathrm {T}}}$ reweighting is taken as a systematic uncertainty, which is then assumed to be symmetric with respect to the central value. Additional uncertainties are assigned due to the extrapolation of the *W* / *Z*+jets estimate to high jet multiplicity.

#### Misidentified lepton background modelling

Systematic uncertainties on the misidentified lepton background estimated via the matrix method [38] in the single-lepton channel receive contributions from the limited number of data events, particularly at high jet and *b*-tag multiplicities, from the subtraction of the prompt-lepton contribution as well as from the uncertainty on the lepton misidentification rates, estimated in different control regions. The statistical uncertainty is uncorrelated among the different jet and *b*-tag multiplicity bins. An uncertainty of 50 % associated with the lepton misidentification rate measurements is assumed, which is taken as correlated across jet and *b*-tag multiplicity bins, but uncorrelated between electron and muon channels. Uncertainty on the shape of the misidentified lepton background arises from the prompt-lepton background subtraction and the misidentified lepton rate measurement.

In the dilepton channel, since the misidentified lepton background is estimated using both the simulation and same-sign dilepton events in data, a 50 % normalisation uncertainty is assigned to cover the maximum difference between the two methods. It is taken as correlated among the different jet and *b*-tag multiplicity bins. An additional uncertainty is applied to cover the difference in shape between the predictions derived from the simulation and from same-sign dilepton events in data.

#### Electroweak background modelling

Uncertainties of +5 %/–4 % and $\pm 6.8$ % are used for the theoretical cross sections of single top production in the single-lepton and dilepton channels [64, 65], respectively. The former corresponds to the weighted average of the theoretical uncertainties on *s*-, *t*- and *Wt*-channel production, while the latter corresponds to the theoretical uncertainty on *Wt*-channel production, the only single top process contributing to the dilepton final state.

The uncertainty on the diboson background rates includes an uncertainty on the inclusive diboson NLO cross section of $\pm 5\,\%$ [62] and uncertainties to account for the extrapolation to high jet multiplicity.

Finally, an uncertainty of $\pm 30\,\%$ is assumed for the theoretical cross sections of the $t\bar{t}{+}V$ [70, 71] background. An additional uncertainty on $t\bar{t}{+}V$ modelling arises from variations in the amount of initial-state radiation. The $t\bar{t}+Z$ background with *Z* boson decaying into a $b\bar{b}$ pair is an irreducible background to the ${t\bar{t}H}$, ${H\rightarrow b\bar{b}}$ signal, and as such, has kinematics and an NN discriminant shape similar to those of the signal. The uncertainty on the $t\bar{t}{+}V$ background normalisation is the fifth leading uncertainty in the analysis.

### Uncertainties on signal modelling

Dedicated NLO PowHel samples are used to evaluate the impact of the choice of factorisation and renormalisation scales on the $t\bar{t}H$ signal kinematics. In these samples the default scale is varied by a factor of two up and down. The effect of the variations on $t\bar{t}H$ distributions was studied at particle level and the nominal PowHel$t\bar{t}H$ sample was reweighted to reproduce these variations. In a similar way, the nominal sample is reweighted to reproduce the effect of changing the functional form of the scale. Additional uncertainties on the ${t\bar{t}H}$ signal due to the choice of PDF, parton shower and fragmentation model and NLO generator are also considered. The effect of the PDF uncertainty on the ${t\bar{t}H}$ signal is evaluated following the recommendation of the PDF4LHC. The uncertainty in the parton shower and fragmentation is evaluated by comparing Powhel+Pythia8 and Powhel+Herwig samples, while the uncertainty due to a generator choice is evaluated by comparing Powhel+Pythia8 with Madgraph5_aMC@NLO [109] interfaced with Herwig++ [110, 111].

## Statistical methods

The distributions of the discriminants from each of the channels and regions considered are combined to test for the presence of a signal, assuming a Higgs boson mass of ${m_H}= 125 {{\,\mathrm GeV\,}}$. The statistical analysis is based on a binned likelihood function $\mathcal{L}(\mu ,\theta )$ constructed as a product of Poisson probability terms over all bins considered in the analysis. The likelihood function depends on the signal-strength parameter $\mu$, defined as the ratio of the observed/expected cross section to the SM cross section, and $\theta$, denoting the set of nuisance parameters that encode the effects of systematic uncertainties on the signal and background expectations. They are implemented in the likelihood function as Gaussian or log-normal priors. Therefore, the total number of expected events in a given bin depends on $\mu$ and $\theta$. The nuisance parameters $\theta$ adjust the expectations for signal and background according to the corresponding systematic uncertainties, and their fitted values correspond to the amount that best fits the data. This procedure allows the impact of systematic uncertainties on the search sensitivity to be reduced by taking advantage of the highly populated background-dominated control regions included in the likelihood fit. It requires a good understanding of the systematic effects affecting the shapes of the discriminant distributions. The test statistic $q_\mu$ is defined as the profile likelihood ratio: $q_\mu = -2\ln (\mathcal{L}(\mu ,\hat{\hat{\theta }}_\mu )/\mathcal{L}(\hat{\mu },\hat{\theta }))$, where $\hat{\mu }$ and $\hat{\theta }$ are the values of the parameters that maximise the likelihood function (with the constraints $0\le \hat{\mu } \le \mu$), and $\hat{\hat{\theta }}_\mu$ are the values of the nuisance parameters that maximise the likelihood function for a given value of $\mu$. This test statistic is used to measure the compatibility of the observed data with the background-only hypothesis (i.e. for $\mu =0$), and to make statistical inferences about $\mu$, such as upper limits using the CL$_\mathrm{{s}}$ method [112–114] as implemented in the RooFit package [115, 116].

To obtain the final result, a simultaneous fit to the data is performed on the distributions of the discriminants in 15 regions: nine analysis regions in the single-lepton channel and six regions in the dilepton channel. Fits are performed under the signal-plus-background hypothesis, where the signal-strength parameter $\mu$ is the parameter of interest in the fit and is allowed to float freely, but is required to be the same in all 15 fit regions. The normalisation of each background is determined from the fit simultaneously with $\mu$. Contributions from $t\bar{t}$, *W* / *Z*+jets production, single top, diboson and $t\bar{t}V$ backgrounds are constrained by the uncertainties of the respective theoretical calculations, the uncertainty on the luminosity, and the data themselves. Statistical uncertainties in each bin of the discriminant distributions are taken into account by dedicated parameters in the fit. The performance of the fit is tested using simulated events by injecting ${t\bar{t}H}$ signal with a variable signal strength and comparing it to the fitted value. Good agreement between the injected and measured signal strength is observed.

## Results

**Fig. 10:**
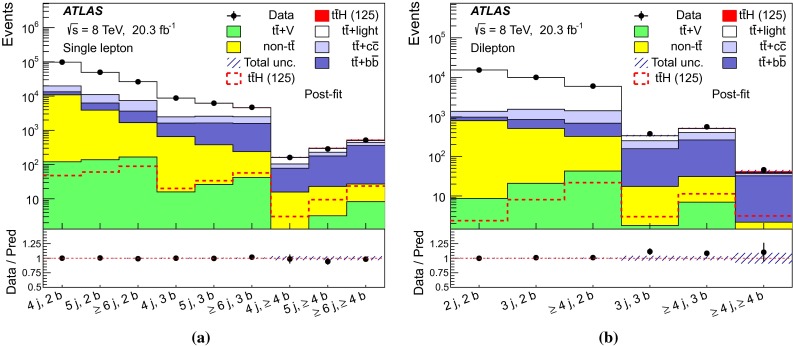
Event yields in all analysis regions in **a** the single-lepton channel and **b** the dilepton channel after the combined fit to data under the signal-plus-background hypothesis. The signal, normalised to the fitted $\mu$, is shown both as a *filled area* stacked on the other backgrounds and separately as a *dashed line*. The *hashed area* represents the total uncertainty on the yields

The results of the binned likelihood fit to data described in Sect. 9 are presented in this section. Figure 10 shows the yields after the fit in all analysis regions in the single-lepton and dilepton channels. The post-fit event yields and the corresponding *S* / *B* and $S/\sqrt{B}$ ratios are summarised in Appendix E.

**Fig. 11:**
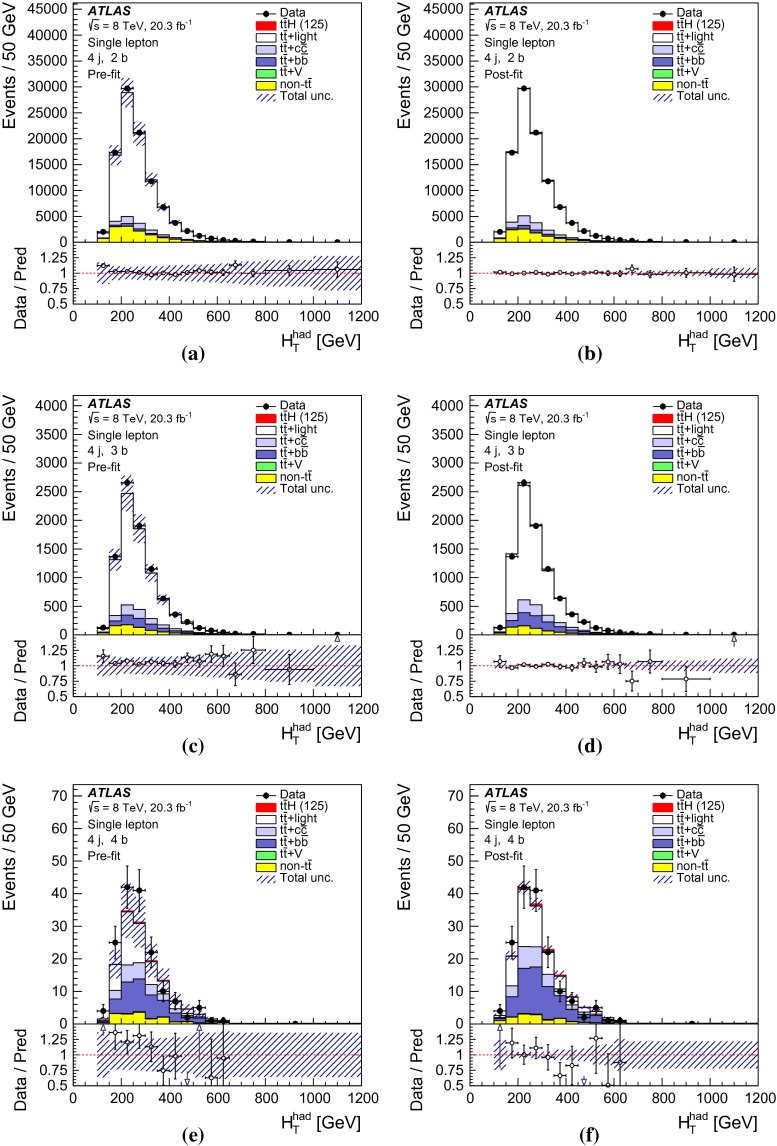
Single-lepton channel: comparison between data and prediction for the discriminant variable used in the $( 4 {\mathrm{j}},\,2 {\mathrm{b}})$ region **a** before the fit and **b** after the fit, in the $( 4 {\mathrm{j}},\,2 {\mathrm{b}})$ region **c** before the fit and **d** after the fit, in the $( 4 {\mathrm{j}},\,4 {\mathrm{b}})$ region **e** before the fit and **f** after the fit. The fit is performed on data under the signal-plus-background hypothesis. The last bin in all figures contains the overflow. The *bottom panel* displays the ratio of data to the total prediction. An *arrow* indicates that the point is off-scale. The *hashed area* represents the uncertainty on the background. The ${t\bar{t}H}$ signal yield (*solid*) is normalised to the SM cross section before the fit and to the fitted $\mu$ after the fit. In several regions, predominantly the control regions, the ${t\bar{t}H}$ signal yield is not visible on top of the large background

**Fig. 12:**
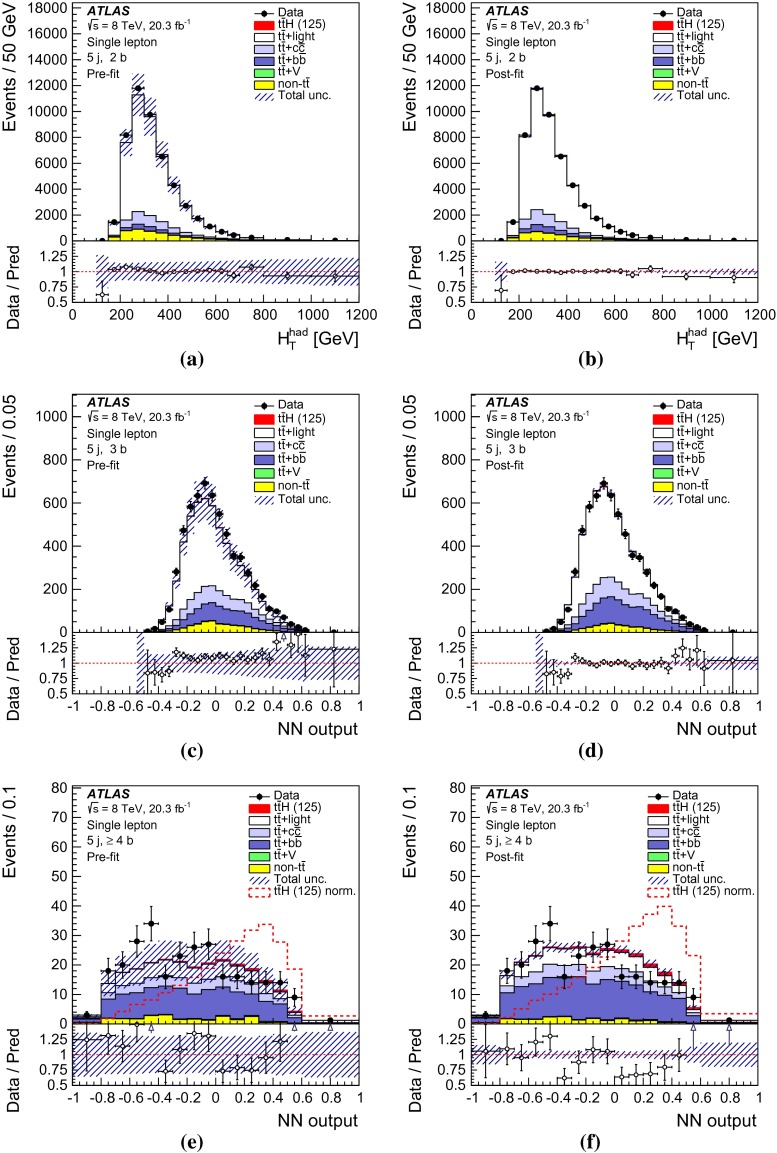
Single-lepton channel: comparison of data and prediction for the discriminant variable used in the $( 5 {\mathrm{j}},\,2 {\mathrm{b}})$ region **a** before the fit and **b** after the fit, in the $( 5 {\mathrm{j}},\,3 {\mathrm{b}})$ region **c** before the fit and **d** after the fit, in the $( 5 {\mathrm{j}},\ge \!{4} {\mathrm{b}})$ region **e** before the fit and **f** after the fit. The fit is peformed on data under the signal-plus-background hypothesis. The last bin in all figures contains the overflow. The *bottom panel* displays the ratio of data to the total prediction. An *arrow* indicates that the point is off-scale. The *hashed area* represents the uncertainty on the background. The *dashed line* shows ${t\bar{t}H}$ signal distribution normalised to background yield. The ${t\bar{t}H}$ signal yield (*solid*) is normalised to the SM cross section before the fit and to the fitted $\mu$ after the fit. In several regions, predominantly the control regions, the ${t\bar{t}H}$ signal yield is not visible on top of the large background

**Fig. 13:**
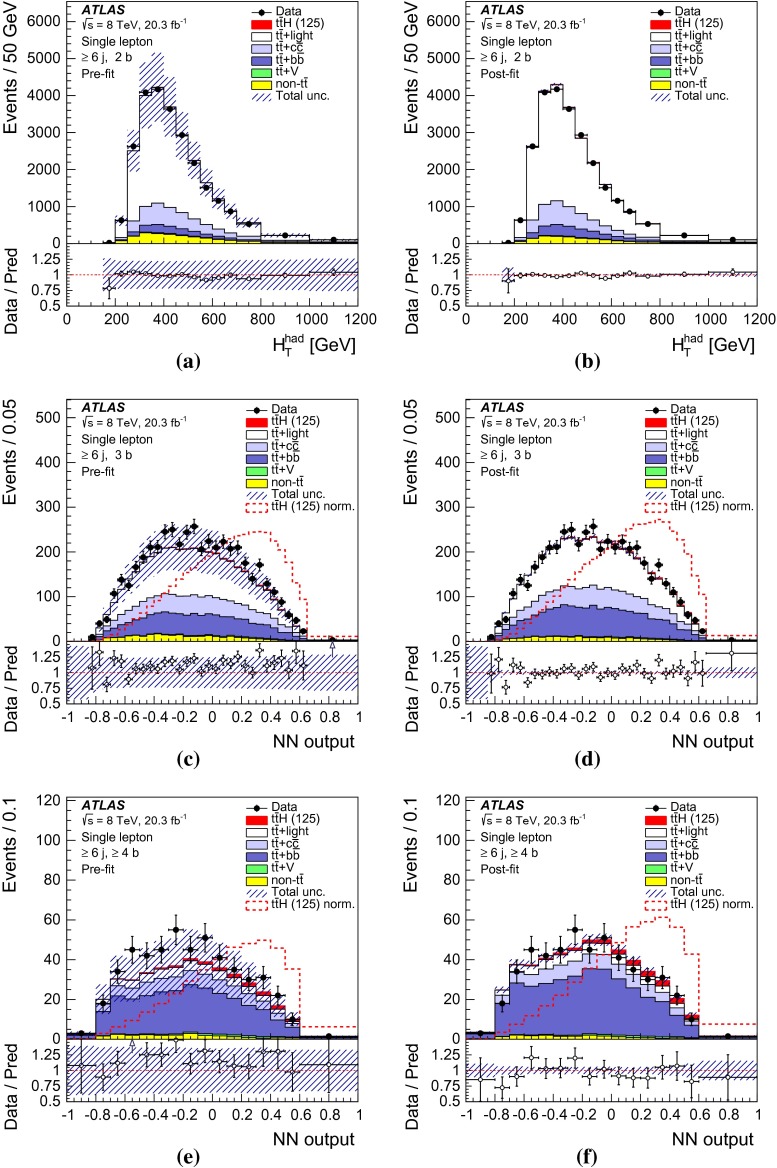
Single-lepton channel: comparison of data and prediction for the discriminant variable used in the $({\ge \!{6} {\mathrm{j}},\,2 {\mathrm{b}})}$ region **a** before the fit and **b** after the fit, in the ${(\ge \!{6} {\mathrm{j}},\,3 {\mathrm{b}})}$ region **c** before the fit and **d** after the fit, in the ${(\ge \!{6} {\mathrm{j}},\ge \!{4} {\mathrm{b}})}$ region **e** before the fit and **f** after the fit. The fit is performed on data under the signal-plus-background hypothesis. The last bin in all figures contains the overflow. The *bottom panel* displays the ratio of data to the total prediction. An *arrow* indicates that the point is off-scale. The *hashed area* represents the uncertainty on the background. The *dashed line* shows ${t\bar{t}H}$ signal distribution normalised to background yield. The ${t\bar{t}H}$ signal yield (*solid*) is normalised to the SM cross section before the fit and to the fitted $\mu$ after the fit. In several regions, predominantly the control regions, the ${t\bar{t}H}$ signal yield is not visible on top of the large background

**Fig. 14:**
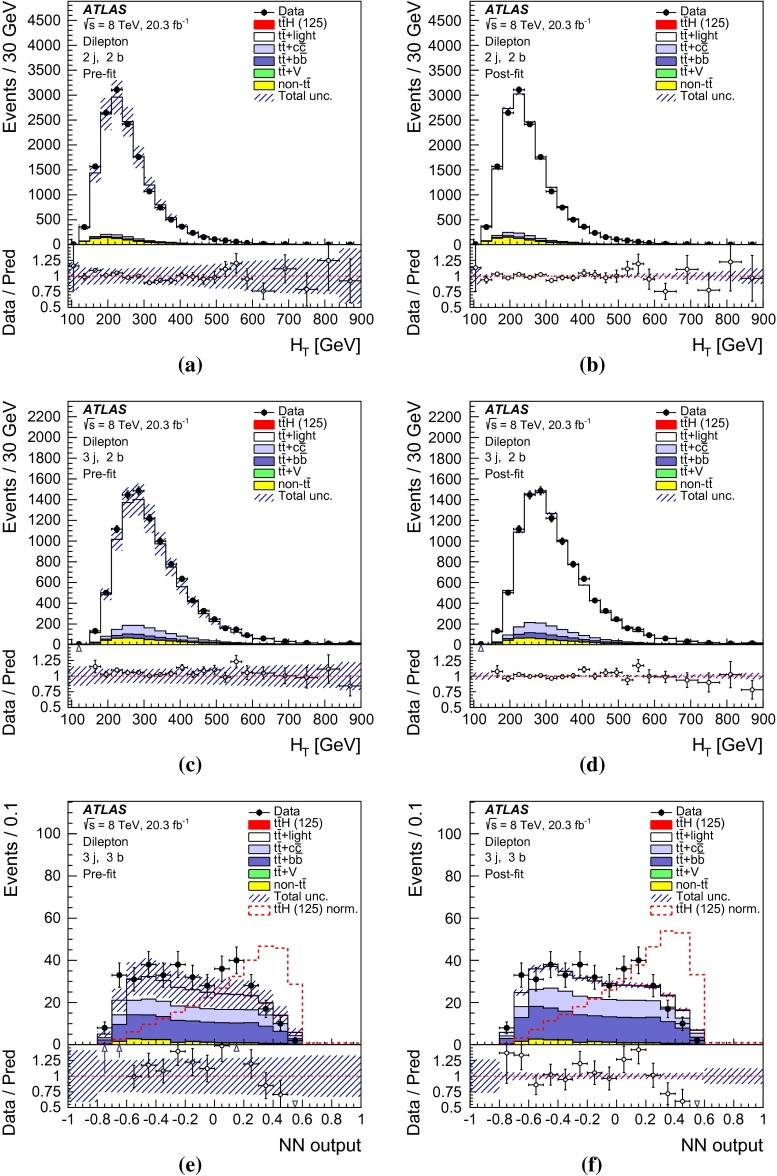
Dilepton channel: comparison of data and prediction for the discriminant variable used in the $(2 {\mathrm{j}},\,2 {\mathrm{b}})$ region **a** before the fit and **b** after the fit, in the $(3 {\mathrm{j}},\,2 {\mathrm{b}})$ region **c** before the fit and **d** after the fit, in the $(3 {\mathrm{j}},\,3 {\mathrm{b}})$ region **e** before the fit and **f** after the fit. The fit is performed on data under the signal-plus-background hypothesis. The last bin in all figures contains the overflow. The *bottom panel* displays the ratio of data to the total prediction. An *arrow* indicates that the point is off-scale. The *hashed area* represents the uncertainty on the background. The *dashed line* shows ${t\bar{t}H}$ signal distribution normalised to background yield. The ${t\bar{t}H}$ signal yield (*solid*) is normalised to the SM cross section before the fit and to the fitted $\mu$ after the fit. In several regions, predominantly the control regions, the ${t\bar{t}H}$ signal yield is not visible on top of the large background

**Fig. 15:**
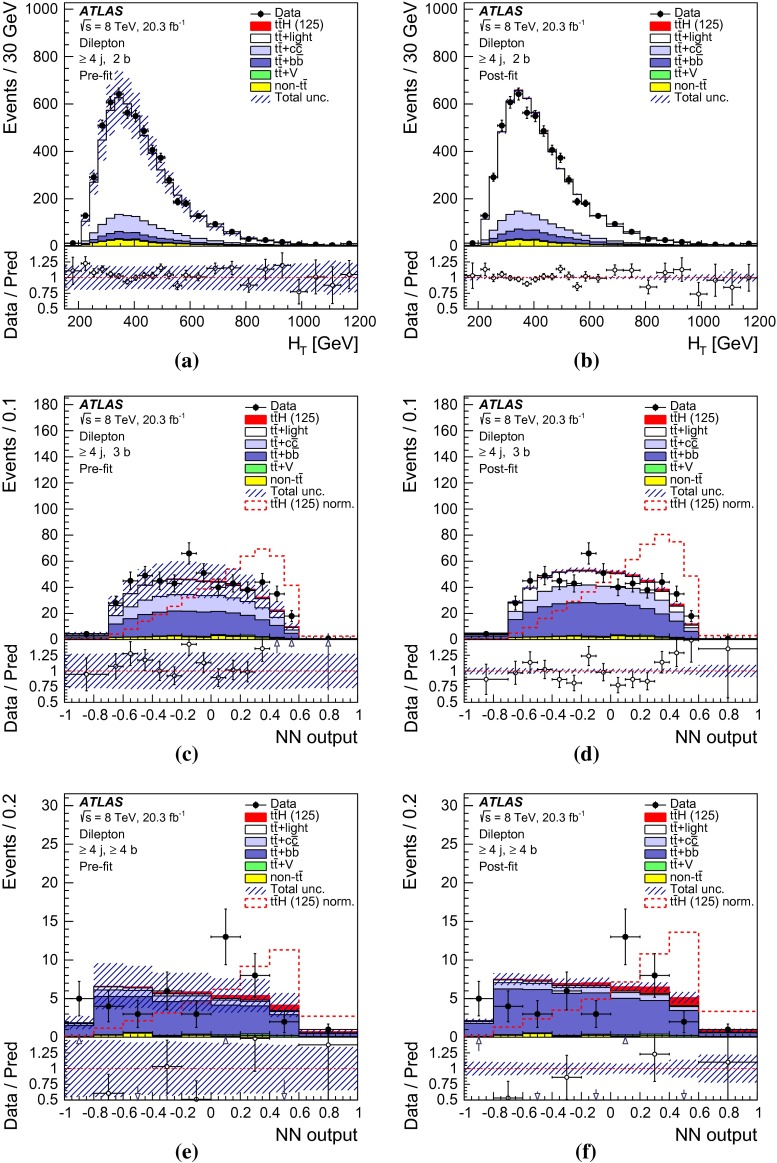
Dilepton channel: comparison of data and prediction for the discriminant variable used in the ${(\ge \!{4} {\mathrm{j}},\,2 {\mathrm{b}})}$ region **a** before the fit and **b** after the fit, in the ${(\ge \!{4} {\mathrm{j}},\,3 {\mathrm{b}})}$ region **c** before the fit and **d** after the fit, in the ${(\ge \!{4} {\mathrm{j}},\ge \!{4} {\mathrm{b}})}$ region **e** before the fit and **f** after the fit. The fit is performed on data under the signal-plus-background hypothesis. The last bin in all figures contains the overflow. The *bottom panel* displays the ratio of data to the total prediction. An *arrow* indicates that the point is off-scale. The *hashed area* represents the uncertainty on the background. The *dashed line* shows ${t\bar{t}H}$ signal distribution normalised to background yield. The ${t\bar{t}H}$ signal yield (*solid*) is normalised to the SM cross section before the fit and to the fitted $\mu$ after the fit. In several regions, predominantly the control regions, the ${t\bar{t}H}$ signal yield is not visible on top of the large background

Figures 11, 12, 13, 14 and 15 show a comparison of data and prediction for the discriminating variables (either ${{H_{\mathrm {T}}}^{\mathrm{had}}}$, ${{H_{\mathrm {T}}}}$, or NN discriminants) for each of the regions considered in the single-lepton and dilepton channels, respectively, both pre- and post-fit to data. The uncertainties decrease significantly in all regions due to constraints provided by data and correlations between different sources of uncertainty introduced by the fit to the data. In Appendix F, the most highly discriminating variables in the NN are shown post-fit compared to data.

Table 4 shows the observed $\mu$ values obtained from the individual fits in the single-lepton and dilepton channels, and their combination. The signal strength from the combined fit for $m_H=125{{\,\mathrm GeV\,}}$ is:

5 $$\begin{aligned} \mu (m_H=125{{\,\mathrm GeV\,}}) = 1.5 \pm 1.1. \end{aligned}$$

The expected uncertainty for the signal strength ($\mu ~=~1$) is $\pm 1.1$. The observed (expected) significance of the signal is 1.4 (1.1) standard deviations, which corresponds to an observed (expected) *p*-value of 8 % (15 %). The probability, *p*, to obtain a result at least as signal-like as observed if no signal is present is calculated using $q_0 = -2\mathrm{ln} (\mathcal {L}(0,\hat{\hat{\theta _{\mu }}})/\mathcal {L}(\hat{\mu }, \hat{\theta }))$ as a test statistic.

**Table 4:** The fitted values of signal strength and their uncertainties for the individual channels as well as their combination, assuming $m_H=125{{\,\mathrm GeV\,}}.$ Total uncertainties are shown

Signal strength	$\mu$	Uncertainty
Single lepton	1.2	1.3
Dilepton	2.8	2.0
Combination	1.5	1.1

**Fig. 16:**
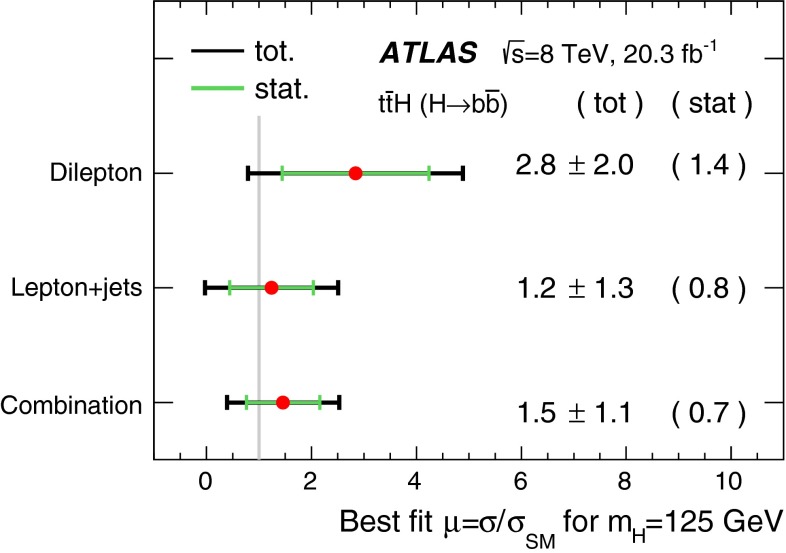
The fitted values of the signal strength and their uncertainties for the individual channels and their combination. The *green line* shows the statistical uncertainty on the signal strength

The fitted values of the signal strength and their uncertainties for the individual channels and their combination are shown in Fig. 16.

**Fig. 17:**
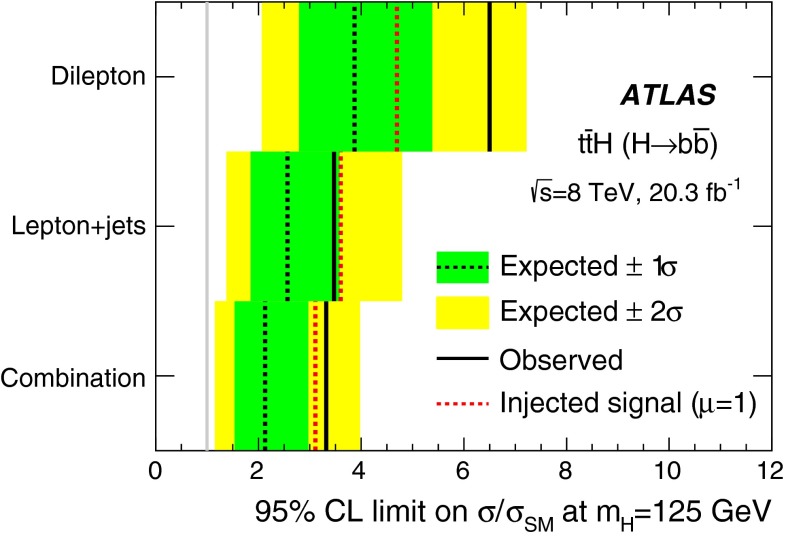
95 % CL upper limits on $\sigma (t\bar{t}H)$ relative to the SM prediction, $\sigma /\sigma _{\mathrm {SM}}$, for the individual channels as well as their combination. The observed limits (*solid lines*) are compared to the expected (median) limits under the background-only hypothesis and under the signal-plus-background hypothesis assuming the SM prediction for $\sigma (t\bar{t}H)$ and pre-fit prediction for the background. The surrounding *shaded bands* correspond to the 68 and 95 % confidence intervals around the expected limits under the background-only hypothesis, denoted by $\pm 1 \sigma$ and $\pm 2 \sigma$, respectively

The observed limits, those expected with and without assuming a SM Higgs boson with $m_H=125{{\,\mathrm GeV\,}}$, for each channel and their combination are shown in Fig. 17. A signal 3.4 times larger than predicted by the SM is excluded at 95 % CL using the CL$_\mathrm{{s}}$ method. A signal 2.2 times larger than for the SM Higgs boson is expected to be excluded in the case of no SM Higgs boson, and 3.1 times larger in the case of a SM Higgs boson. This is also summarised in Table 5.

**Table 5:** Observed and expected (median, for the background-only hypothesis) 95 % CL upper limits on $\sigma (t\bar{t}H)$ relative to the SM prediction, for the individual channels as well as their combination, assuming $m_H=125{{\,\mathrm GeV\,}}$. The 68 and 95 % confidence intervals around the expected limits under the background-only hypothesis are also provided, denoted by $\pm 1 \sigma$ and $\pm 2 \sigma$, respectively. The expected (median) 95 % CL upper limits assuming the SM prediction for $\sigma (t\bar{t}H)$ are shown in the last column

95 % CL upper limit	Observed	$-2\sigma$	$-1\sigma$	Median	$+1\sigma$	$+2\sigma$	Median ($\mu = 1$)
Single lepton	3.6	1.4	1.9	2.6	3.7	4.9	3.6
Dilepton	6.7	2.2	3.0	4.1	5.8	7.7	4.7
Combination	3.4	1.2	1.6	2.2	3.0	4.1	3.1

**Fig. 18:**
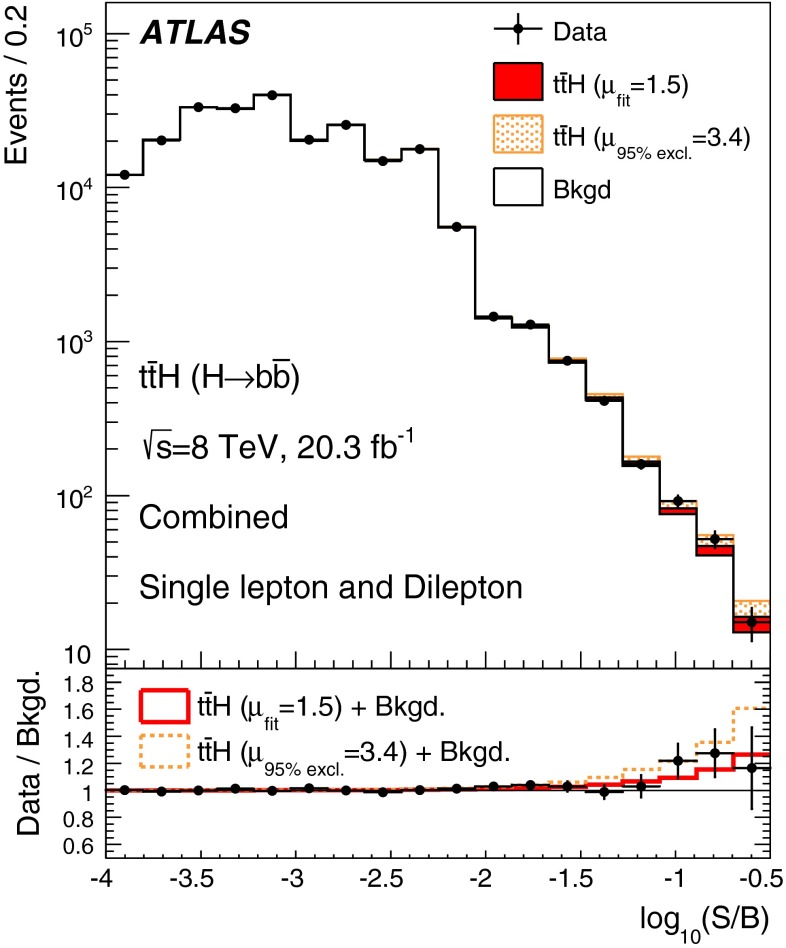
Event yields as a function of $\log _{10}(S/B)$, where *S* (signal yield) and *B* (background yield) are taken from the ${{H_{\mathrm {T}}}^{\mathrm{had}}}$, ${{H_{\mathrm {T}}}}$, and NN output bin of each event. Events in all fitted regions are included. The predicted background is obtained from the global signal-plus-background fit. The ${t\bar{t}H}$ signal is shown both for the best fit value ($\mu = 1.5$) and for the upper limit at 95 % CL ($\mu =3.4$)

Figure 18 summarises post-fit event yields as a function of $\log _{10}(S/B)$, for all bins of the distributions used in the combined fit of the single-lepton and dilepton channels. The value of $\log _{10}(S/B)$ is calculated according to the post-fit yields in each bin of the fitted distributions, either ${{H_{\mathrm {T}}}^{\mathrm{had}}}$, ${{H_{\mathrm {T}}}}$, or NN. The total number of background and signal events is displayed in bins of $\log _{10}(S/B)$. In particular, the last bin of Fig. 18 includes the two last bins from the most signal-rich region of the NN distribution in ${(\ge \!{6} {\mathrm{j}},\ge \!{4} {\mathrm{b}})}$ and the two last bins from the most signal-rich region of the NN in ${(\ge \!{4} {\mathrm{j}},\ge \!{4} {\mathrm{b}})}$ from the fit. The signal is normalised to the fitted value of the signal strength ($\mu = 1.5$) and the background is obtained from the global fit. A signal strength 3.4 times larger than predicted by the SM, which is excluded at 95 % CL by this analysis, is also shown.

**Fig. 19:**
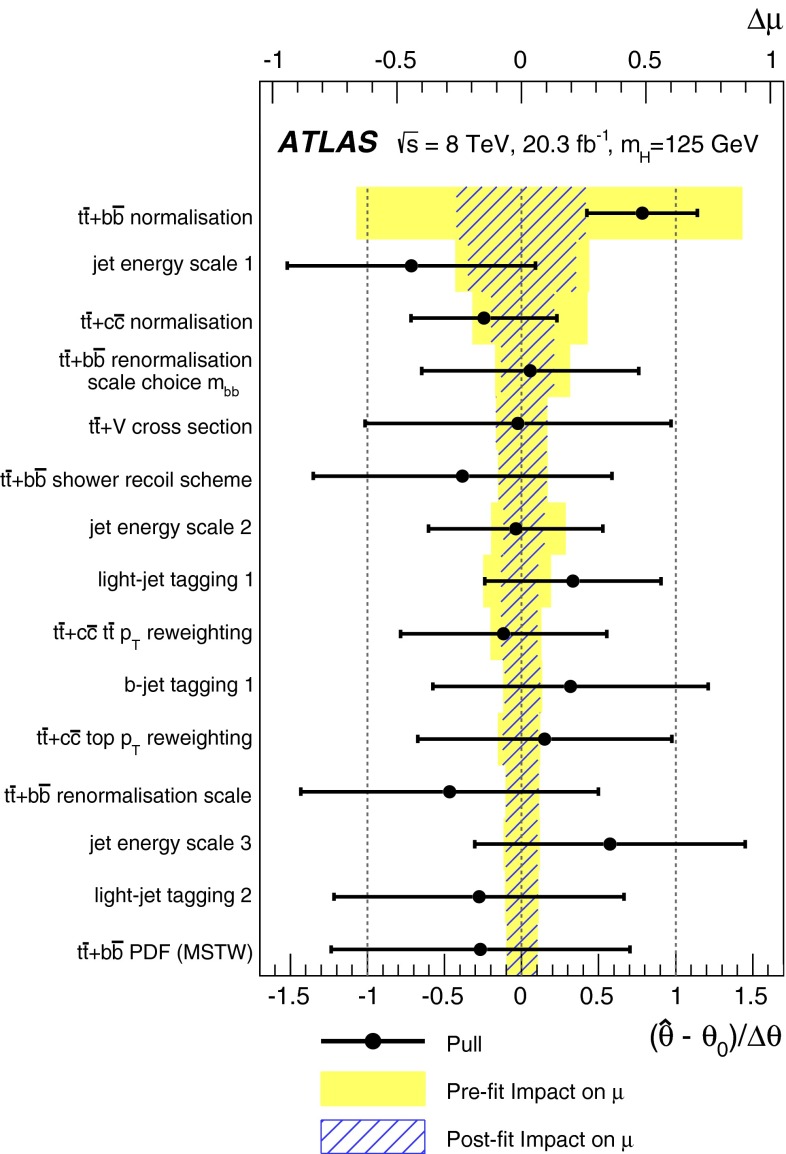
The fitted values of the nuisance parameters with the largest impact on the measured signal strength. The points, which are drawn conforming to the scale of the bottom axis, show the deviation of each of the fitted nuisance parameters, $\hat{{\theta }}$, from $\mathrm {\theta _{0}}$, which is the nominal value of that nuisance parameter, in units of the pre-fit standard deviation $\Delta \theta$. The *error bars* show the post-fit uncertainties, $\sigma _{\theta }$, which are close to 1 if the data do not provide any further constraint on that uncertainty. Conversely, a value of $\sigma _{\theta }$ much smaller than 1 indicates a significant reduction with respect to the original uncertainty. The nuisance parameters are sorted according to the post-fit effect of each on $\mu$ (*hashed blue area*) conforming to the scale of the top axis, with those with the largest impact at the top

**Fig. 20:**
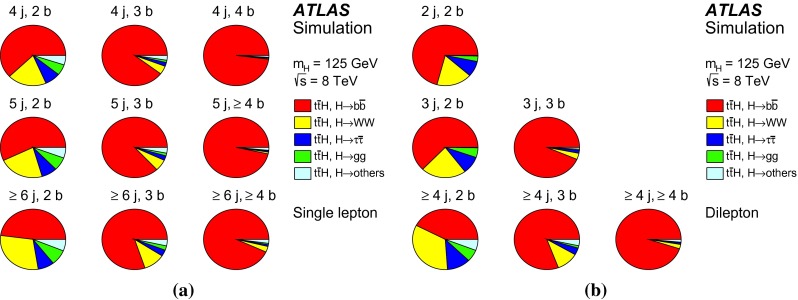
Contribution of various Higgs boson decay modes to the analysis regions in **a** the single-lepton channel and **b** the dilepton channel

Figure 19 demonstrates the effect of various systematic uncertainties on the fitted value of $\mu$ and the constraints provided by the data. The post-fit effect on $\mu$ is calculated by fixing the corresponding nuisance parameter at $\hat{{\theta }} \pm \sigma _{{\theta }}$, where $\hat{{\theta }}$ is the fitted value of the nuisance parameter and $\sigma _{{\theta }}$ is its post-fit uncertainty, and performing the fit again. The difference between the default and the modified $\mu$, $\Delta \mu$, represents the effect on $\mu$ of this particular systematic uncertainty. The largest effect arises from the uncertainty in normalisation of the irreducible ${t\bar{t}{+}b\bar{b}}$ background. This uncertainty is reduced by more than one half from the initial 50 %. The ${t\bar{t}{+}b\bar{b}}$ background normalisation is pulled up by about 40 % in the fit, resulting in an increase in the observed ${t\bar{t}{+}b\bar{b}}$ yield with respect to the Powheg+Pythia prediction. Most of the reduction in uncertainty on the ${t\bar{t}{+}b\bar{b}}$ normalisation is the result of the significant number of data events in the signal-rich regions dominated by ${t\bar{t}{+}b\bar{b}}$ background. With no Gaussian prior considered on the ${t\bar{t}{+}b\bar{b}}$ normalisation, as described in Sect. 8, the fit still prefers an increase in the amount of ${t\bar{t}{+}b\bar{b}}$ background by about 40 %.

The ${t\bar{t}{+}b\bar{b}}$ modelling uncertainties affecting the shape of this background also have a significant effect on $\mu$. These systematic uncertainties affect only the ${t\bar{t}{+}b\bar{b}}$ modelling and are not correlated with the other $t\bar{t}$+jets backgrounds. The largest of the uncertainties is given by the renormalisation scale choice. The uncertainty drastically changes the shape of the NN for the ${t\bar{t}{+}b\bar{b}}$ background, making it appear more signal-like.

The ${t\bar{t}{+}c\bar{c}}$ normalisation uncertainty is ranked third (Fig. 19) and its pull is slightly negative, while the post-fit yields for ${t\bar{t}{+}c\bar{c}}$ increase significantly in the four- and five-jet regions in the single-lepton channel and in the two- and three-jet regions of the dilepton channel (see Tables 10, 11 of Appendix 1). It was verified that this effect is caused by the interplay between the ${t\bar{t}{+}c\bar{c}}$ normalisation uncertainty and several other systematic uncertainties affecting the ${t\bar{t}{+}c\bar{c}}$ background yield.

The noticeable effect of the light-jet tagging (mistag) systematic uncertainty is explained by the relatively large fraction of the $t\bar{t}$+light background in the signal region with four *b*-jets in the single-lepton channel. The $t\bar{t}$+light events enter the 4-*b*-tag region through a mistag as opposed to the 3-*b*-tag region where tagging a *c*-jet from a *W* boson decay is more likely. Since the amount of data in the 4-*b*-tag regions is not large this uncertainty cannot be constrained significantly.

The $t\bar{t}+Z$ background with $Z \rightarrow b\bar{b}$ is an irreducible background to the ${t\bar{t}H}$ signal as it has the same number of *b*-jets in the final state and similar event kinematics. Its normalisation has a notable effect on $\mu$ ($\mathrm{d}\mu /\mathrm{d}\sigma ({t\bar{t}V}) = 0.3$) and the uncertainty arising from the $t\bar{t}{+}V$ normalisation cannot be significantly constrained by the fit. Other leading uncertainties include *b*-tagging and some components of the JES uncertainty.

Uncertainties arising from jet energy resolution, jet vertex fraction, jet reconstruction and JES that affect primarily low ${p_{\mathrm {T}}}$ jets as well as the $t\bar{t}$+light-jet background modelling uncertainties are constrained mainly in the signal-depleted regions. These uncertainties do not have a significant effect on the fitted value of $\mu$.

## Summary

A search has been performed for the Standard Model Higgs boson produced in association with a top-quark pair ($t\bar{t}H$) using 20.3 fb$^{-1}$ of *pp* collision data at $\sqrt{s}=8{{\,\mathrm TeV}}$ collected with the ATLAS detector during the first run of the Large Hadron Collider. The search focuses on ${H\rightarrow b\bar{b}}$ decays, and is performed in events with either one or two charged leptons.

To improve sensitivity, the search employs a likelihood fit to data in several jet and *b*-tagged jet multiplicity regions. Systematic uncertainties included in the fit are significantly constrained by the data. Discrimination between signal and background is obtained in both final states by employing neural networks in the signal-rich regions. In the single-lepton channel, discriminating variables are calculated using the matrix element technique. They are used in addition to kinematic variables as input to the neural network. No significant excess of events above the background expectation is found for a Standard Model Higgs boson with a mass of 125 GeV. An observed (expected) 95 % confidence-level upper limit of 3.4 (2.2) times the Standard Model cross section is obtained. By performing a fit under the signal-plus-background hypothesis, the ratio of the measured signal strength to the Standard Model expectation is found to be $\mu = 1.5 \pm 1.1$.

## Figures and Tables

**Fig. 21:**
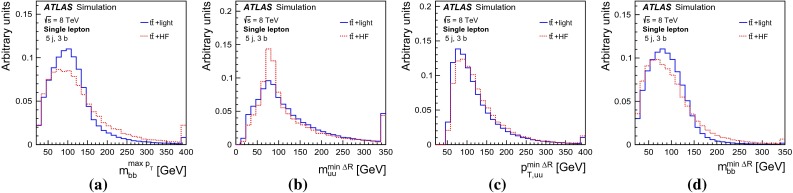
Single-lepton channel: comparison of $t\bar{t}$+HF (*dashed*) and $t\bar{t}$+light (*solid*) background for the four top-ranked input variables in the $( 5 {\mathrm{j}},\,3 {\mathrm{b}})$ region where the NN is designed to separate these two backgrounds. The plots include **a** ${m_{\mathrm{bb}}^{\mathrm{max\, p_{T}}}}$, **b** ${m^{\mathrm{min} \Delta \mathrm{R}}_{\mathrm{uu}}}$, **c** ${ p_{\mathrm{T, uu}}^{\mathrm{min\, \Delta R}}}$ and **d** ${m_{\mathrm{bb}}^{\mathrm{min} \Delta \mathrm{R}}}$

**Fig. 22:**
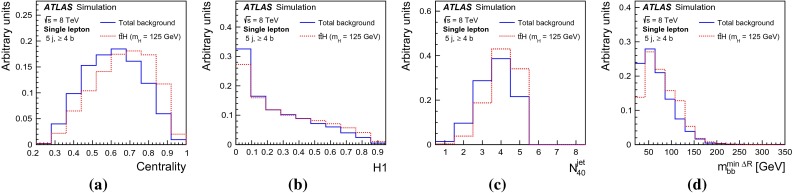
Single-lepton channel: comparison of ${t\bar{t}H}$ signal (*dashed*) and background (*solid*) for the four top-ranked input variables in the $( 5 {\mathrm{j}},\ge \!{4} {\mathrm{b}})$ region. The plots include **a** ${{\mathrm{Centrality}}}$, **b** *H*1, **c** ${N^{\mathrm{jet}}_{40}}$ and **d** ${m_{\mathrm{bb}}^{\mathrm{min} \Delta \mathrm{R}}}$

**Fig. 23:**
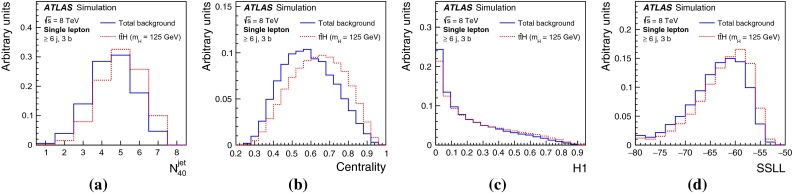
Single-lepton channel: comparison of ${t\bar{t}H}$ signal (*dashed*) and background (*solid*) for the four top-ranked input variables in the ${(\ge \!{6} {\mathrm{j}},\,3 {\mathrm{b}})}$ region. The plots include **a** ${N^{\mathrm{jet}}_{40}}$, **b** ${{\mathrm{Centrality}}}$, **c** *H*1, and **d** SSLL

**Fig. 24:**
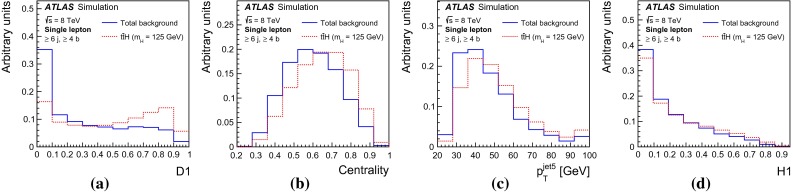
Single-lepton channel: comparison of ${t\bar{t}H}$ signal (*dashed*) and background (*solid*) for the four top-ranked input variables in the ${(\ge \!{6} {\mathrm{j}},\ge \!{4} {\mathrm{b}})}$ region. The plots include **a** *D*1, **b** ${{\mathrm{Centrality}}}$, **c** ${{p_{\mathrm {T}}}^{\mathrm{jet5}}}$, and **d** *H*1

**Fig. 25:**
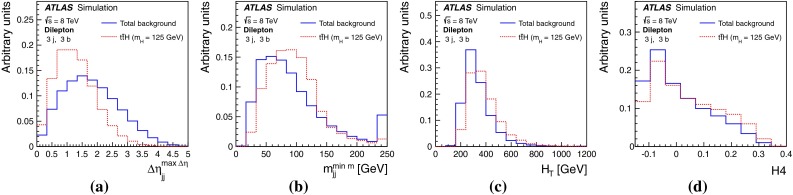
Dilepton channel: comparison of ${t\bar{t}H}$ signal (*dashed*) and background (*solid*) for the four top-ranked input variables in the $(3 {\mathrm{j}},\,3 {\mathrm{b}})$ region. The plots include **a** ${\Delta \eta _{\mathrm{jj}}^{\mathrm{max\,\Delta \eta }}}$, **b** ${m_{\mathrm{jj}}^{\mathrm{min\,m}}}$, **c** ${{H_{\mathrm {T}}}}$, and **d** *H*4

**Fig. 26:**
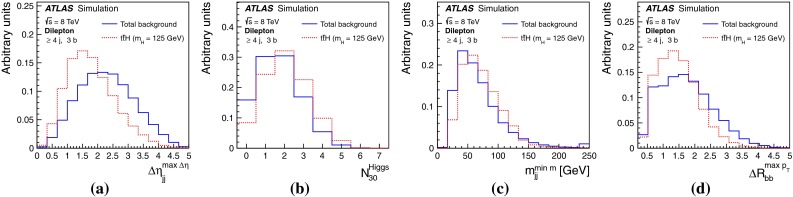
Dilepton channel: comparison of ${t\bar{t}H}$ signal (*dashed*) and background (*solid*) for the four top-ranked input variables in the ${(\ge \!{4} {\mathrm{j}},\,3 {\mathrm{b}})}$ region. The plots include **a** ${\Delta \eta _{\mathrm{jj}}^{\mathrm{max\,\Delta \eta }}}$, **b** ${\mathrm{{N_{30}^{Higgs}}}}$, **c** ${m_{\mathrm{jj}}^{\mathrm{min\,m}}}$, and **d** ${\Delta R_{\mathrm{bb}}^{\mathrm{max\, p_{T}}}}$

**Fig. 27:**
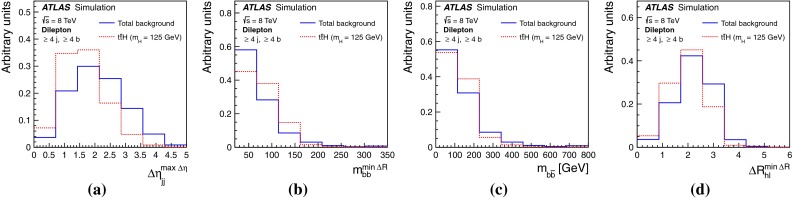
Dilepton channel: comparison of ${t\bar{t}H}$ signal (*dashed*) and background (*solid*) for the four top-ranked input variables in the ${(\ge \!{4} {\mathrm{j}},\ge \!{4} {\mathrm{b}})}$ region. The plots include **a** ${\Delta \eta _{\mathrm{jj}}^{\mathrm{max\,\Delta \eta }}}$, **b** ${m_{\mathrm{bb}}^{\mathrm{min} \Delta \mathrm{R}}}$, **c** ${m_{b\bar{b}}}$, and **d** ${\Delta R_{\mathrm{hl}}^{\mathrm{min\,\Delta R}}}$

**Fig. 28:**
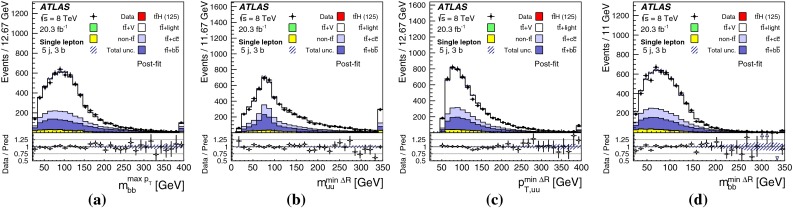
Single-lepton channel: post-fit comparison of data and prediction for the four top-ranked input variables in the $( 5 {\mathrm{j}},\,3 {\mathrm{b}})$ region. The plots include **a** ${m_{\mathrm{bb}}^{\mathrm{max\, p_{T}}}}$, **b** ${m^{\mathrm{min} \Delta \mathrm{R}}_{\mathrm{uu}}}$, **c** ${ p_{\mathrm{T, uu}}^{\mathrm{min\, \Delta R}}}$ and **d** ${m_{\mathrm{bb}}^{\mathrm{min} \Delta \mathrm{R}}}$. The first and last bins in all figures contain the underflow and overflow, respectively. The *bottom panel* displays the ratio of data to the total prediction. An *arrow* indicates that the point is off-scale. The *hashed area* represents the uncertainty on the background

**Fig. 29:**
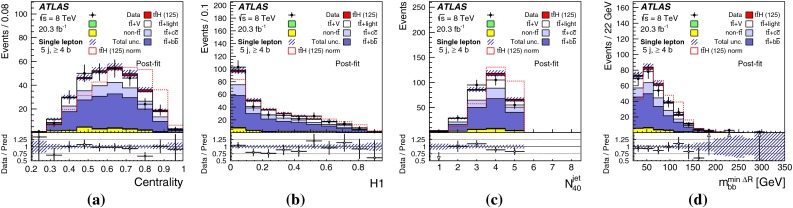
Single-lepton channel: post-fit comparison of data and prediction for the four top-ranked input variables in the $( 5 {\mathrm{j}},\ge \!{4} {\mathrm{b}})$ region. The plots include **a** ${{\mathrm{Centrality}}}$, **b** *H*1, **c** ${N^{\mathrm{jet}}_{40}}$ and **d** ${m_{\mathrm{bb}}^{\mathrm{min} \Delta \mathrm{R}}}$. The first and last bins in all figures contain the underflow and overflow, respectively. The *bottom panel* displays the ratio of data to the total prediction. An *arrow* indicates that the point is off-scale. The *hashed area* represents the uncertainty on the background. The *dashed line* shows ${t\bar{t}H}$ signal distribution normalised to background yield. The ${t\bar{t}H}$ signal yield (*solid*) is normalised to the fitted $\mu$

**Fig. 30:**
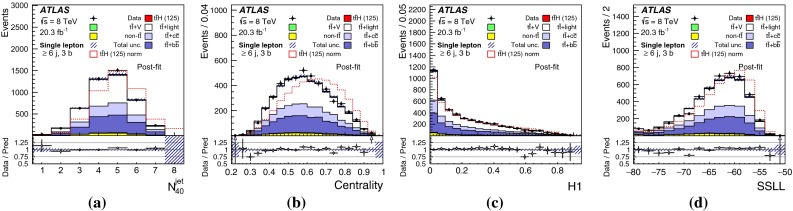
Single-lepton channel: post-fit comparison of data and prediction for the four top-ranked input variables in ${(\ge \!{6} {\mathrm{j}},\,3 {\mathrm{b}})}$ region. The plots include **a** ${N^{\mathrm{jet}}_{40}}$, **b** ${{\mathrm{Centrality}}}$, **c** *H*1, and **d** SSLL. The first and last bins in all figures contain the underflow and overflow, respectively. The *bottom panel* displays the ratio of data to the total prediction. An *arrow* indicates that the point is off-scale. The *hashed area* represents the uncertainty on the background. The *dashed line* shows ${t\bar{t}H}$ signal distribution normalised to background yield. The ${t\bar{t}H}$ signal yield (*solid*) is normalised to the fitted $\mu$

**Fig. 31:**
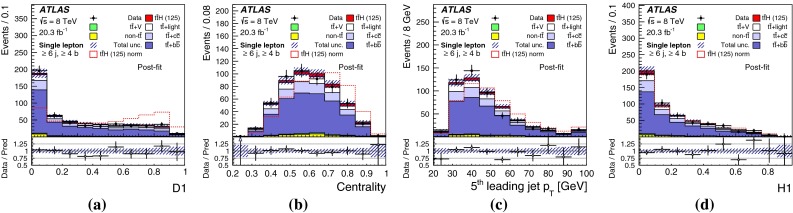
Single-lepton channel: post-fit comparison of data and prediction for the four top-ranked input variables in ${(\ge \!{6} {\mathrm{j}},\ge \!{4} {\mathrm{b}})}$ region. The plots include **a** *D*1, **b** ${{\mathrm{Centrality}}}$, **c** ${{p_{\mathrm {T}}}^{\mathrm{jet5}}}$, and **d** *H*1. The first and last bins in all figures contain the underflow and overflow, respectively. The *bottom panel* displays the ratio of data to the total prediction. An *arrow* indicates that the point is off-scale. The *hashed area* represents the uncertainty on the background. The *dashed line* shows ${t\bar{t}H}$ signal distribution normalised to background yield. The ${t\bar{t}H}$ signal yield (*solid*) is normalised to the fitted $\mu$

**Fig. 32:**
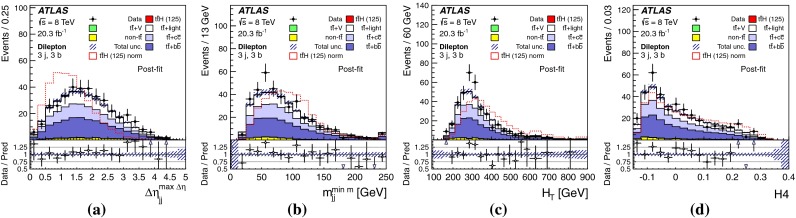
Dilepton channel: post-fit comparison of data and prediction for the four top-ranked input variables in the $(3 {\mathrm{j}},\,3 {\mathrm{b}})$ region. The plots include **a** ${\Delta \eta _{\mathrm{jj}}^{\mathrm{max\,\Delta \eta }}}$, **b** ${m_{\mathrm{jj}}^{\mathrm{min\,m}}}$, **c** ${{H_{\mathrm {T}}}}$, and **d** *H*4. The first and last bins in all figures contain the underflow and overflow, respectively. The *bottom panel* displays the ratio of data to the total prediction. An *arrow* indicates that the point is off-scale. The *hashed area* represents the uncertainty on the background. The *dashed line* shows ${t\bar{t}H}$ signal distribution normalised to background yield. The ${t\bar{t}H}$ signal yield (*solid*) is normalised to the fitted $\mu$

**Fig. 33:**
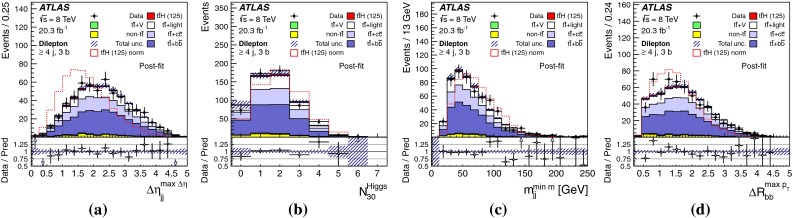
Dilepton channel: post-fit comparison of data and prediction for the four top-ranked input variables in the ${(\ge \!{4} {\mathrm{j}},\,3 {\mathrm{b}})}$ region. The plots include **a** ${\Delta \eta _{\mathrm{jj}}^{\mathrm{max\,\Delta \eta }}}$, **b** ${\mathrm{{N_{30}^{Higgs}}}}$, **c** ${m_{\mathrm{jj}}^{\mathrm{min\,m}}}$, and **d** ${\Delta R_{\mathrm{bb}}^{\mathrm{max\, p_{T}}}}$. The first and last bins in all figures contain the underflow and overflow, respectively. The *bottom panel* displays the ratio of data to the total prediction. An *arrow* indicates that the point is off-scale. The *hashed area* represents the uncertainty on the background. The *dashed line* shows ${t\bar{t}H}$ signal distribution normalised to background yield. The ${t\bar{t}H}$ signal yield (*solid*) is normalised to the fitted $\mu$

**Fig. 34:**
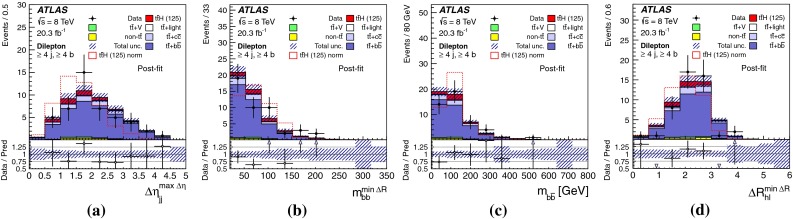
Dilepton channel: post-fit comparison of data and prediction for the four top-ranked input variables in the ${(\ge \!{4} {\mathrm{j}},\ge \!{4} {\mathrm{b}})}$ region. The plots include **a** ${\Delta \eta _{\mathrm{jj}}^{\mathrm{max\,\Delta \eta }}}$, **b** ${m_{\mathrm{bb}}^{\mathrm{min} \Delta \mathrm{R}}}$, **c** ${m_{b\bar{b}}}$, and **d** ${\Delta R_{\mathrm{hl}}^{\mathrm{min\,\Delta R}}}$. The first and last bins in all figures contain the underflow and overflow, respectively. The *bottom panel* displays the ratio of data to the total prediction. An *arrow* indicates that the point is off-scale. The *hashed area* represents the uncertainty on the background. The *dashed line* shows ${t\bar{t}H}$ signal distribution normalised to background yield. The ${t\bar{t}H}$ signal yield (*solid*) is normalised to the fitted $\mu$

## Appendix A: Higgs boson decay modes

Figure 20 shows the contributions of different Higgs boson decay modes in each of the analysis regions in the single-lepton and dilepton channels. The ${H\rightarrow b\bar{b}}$ decay is the dominant contribution in the signal-rich regions.

## Appendix B: Event yields prior to the fit

The event yields prior to the fit for the combined *e*+jets and $\mu$+jets samples for the different regions considered in the analysis are summarised in Table 6.

The event yields prior to the fit for the combined *ee*+jets, $\mu \mu$+jets and $e\mu$+jets samples for the different regions considered in the dilepton channel are summarised in Table 7.

**Table 6:** Single lepton channel: pre-fit event yields for signal, backgrounds and data in each of the analysis regions. The quoted uncertainties are the sum in quadrature of the statistical and systematic uncertainties on the yields

	4 j, 2 b	4 j, 3 b	4 j, 4 b
$t\bar{t}H$ (125)	31 $\pm$ 3	13 $\pm$ 2	2.0 $\pm$ 0.3
$t\bar{t}+$ light	77000 $\pm$ 7500	6200 $\pm$ 750	53 $\pm$ 12
$t\bar{t}+c\bar{c}$	4900 $\pm$ 3000	680 $\pm$ 390	21 $\pm$ 12
$t\bar{t}{+}b\bar{b}$	1800 $\pm$ 1100	680 $\pm$ 380	44 $\pm$ 25
*W*+jets	5100 $\pm$ 3000	220 $\pm$ 130	5.5 $\pm$ 3.3
*Z*+jets	1100 $\pm$ 600	50 $\pm$ 27	0.9 $\pm$ 0.6
Single top	4900 $\pm$ 640	340 $\pm$ 60	6.8 $\pm$ 1.6
Diboson	220 $\pm$ 71	11 $\pm$ 4.1	0.2 $\pm$ 0.1
$t\bar{t}{+}V$	120 $\pm$ 40	15 $\pm$ 5.1	0.9 $\pm$ 0.3
Lepton misID	1600 $\pm$ 620	100 $\pm$ 37	3.5 $\pm$ 1.3
Total	96000 $\pm$ 9500	8300 $\pm$ 1100	140 $\pm$ 34
Data	98049	8752	161
S/B	$<$0.001	0.002	0.014
S/$\sqrt{\mathrm{B}}$	0.099	0.141	0.167

	5 j, 2 b	5 j, 3 b	5 j, $\ge$4 b
$t\bar{t}H$ (125)	41 $\pm$ 2	23 $\pm$ 2	6.2 $\pm$ 0.8
$t\bar{t}+$ light	38000 $\pm$ 5500	3500 $\pm$ 520	61 $\pm$ 15
$t\bar{t}+c\bar{c}$	4300 $\pm$ 2400	810 $\pm$ 460	43 $\pm$ 25
$t\bar{t}{+}b\bar{b}$	1700 $\pm$ 880	890 $\pm$ 480	110 $\pm$ 63
*W*+jets	1900 $\pm$ 1200	140 $\pm$ 87	5.9 $\pm$ 3.9
*Z*+jets	410 $\pm$ 240	29 $\pm$ 17	1.5 $\pm$ 0.9
Single top	1900 $\pm$ 360	190 $\pm$ 41	8.3 $\pm$ 1.3
Diboson	97 $\pm$ 39	8.0 $\pm$ 3.4	0.4 $\pm$ 0.2
$t\bar{t}{+}V$	150 $\pm$ 48	26 $\pm$ 9	3.1 $\pm$ 1.0
Lepton misID	460 $\pm$ 170	70 $\pm$ 28	8.3 $\pm$ 3.7
Total	49000 $\pm$ 7000	5700 $\pm$ 980	250 $\pm$ 75
Data	49699	6199	286
S/B	0.001	0.004	0.025
S/$\sqrt{\mathrm{B}}$	0.186	0.301	0.397

	$\ge$6 j, 2 b	$\ge$6 j, 3 b	$\ge$6 j, $\ge$4 b
$t\bar{t}H$ (125)	64 $\pm$ 5	40 $\pm$ 3	16 $\pm$ 2
$t\bar{t}+$ light	19000 $\pm$ 4400	2000 $\pm$ 460	52 $\pm$ 17
$t\bar{t}+c\bar{c}$	3700 $\pm$ 2100	850 $\pm$ 480	79 $\pm$ 46
$t\bar{t}{+}b\bar{b}$	1400 $\pm$ 770	970 $\pm$ 530	250 $\pm$ 130
*W*+jets	910 $\pm$ 620	97 $\pm$ 66	8.6 $\pm$ 6.2
*Z*+jets	180 $\pm$ 120	19 $\pm$ 12	1.5 $\pm$ 1.0
Single top	840 $\pm$ 220	120 $\pm$ 35	12 $\pm$ 3.7
Diboson	50 $\pm$ 24	6.0 $\pm$ 3.0	0.5 $\pm$ 0.3
$t\bar{t}{+}V$	180 $\pm$ 59	45 $\pm$ 14	8.5 $\pm$ 2.8
Lepton misID	180 $\pm$ 66	21 $\pm$ 8	1.1 $\pm$ 0.5
Total	26000 $\pm$ 5800	4200 $\pm$ 1000	430 $\pm$ 150
Data	26185	4701	516
S/B	0.002	0.01	0.04
S/$\sqrt{\mathrm{B}}$	0.393	0.63	0.815

**Table 7:** Dilepton channel: pre-fit event yields for signal, backgrounds and data in each of the analysis regions. The quoted uncertainties are the sum in quadrature of the statistical and systematic uncertainties on the yields

	2 j, 2 b	3 j, 2 b	3 j, 3 b
$t\bar{t}H$ (125)	1.5 $\pm$ 0.2	5.3 $\pm$ 0.5	2.2 $\pm$ 0.3
$t\bar{t}+$ light	14000 $\pm$ 1800	8100 $\pm$ 880	96 $\pm$ 21
$t\bar{t}+c\bar{c}$	270 $\pm$ 170	600 $\pm$ 320	76 $\pm$ 44
$t\bar{t}{+}b\bar{b}$	150 $\pm$ 87	260 $\pm$ 130	120 $\pm$ 65
*Z*+jets	330 $\pm$ 30	190 $\pm$ 49	8.2 $\pm$ 3.1
Single top	430 $\pm$ 71	270 $\pm$ 30	7.6 $\pm$ 3.5
Diboson	6.8 $\pm$ 2.2	4.2 $\pm$ 1.5	$\le 0.1\pm$0.1
$t\bar{t}{+}V$	8.4 $\pm$ 2.7	21 $\pm$ 6	1.9 $\pm$ 0.6
Lepton misID	21 $\pm$ 10	33 $\pm$ 17	0.8 $\pm$ 0.4
Total	15000 $\pm$ 1900	9500 $\pm$ 1000	310 $\pm$ 85
Data	15296	9996	374
S/B	$<0.001$	0.001	0.006
S/$\sqrt{\mathrm{B}}$	0.012	0.053	0.114

	$\ge$4 j, 2 b	$\ge$4 j, 3 b	$\ge$4 j, $\ge$4 b
$t\bar{t}H$ (125)	15 $\pm$ 1	8.6 $\pm$ 0.6	2.7 $\pm$ 0.3
$t\bar{t}+$ light	4400 $\pm$ 810	120 $\pm$ 31	1.9 $\pm$ 0.8
$t\bar{t}+c\bar{c}$	710 $\pm$ 380	130 $\pm$ 74	5.0 $\pm$ 3.0
$t\bar{t}{+}b\bar{b}$	290 $\pm$ 150	200 $\pm$ 100	31 $\pm$ 17
*Z*+jets	100 $\pm$ 39	10 $\pm$ 4	0.6 $\pm$ 0.2
Single top	140 $\pm$ 55	11 $\pm$ 5	0.8 $\pm$ 0.2
Diboson	4.0 $\pm$ 1.3	0.4 $\pm$ 0.1	$\le 0.1\pm$0.1
$t\bar{t}{+}V$	45 $\pm$ 14	7.8 $\pm$ 2.4	1.1 $\pm$ 0.4
Lepton misID	38 $\pm$ 19	4.3 $\pm$ 2.2	0.4 $\pm$ 0.2
Total	5800 $\pm$ 1000	490 $\pm$ 140	43 $\pm$ 18
Data	6006	561	46
S/B	0.003	0.015	0.059
S/$\sqrt{\mathrm{{B}}}$	0.197	0.365	0.401

## Appendix C: Discrimination power of input variables

Figures 21, 22, 23, 24, 25, 26 and 27 show the discrimination between signal and background for the top four input variables in each region where NN is used in the single-lepton and dilepton channels, respectively. In Fig. 21, the NN is designed to separate $t\bar{t}$+HF from $t\bar{t}$+light.

## Appendix D: Tables of systematic uncertainties in the signal region

Tables 8 and 9 show pre-fit and post-fit contributions of the different categories of uncertainties (expressed in  %) for the ${t\bar{t}H}$ signal and main background processes in the ${(\ge \!{6} {\mathrm{j}},\ge \!{4} {\mathrm{b}})}$ region of the single-lepton channel and the ${(\ge \!{4} {\mathrm{j}},\ge \!{4} {\mathrm{b}})}$ region of the dilepton channel, respectively.

The “Lepton efficiency” category includes systematic uncertainties on electrons and muons listed in Table 3. The “Jet efficiency” category includes uncertainties on the jet vertex fraction and jet reconstruction. The “$$t\bar{t}$$ heavy-flavour modelling” category includes uncertainties on the $$t\bar{t}{+}b\bar{b}$$ NLO shape and on the $$t\bar{t}$$+$$c\bar{l}$$$${p_{\mathrm {T}}}$$ reweighting and generator. The “Theoretical cross sections” category includes uncertainties on the single top, diboson, *V*+jets and $$t\bar{t}{+}V$$ theoretical cross sections. The “$${t\bar{t}H}$$ modelling” category includes contributions from $${t\bar{t}H}$$ scale, generator, hadronisation model and PDF choice. The details of the evaluation of the uncertainties can be found in Sect. 8.

**Table 8:** Single lepton channel: normalisation uncertainties (expressed in %) on signal and main background processes for the systematic uncertainties considered, before and after the fit to data in ${(\ge \!{6} {\mathrm{j}},\ge \!{4} {\mathrm{b}})}$ region of the single lepton channel. The total uncertainty can be different from the sum in quadrature of individual sources due to the anti-correlations between them

	Pre-fit				Post-fit			
	$t\bar{t}H$ (125)	$t\bar{t}$ + light	$t\bar{t}+c\bar{c}$	$t\bar{t}{+}b\bar{b}$	$t\bar{t}H$ (125)	$t\bar{t}$ + light	$t\bar{t}+c\bar{c}$	$t\bar{t}{+}b\bar{b}$
$\ge$6 j, $\ge$4 b								
Luminosity	${\pm } 2.8$	${\pm } 2.8$	${\pm } 2.8$	${\pm } 2.8$	${\pm } 2.6$	${\pm } 2.6$	${\pm } 2.6$	${\pm } 2.6$
Lepton efficiencies	${\pm } 1.4$	${\pm } 1.4$	${\pm } 1.4$	${\pm } 1.5$	${\pm } 1.3$	${\pm } 1.3$	${\pm } 1.3$	${\pm } 1.3$
Jet energy scale	${\pm } 6.4$	${\pm } 13$	${\pm } 11$	${\pm } 9.2$	${\pm } 2.3$	${\pm } 5.3$	${\pm } 4.7$	${\pm } 3.6$
Jet efficiencies	${\pm } 1.7$	${\pm } 5.2$	${\pm } 2.7$	${\pm } 2.5$	${\pm } 0.7$	${\pm } 2.3$	${\pm } 1.2$	${\pm } 1.1$
Jet energy resolution	${\pm } 0.1$	${\pm } 4.4$	${\pm } 2.5$	${\pm } 1.6$	${\pm } 0.1$	${\pm } 2.3$	${\pm } 1.3$	${\pm } 0.8$
*b*-tagging efficiency	${\pm } 9.2$	${\pm } 5.6$	${\pm } 5.1$	${\pm } 9.3$	${\pm } 5.0$	${\pm } 3.1$	${\pm } 2.9$	${\pm } 5.0$
*c*-tagging efficiency	${\pm } 1.7$	${\pm } 6.0$	${\pm } 12$	${\pm } 2.4$	${\pm } 1.4$	${\pm } 5.1$	${\pm } 10$	${\pm } 2.1$
*l*-tagging efficiency	${\pm } 1.0$	${\pm } 19$	${\pm } 5.2$	${\pm } 2.1$	${\pm } 0.6$	${\pm } 11$	${\pm } 3.0$	${\pm } 1.1$
High ${p_{\mathrm {T}}}$ tagging efficiency	${\pm } 0.6$	–	${\pm } 0.7$	${\pm } 0.6$	${\pm } 0.3$	–	${\pm } 0.4$	${\pm } 0.3$
$t\bar{t}$: ${p_{\mathrm {T}}}$ reweighting	–	${\pm } 5.4$	${\pm } 6.1$	–	–	${\pm } 4.7$	${\pm } 5.4$	–
$t\bar{t}$: parton shower	–	${\pm } 13$	${\pm } 16$	${\pm } 11$	–	${\pm } 3.6$	${\pm } 10$	${\pm } 6.0$
$t\bar{t}$ + HF: normalisation	–	–	${\pm } 50$	${\pm } 50$	–	–	${\pm } 28$	${\pm } 14$
$t\bar{t}$ + HF: modelling	–	${\pm } 11$	${\pm } 16$	${\pm } 8.3$	–	${\pm } 3.6$	${\pm } 9.1$	${\pm } 7.1$
Theoretical cross sections	–	${\pm } 6.3$	${\pm } 6.3$	${\pm } 6.3$	–	${\pm } 4.1$	${\pm } 4.1$	${\pm } 4.1$
$t\bar{t}H$ modelling	${\pm } 2.7$	–	–	–	${\pm } 2.6$	–	–	–
Total	${\pm } 12$	${\pm } 32$	${\pm } 59$	${\pm } 54$	${\pm } 6.9$	${\pm } 9.2$	${\pm } 23$	${\pm } 12$

**Table 9:** Dilepton channel: normalisation uncertainties (expressed in % ) on signal and main background processes for the systematic uncertainties considered, before and after the fit to data in ${(\ge \!{4} {\mathrm{j}},\ge \!{4} {\mathrm{b}})}$ region of the dilepton channel. The total uncertainty can be different from the sum in quadrature of individual sources due to the anti-correlations between them

	Pre-fit				Post-fit			
	$t\bar{t}H$ (125)	$t\bar{t}$ + light	$t\bar{t}+c\bar{c}$	$t\bar{t}{+}b\bar{b}$	$t\bar{t}H$ (125)	$t\bar{t}$ + light	$t\bar{t}+c\bar{c}$	$t\bar{t}{+}b\bar{b}$
$\ge$4 j, $\ge$4 b								
Luminosity	${\pm } 2.8$	${\pm } 2.8$	${\pm } 2.8$	${\pm } 2.8$	${\pm } 2.6$	${\pm } 2.6$	${\pm } 2.6$	${\pm } 2.6$
Lepton efficiencies	${\pm } 2.5$	${\pm } 2.5$	${\pm } 2.5$	${\pm } 2.5$	${\pm } 1.8$	${\pm } 1.8$	${\pm } 1.8$	${\pm } 1.8$
Jet energy scale	${\pm } 4.5$	${\pm } 12$	${\pm } 9.4$	${\pm } 7.0$	${\pm } 2.0$	${\pm } 5.5$	${\pm } 4.5$	${\pm } 3.3$
Jet efficiencies	–	${\pm } 5.9$	${\pm } 1.6$	${\pm } 0.9$	–	${\pm } 2.6$	${\pm } 0.7$	${\pm } 0.4$
Jet energy resolution	${\pm } 0.1$	${\pm } 4.5$	${\pm } 1.1$	–	${\pm } 0.1$	${\pm } 2.3$	${\pm } 0.6$	–
*b*-tagging efficiency	${\pm } 10$	${\pm } 5.5$	${\pm } 5.4$	${\pm } 11$	${\pm } 5.6$	${\pm } 3.1$	${\pm } 3.0$	${\pm } 5.8$
*c*-tagging efficiency	${\pm } 0.5$	–	${\pm } 12$	${\pm } 0.6$	${\pm } 0.3$	–	${\pm } 10$	${\pm } 0.3$
*l*-tagging efficiency	${\pm } 0.7$	${\pm } 34$	${\pm } 7.0$	${\pm } 1.6$	${\pm } 0.4$	${\pm } 21$	${\pm } 4.2$	${\pm } 0.9$
High ${p_{\mathrm {T}}}$ tagging efficiency	–	–	${\pm } 0.6$	–	–	–	${\pm } 0.3$	–
$t\bar{t}$: ${p_{\mathrm {T}}}$ reweighting	–	${\pm } 5.8$	${\pm } 6.2$	–	–	${\pm } 5.0$	${\pm } 5.4$	–
$t\bar{t}$: parton shower	–	${\pm } 14$	${\pm } 18$	${\pm } 14$	–	${\pm } 4.8$	${\pm } 11$	${\pm } 8.1$
$t\bar{t}$ + HF: normalisation	–	–	${\pm } 50$	${\pm } 50$	–	–	${\pm } 28$	${\pm } 14$
$t\bar{t}$ + HF: modelling	–	${\pm } 11$	${\pm } 16$	${\pm } 12$	–	${\pm } 3.8$	${\pm } 10$	${\pm } 10$
Theoretical cross sections	–	${\pm } 6.3$	${\pm } 6.3$	${\pm } 6.2$	–	${\pm } 4.1$	${\pm } 4.1$	${\pm } 4.1$
$t\bar{t}H$ modelling	${\pm } 1.9$	–	–	–	${\pm } 1.8$	–	–	–
Total	${\pm } 12$	${\pm } 40$	${\pm } 59$	${\pm } 55$	${\pm } 6.7$	${\pm } 22$	${\pm } 22$	${\pm } 13$

## Appendix E: Post-fit event yields

The post-fit event yields for the combined single-lepton channel for the different regions considered in the analysis are summarised in Table 10. Similarly, the post-fit event yields for the combined dilepton channels for the different regions are summarised in Table 11.

**Table 10:** Dilepton channel: post-fit event yields under the signal-plus-background hypothesis for signal, backgrounds and data in each of the analysis regions. The quoted uncertainties are the sum in quadrature of statistical and systematic uncertainties on the yields, computed taking into account correlations among nuisance parameters and among processes

	4 j, 2 b	4 j, 3 b	4 j, 4 b
$t\bar{t}H$ (125)	48 $\pm$ 35	20 $\pm$ 15	3.0 $\pm$ 2.2
$t\bar{t}+$ light	78000 $\pm$ 1600	6300 $\pm$ 160	56 $\pm$ 5
$t\bar{t}+c\bar{c}$	6400 $\pm$ 1800	850 $\pm$ 220	26 $\pm$ 7
$t\bar{t}{+}b\bar{b}$	2500 $\pm$ 490	970 $\pm$ 150	63 $\pm$ 8
*W*+jets	3700 $\pm$ 1100	170 $\pm$ 51	4.0 $\pm$ 1.2
*Z*+jets	1100 $\pm$ 540	49 $\pm$ 25	1.1 $\pm$ 0.6
Single top	4700 $\pm$ 320	330 $\pm$ 28	6.8 $\pm$ 0.7
Diboson	220 $\pm$ 65	11 $\pm$ 4	0.3 $\pm$ 0.1
$t\bar{t}{+}V$	120 $\pm$ 38	16 $\pm$ 5	0.9 $\pm$ 0.3
Lepton misID	1100 $\pm$ 370	78 $\pm$ 26	2.6 $\pm$ 1.0
Total	98000 $\pm$ 340	8800 $\pm$ 82	160 $\pm$ 6
Data	98049	8752	161

	5 j, 2 b	5 j, 3 b	5 j, $\ge$4 b
$t\bar{t}H$ (125)	60 $\pm$ 44	34 $\pm$ 25	9.4 $\pm$ 6.9
$t\bar{t}+$ light	38000 $\pm$ 1000	3600 $\pm$ 120	65 $\pm$ 6
$t\bar{t}+c\bar{c}$	4800 $\pm$ 1200	930 $\pm$ 230	51 $\pm$ 12
$t\bar{t}{+}b\bar{b}$	2400 $\pm$ 360	1300 $\pm$ 180	150 $\pm$ 20
*W*+jets	1200 $\pm$ 420	87 $\pm$ 31	4.0 $\pm$ 1.5
*Z*+jets	370 $\pm$ 200	28 $\pm$ 16	1.4 $\pm$ 0.8
Single top	1700 $\pm$ 150	190 $\pm$ 18	8.2 $\pm$ 0.7
Diboson	94 $\pm$ 35	8.0 $\pm$ 3.1	0.5 $\pm$ 0.2
$t\bar{t}{+}V$	140 $\pm$ 43	26 $\pm$ 8	3.2 $\pm$ 1.0
Lepton misID	340 $\pm$ 110	44 $\pm$ 16	5.7 $\pm$ 2.2
Total	50000 $\pm$ 220	6200 $\pm$ 54	300 $\pm$ 10
Data	49699	6199	286

	$\ge$6 j, 2 b	$\ge$6 j, 3 b	$\ge$6 j, $\ge$4 b
$t\bar{t}H$ (125)	89 $\pm$ 65	57 $\pm$ 42	24 $\pm$ 17
$t\bar{t}+$ light	19000 $\pm$ 700	2100 $\pm$ 87	58 $\pm$ 5
$t\bar{t}+c\bar{c}$	3700 $\pm$ 890	890 $\pm$ 210	85 $\pm$ 21
$t\bar{t}{+}b\bar{b}$	2000 $\pm$ 310	1400 $\pm$ 190	330 $\pm$ 37
*W*+jets	450 $\pm$ 170	51 $\pm$ 19	4.4 $\pm$ 1.9
*Z*+jets	150 $\pm$ 86	16 $\pm$ 9	1.2 $\pm$ 0.7
Single top	730 $\pm$ 83	110 $\pm$ 14	11 $\pm$ 2
Diboson	45 $\pm$ 20	5.6 $\pm$ 2.6	0.5 $\pm$ 0.2
$t\bar{t}{+}V$	170 $\pm$ 52	42 $\pm$ 13	8.2 $\pm$ 2.5
Lepton misID	120 $\pm$ 41	14 $\pm$ 5	1.1 $\pm$ 0.5
Total	26000 $\pm$ 160	4600 $\pm$ 55	520 $\pm$ 18
Data	26185	4701	516

**Table 11:** Single lepton channel: post-fit event yields under the signal-plus-background hypothesis for signal, backgrounds and data in each of the analysis regions. The quoted uncertainties are the sum in quadrature of statistical and systematic uncertainties on the yields, computed taking into account correlations among nuisance parameters and among processes

	2 j, 2 b	3 j, 2 b	3 j, 3 b
$t\bar{t}H$ (125)	2.4 $\pm$ 1.8	8.1 $\pm$ 5.9	3.0 $\pm$ 2.2
$t\bar{t}+$ light	14000 $\pm$ 160	8300 $\pm$ 170	84 $\pm$ 9.6
$t\bar{t}+c\bar{c}$	400 $\pm$ 110	700 $\pm$ 160	92 $\pm$ 22
$t\bar{t}{+}b\bar{b}$	190 $\pm$ 36	350 $\pm$ 49	140 $\pm$ 19
*Z*+jets	330 $\pm$ 22	200 $\pm$ 43	7.3 $\pm$ 2.4
Single top	430 $\pm$ 35	260 $\pm$ 21	7.6 $\pm$ 1.5
Diboson	6.8 $\pm$ 2.1	4.5 $\pm$ 1.4	$\le 0.1\pm 0.1$
$t\bar{t}{+}V$	8.7 $\pm$ 2.7	21 $\pm$ 6	1.8 $\pm$ 0.6
Lepton misID	19 $\pm$ 10	30 $\pm$ 15	1.7 $\pm$ 0.4
Total	15000 $\pm$ 120	9900 $\pm$ 82	340 $\pm$ 14
Data	15296	9996	374

	$\ge$4 j, 2 b	$\ge$4 j, 3 b	$\ge$4 j, $\ge$4 b
$t\bar{t}H$ (125)	22 $\pm$ 16	11 $\pm$ 8	3.1 $\pm$ 2.3
$t\bar{t}+$ light	4500 $\pm$ 150	100 $\pm$ 12	1.4 $\pm$ 0.3
$t\bar{t}+c\bar{c}$	740 $\pm$ 170	140 $\pm$ 30	4.8 $\pm$ 1.1
$t\bar{t}{+}b\bar{b}$	370 $\pm$ 59	230 $\pm$ 31	30 $\pm$ 4
*Z*+jets	100 $\pm$ 33	9.5 $\pm$ 3.1	0.4 $\pm$ 0.2
Single top	140 $\pm$ 23	11 $\pm$ 2	0.6 $\pm$ 0.1
Diboson	4.2 $\pm$ 1.3	0.3 $\pm$ 0.1	$\le$0.1 $\pm$ 0.1
$t\bar{t}{+}V$	43 $\pm$ 13	7.0 $\pm$ 2.1	0.9 $\pm$ 0.3
Lepton misID	34 $\pm$ 18	3.5 $\pm$ 1.8	0.2 $\pm$ 0.1
Total	5900 $\pm$ 65	520 $\pm$ 18	42 $\pm$ 4
Data	6006	561	46

## Appendix F: Post-fit input variables

Figures 28, 29, 30, 31, 32, 33 and 34 show a comparison of data and prediction for the top four input variables in each region with a neural network in the single-lepton channel and dilepton channel, respectively. All of the plots are made using post-fit predictions.

## Footnotes

1 ATLAS uses a right-handed coordinate system with its origin at the nominal interaction point (IP) in the centre of the detector and the *z*-axis coinciding with the axis of the beam pipe. The *x*-axis points from the IP to the centre of the LHC ring, and the *y*-axis points upward. Cylindrical coordinates (*r*, $\phi$) are used in the transverse plane, $\phi$ being the azimuthal angle around the beam pipe. The pseudorapidity is defined in terms of the polar angle $\theta$ as $\eta = - \ln \tan (\theta /2)$. Transverse momentum and energy are defined as ${p_{\mathrm {T}}}= \text { p} \sin \theta$ and ${E_{\mathrm {T}}}= \text { E} \sin \theta$, respectively.
